# Nanozyme-reinforced hydrogel as a H_2_O_2_-driven oxygenerator for enhancing prosthetic interface osseointegration in rheumatoid arthritis therapy

**DOI:** 10.1038/s41467-022-34481-5

**Published:** 2022-11-09

**Authors:** Yue Zhao, Shanliang Song, Dongdong Wang, He Liu, Junmin Zhang, Zuhao Li, Jincheng Wang, Xiangzhong Ren, Yanli Zhao

**Affiliations:** 1 International Joint Research Center for Molecular Science, College of Chemistry and Environmental Engineering, Shenzhen University, Shenzhen, 518060 China; 2 School of Chemistry, Chemical Engineering and Biotechnology, Nanyang Technological University, 21 Nanyang Link, Singapore, 637371 Singapore; 3 Orthopaedic Medical Center, The Second Hospital of Jilin University, Orthopaedic Research Institute of Jilin Province, Changchun, 130041 China

**Keywords:** Stem-cell biotechnology, Implants, Biomedical materials, Metal-organic frameworks

## Abstract

Stem cell-based therapy has drawn attention for enhancing the osseointegration efficiency after joint replacement in the rheumatoid arthritis (RA). However, therapeutic efficacy of this approach is threatened by the accumulated reactive oxygen species (ROS) and poor oxygen supply. Herein, we develop a nanozyme-reinforced hydrogel for reshaping the hostile RA microenvironment and improving prosthetic interface osseointegration. The engineered hydrogel not only scavenges endogenously over-expressed ROS, but also synergistically produces dissolved oxygen. Such performance enables the hydrogel to be utilized as an injectable delivery vehicle of bone marrow-derived mesenchymal stem cells (BMSCs) to protect implanted cells from ROS and hypoxia-mediated death and osteogenic limitation. This nanozyme-reinforced hydrogel encapsulated with BMSCs can alleviate the symptoms of RA, including suppression of local inflammatory cytokines and improvement of osseointegration. This work provides a strategy for solving the long-lasting challenge of stem cell transplantation and revolutionizes conventional intervention methods for improving prosthetic interface osseointegration in RA.

## Abstract (web summary)

Stem cell transplantation has huge potential but can suffer from effects caused by disease state microenvironment. Here, the authors report on the development of a nanozyme reinforced hydrogel for reactive oxygen species scavenging and the generation of dissolved oxygen, demonstrating improved osteointegration using the hydrogel.

## Introduction

Rheumatoid arthritis (RA) is one of the most widespread and devastating autoimmune disorders^1,2^. It is primarily characterized by the inflammation of the synovial membrane that further progresses and induces joint pain, stiffness, loss of function, and in some cases limited mobility ultimately leading to joint replacement surgery to restore function and relieve pain. RA carries tremendous social tribulations and ever-increasing economic hardship for patients^3,4^. While several therapeutic methods have been exploited, the full recovery or resolution of hostile RA microenvironment is still far from enough satisfactory^5,6^. Recently, stem cell-based therapies have emerged as a promising alternative for RA management, which presents many important advantages over traditional interventional or drug treatments^7,8^. Particularly, bone marrow-derived mesenchymal stem cells (BMSCs), known as one of multipotent progenitor cells, have superior abilities to differentiate into chondrocytes and osteoblasts. For joint replacement surgery in RA, BMSCs have demonstrated the ability to improve osseointegration, thus avoiding postoperative complications such as prosthesis loosening and displacement. Another important character of BMSCs is their potent anti-inflammatory and immunomodulatory properties^9^. Immune-regulatory effect of stem cells mainly originates from the secretion of a variety of immune-related bioactive factors such as cytokines, chemokines, exosomes, growth factors, and extracellular vesicles. Having the above-mentioned osteogenic and immune-regulatory capacities, BMSCs display a remarkable therapeutic effect for RA by amelioration of insufficient bone regeneration and inflammatory reaction.

While stem cell-based therapy exhibits advantages for RA management, the wide application of this method still faces some insurmountable obstacles. One of the obstacles to achieve effective treatment is the hypoxic microenvironment of RA. Increasing evidence suggests that fibroblast-like hyperplastic synoviocytes and infiltrated inflammatory cells dramatically contribute to the increased oxygen consumption in the RA synovium^10,11^. Meanwhile, the highly dysregulated architecture of the microvasculature limits the delivery of oxygen to the synovium, thereby exacerbating the hypoxic condition^12,13^. The uncontrolled overproduction of reactive oxygen species (ROS) is another hallmark of RA. Under the hostile RA microenvironment, the poor O_2_ supply leads to a significant destruction of cellular metabolism and mitochondrial function, which inevitably increases the accumulation of ROS and inflammatory cytokines^14^. The uncontrolled accumulation of ROS together with the hypoxic condition result in the devastating destruction of transplanted stem cells, and thus dramatically compromise the therapeutic effect. Considering the magnitude of these problems, essential effort should be further devoted to the development of a desirable cell delivery vehicle with the capability of synergistically scavenging ROS and producing O_2_ to boost the stem cell therapy efficiency around the prosthetic interface in the RA microenvironment.

Hydrogels, acknowledged as cross-linked 3D polymer networks, have attracted substantial attention as effective vehicles to deliver bioactive substances (e.g., drugs and stem cells) in therapeutics of various disorders and reconstruction of tissue functions^15,16^. Hydrogels possess high-water content, tailorable structure, good biocompatibility, predictable degradation rates, and adjustable mechanical strength^17,18^. These beneficial intrinsic properties of hydrogels enable them to recapitulate the temporal and spatial complexity of the native extracellular matrix, which are analogous not only in formula but also in physicochemical parameters^19,20^. In addition, a variety of specific cues and triggers can be introduced into hydrogels for regulation of encapsulated cell metabolism, survival, cell-to-cell interactions, and differentiation^21,22^. Thus, hydrogels have become a frontier in cell delivery by virtue of their irreplaceable superiorities over other currently available toolset of biomaterials. Even so, the hydrogel vehicle choices for stem cell-based RA therapy are very limited, since most of the reported hydrogels have invariably been focused on delivery functions while never taking both hypoxia and ROS in the RA synovium into consideration. On the other side, recent advances in nanozyme represent a promising solution for reshaping oxidative stress and hypoxia in various oxidative stress-associated diseases^23,24^. The introduction of nanozymes into hydrogels can yield highly advanced bioactive platforms that tackle complex tissue-specific physiology for expanding the range of biomedical applications. Therefore, we hypothesize that the integration of hydrogels and nanozyme can offer a more rational and targeted tactic to satisfy the multifunction of ROS decomposition and sustained oxygenation, and provide a suitable microenvironment to enhance cell viability as well as direct stem cell differentiation toward the desired lineages.

In this work, we develop nanozyme-reinforced hydrogels as H_2_O_2_-driven oxygenerators to regulate stem cell behavior. The biological metabolism-inspired hydrogel is derived from a dynamic cross-linked natural polymer and a catalase-mimic nanozyme, which exhibits injectable, self-healing, and biocompatible properties. More significantly, benefitting from the inclusion of catalytic nanozyme, the obtained hydrogel system can effectively decompose the endogenous H_2_O_2_ to produce O_2_. The corresponding in vitro experiments demonstrate that the nanozyme-reinforced hydrogel successfully assuages the hypoxic and oxidative microenvironment of RA, thereby providing an appropriate 3D microenvironment for BMSC proliferation and osteogenesis. To further investigate the efficiency of stem cell therapy in vivo, the 3D printed titanium alloy scaffold modified with BMSC-encapsulated hydrogel is transplanted into the large-scale bone defects of an RA animal model. The nanozyme-reinforced hydrogel with stem cells can effectively suppress inflammatory cytokines and improve prosthetic interface osseointegration. These findings reveal that the engineered hydrogel scaffold holds a great promise for the improvement of stem cell therapy efficacy in RA.

## Results

### Design and synthesis of nanozyme-reinforced hydrogel

In our previous studies, we demonstrated that the mesoporous manganese cobalt oxide (Mn_1.8_Co_1.2_O_4_ = MnCoO) nanozyme derived from Mn_3_[Co(CN)_6_]_2_ metal-organic frameworks (MOFs) simultaneously exhibited endogenous H_2_O_2_ decomposition and oxygen generation abilities for amplified photodynamic therapy^25^. Inspired by this finding, the MnCoO nanozyme was employed as the building block of hydrogel and behaved in the catalase-mimic enzyme activity in the three-dimensional polymer network. The structure and morphology of the prepared nanozyme (Supplementary Figs. 1–9) were studied by scanning electron microscopy (SEM), powder X-ray diffraction (XRD), and X-ray photoelectron spectroscopy (XPS), and all the results agreed well with the literature report^25^. Then, the amino group (-NH_2_)-rich ε-polylysine was modified on the surface of MnCoO nanozyme via a facile electrostatic assembly to obtain ε-PLE@MnCoO nanoparticles, which were characterized by the zeta potential measurement (Supplementary Fig. 10) and Fourier transform infrared (FT-IR) spectroscopy (Supplementary Fig. 11). Moreover, the polymer used in this study was originated from hyaluronic acid (HA). HA is a polysaccharide naturally found in synovial joint fluids, which was employed as the hydrogel backbone on account of its biocompatibility, biodegradability, bio-functionality, high-water retention, and viscoelastic properties^26^. We then synthesized the hydrazide group (-CONH-NH_2_) and aldehyde group (-CHO)-functionalized HA through representative chemical reactions, respectively (Supplementary Fig. 12).

**Fig. 1:**
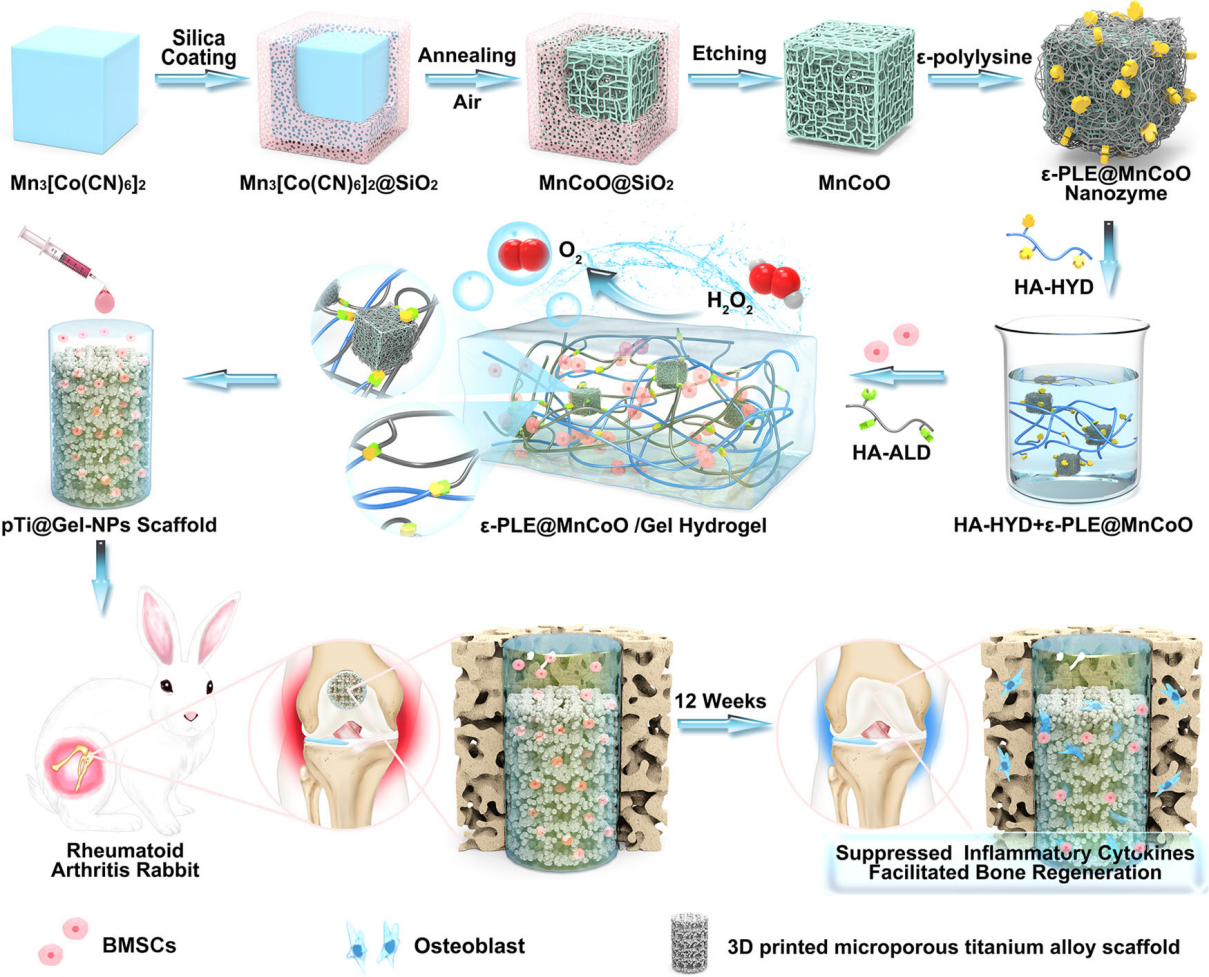
Development of nanozyme-reinforced hydrogels as H_2_O_2_-driven oxygenerators to regulate stem cell behavior. Schematic diagram illustrating the synthesis process of nanozyme-reinforced self-protecting hydrogel and its application for enhancing prosthetic interface osseointegration in RA therapy.

**Fig. 2:**
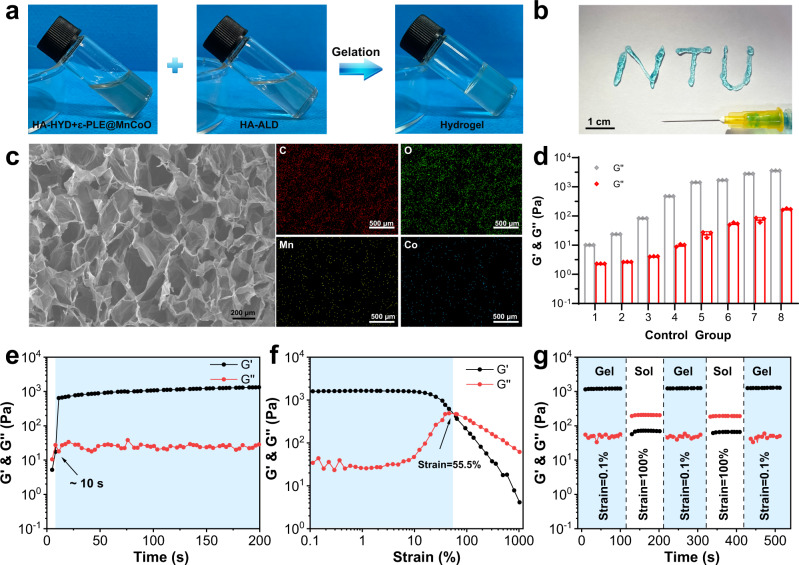
Characterization of the ε-PLE@MnCoO/Gel hydrogel. **a** Sol-gel transition of the ε-PLE@MnCoO/Gel hydrogel. **b** Injectability of the ε-PLE@MnCoO/Gel hydrogel. **c** SEM image of the lyophilized ε-PLE@MnCoO/Gel hydrogel and corresponding elemental mapping of the synthesized hydrogel. A representative image of three replicates from each group is shown. **d** G’ and G” values of hydrogels containing different concentrations of polymer. Data are presented as mean values ± SD (*n* = 3 independent samples). **e** Evolution of the G’ and G” values over time for the ε-PLE@MnCoO/Gel hydrogel. **f** Dependence of the G’ and G” values over strain for the ε-PLE@MnCoO/Gel hydrogel. **g** Step strain measurements of the ε-PLE@MnCoO/Gel hydrogel with a fixed frequency of 10 rad s^−1^. Each strain interval was kept as 100 s. Source data are provided as a Source Data file.

To regulate stem cell behavior in the RA microenvironment, the nanozyme-reinforced self-protecting hydrogel was rationally prepared as a delivery vehicle for enhancing prosthetic interface osseointegration in RA (Fig. 1). The overall gelation process of hydrogel was shown in Fig. 2a. In brief, the mixture solution of hydrazide modified hyaluronic acid (HA-HYD) and ε-PLE@MnCoO nanoparticles was slowly added into the solution of aldehyde modified hyaluronic acid (HA-ALD) under intense shaking, and a transparent light black hydrogel (ε-PLE@MnCoO/Gel) was gradually formed. The gelation mechanism of hydrogel was ascribed to dynamic imine bonds between -CHO groups of HA-ALD and -NH_2_ groups of ε-PLE@MnCoO nanoparticles, and dynamic acylhydrazone bonds between –CHO groups of HA-ALD and -CONH-NH_2_ groups of HA-HYD. The hydrogel at the early stage was weak due to the incomplete crosslinking of dynamic bonds, and this feature endowed hydrogel with a unique advantage of noninvasive injection. To verify the injectability, a 26-gauge (φ ≈ 260 µm) needle was used to extrude the mixed polymer solution. As displayed in Fig. 2b, ε-PLE@MnCoO/Gel could be pushed out of the needle, and the stable letters “NTU” were successfully written using the hydrogel. Such performance enabled hydrogel to be precisely injected around the target site by minimal invasive procedure^27^. The microstructure of ε-PLE@MnCoO/Gel was further investigated by SEM. The freeze-dried ε-PLE@MnCoO/Gel sample possessed a porous network structure (Fig. 2c), with porosity of nearly 96% (Supplementary Table 1). This structural feature allowed the ε-PLE@MnCoO/Gel with abundant-water content and a high swelling ratio under physiological conditions (Supplementary Fig. 13a), which would be beneficial to the cellular metabolism for improving the survival and proliferation of engineered cells. Meanwhile, energy-dispersive X-ray spectroscopy (EDS) mapping images illustrated the presence of C, O, Mn, and Co elements in the hydrogel, indicating the successful introduction of ε-PLE@MnCoO nanoparticles into the hydrogel.

The mechanical property of hydrogels has profound effects on the progression of cellular behaviors^28,29^. The mechanical properties of the ε-PLE@MnCoO/Gel hydrogel were comparatively investigated as a function of polymer concentration, and details of the hydrogel formula for Fig. 2d were provided in Supplementary Table 2. It was observed that the storage modulus (G’) and loss modulus (G”) of hydrogel elevated with the enhancement of polymer precursor solution, and G’ was dominant over G” throughout the whole concentration range. This result implied that the mechanical property of hydrogel could be adjusted in a large range by varying the concentration of polymer, and the enhancement of the polymer concentration facilitated the increased crosslinking density of the network. Subsequently, the hydrogel prepared from HA-HYD (2.5 wt%), ε-PLE@MnCoO (1 mg mL^−1^), and HA-ALD (5 wt%) was selected as the representative, and the rheological behavior of ε-PLE@MnCoO/Gel hydrogel at this formulation was then studied by means of monitoring G’ and G” in time sweep mode. As shown in Fig. 2e, the G’ and G” simultaneously increased over time, and G’ surpassed G” at about 10 s, suggesting the gradually cross-linked behavior of hydrogel. In addition, the representative strain amplitude sweep of hydrogel was analyzed. It was found that both the G’ and G” values decreased with the strain range from 0.1 to 1000%, exhibiting the typical viscoelastic behavior of hydrogels. In particular, the G’ and G” curves intersected at the strain of 55.5%, which was a crucial strain value for the breakdown of the hydrogel network (Fig. 2f). Owing to the dynamic nature of imine and acylhydrazone bonds, the ε-PLE@MnCoO/Gel hydrogel also exhibited extraordinary self-healing properties. Based on the strain amplitude sweep results, a continuous shear strain measurement was carried out by the alternating employment of a low strain and a high strain. According to Fig. 2g, both G’ and G” remained stable at the low strain (0.1%), while G’ and G” dramatically declined and G” surpassed G’ at the high strain (100%). However, when the high strain was removed, G’ and G” quickly recovered to the initial valves within 100 s in all cycles. These results indicated that the hydrogel network was destroyed at the high strain, and the breakdown network could be healed automatically at the low strain.

This self-healing property of hydrogels promised them a durable protection on engineered stem cells, leading to a high therapeutic efficacy. The biodegradability of hydrogels also plays a crucial role in bone tissue applications. To explore in vitro degradation ability of the hydrogel, a quantitative survey of the change in dry weight of hydrogels was further investigated under physiological conditions (i.e., in phosphate buffered saline at 37 °C). It was observed that the ε-PLE@MnCoO/Gel hydrogel could undergo more than 50% weight loss on the 18th day, and the degraded mass percentage was nearly 75% on the 24th day, demonstrating that the ε-PLE@MnCoO/Gel hydrogel gradually degraded as the incubation time increased (Supplementary Fig. 13b). The degradation of hydrogels was conducive to stem cell proliferation, migration, and remodeling of the synthetic matrix, finally providing space for the ingrowth of new bone. Taken together, these results indicated that the ε-PLE@MnCoO/Gel satisfied the requirements for cell culture and offered a substantial clinical advantage.

### H_2_O_2_-driven O_2_ production profile of nanozyme-reinforced hydrogel

**Fig. 3:**
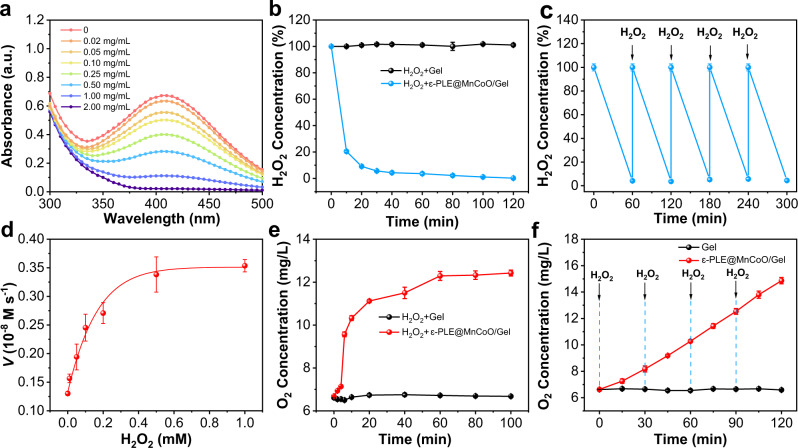
Catalase-like catalytic activity characterization of ε-PLE@MnCoO/Gel. **a** UV-vis absorption spectra of ε-PLE@MnCoO/Gel with the addition of Ti(SO_4_)_2_ solution. The concentration of ε-PLE@MnCoO nanozyme in hydrogel was varied, and the concentration of H_2_O_2_ was 1.0 mM. **b** Decomposition of H_2_O_2_ (1.0 mM) with and without ε-PLE@MnCoO nanozyme. **c** Repetitive catalytic H_2_O_2_ consumption-ability of ε-PLE@MnCoO/Gel with a repetitive addition of H_2_O_2_ (1.0 mM). **d** Catalytic kinetics of ε-PLE@MnCoO/Gel. The concentration of H_2_O_2_ was varied, and the initial reaction velocity (*V*) was monitored in PBS solution (pH 7.0) at 37 °C. **e** O_2_ generation ability with and without ε-PLE@MnCoO nanozyme. The concentration of H_2_O_2_ was 0.1 mM. **f** Continuous catalytic O_2_ generation ability of ε-PLE@MnCoO/Gel with a repetitive addition of H_2_O_2_ (0.1 mM). These data are presented as mean values ± SD (*n* = 3 independent experiments). Source data are provided as a Source Data file.

The substance exchange of metabolism process is identified as a determinant for regulating the physiological functions of living organisms^30^. Inspired by the metabolism process, the nanozyme-reinforced self-protecting hydrogel was designed to act as a H_2_O_2_-driven oxygenerator that could catalytically decompose H_2_O_2_ for O_2_ evolution. The catalase-like activity of ε-PLE@MnCoO/Gel was subsequently investigated by detecting the consumption of H_2_O_2_ using the titanium sulfate [Ti(SO_4_)_2_] colorimetric method. According to Fig. 3a, the H_2_O_2_ decomposition capacity of ε-PLE@MnCoO/Gel was proportional to the ε-PLE@MnCoO nanozyme content, demonstrating that the superior catalytic capabilities of the hydrogel were originated from the nanozyme. To better understand the H_2_O_2_ decomposition kinetics of the hydrogel, a time-dependent H_2_O_2_ assay was applied upon the presence of ε-PLE@MnCoO nanozyme (Fig. 3b). It was found that the addition of H_2_O_2_ (1.0 mM) to ε-PLE@MnCoO/Gel caused more than 70% of H_2_O_2_ decomposition for the first 10 min, and the complete depletion was achieved within 120 min. In contrast, no obvious loss of H_2_O_2_ content was observed when treated with the hydrogel in the absence of ε-PLE@MnCoO nanozyme, further confirming H_2_O_2_ decomposition was from the participation of the nanozyme. Importantly, ε-PLE@MnCoO/Gel could enduringly catalyze H_2_O_2_. Even after repeated addition of H_2_O_2_, the hydrogel still possessed excellent catalytic activity without an obvious delay (Fig. 3c). On basis of these results, the H_2_O_2_-driven O_2_ supply ability of the hydrogel was then studied. As shown in Fig. 3d, the Michaelis-Menten kinetic parameters of the catalase-mimicking catalytic process by ε-PLE@MnCoO/Gel were determined using H_2_O_2_ as a substrate. The key enzyme kinetic parameters of Michaelis-Menten constants (*K*_*m*_) and maximum reaction velocity (*V*_*max*_) values demonstrated the high catalytic activity of the hydrogel. Further studies were performed to evaluate the O_2_ production process of the hydrogel. The O_2_ concentration of the hydrogel solution notably increased from 6.7 to 11.1 mg L^−1^ after the addition of H_2_O_2_ in 20 min, while the primary hydrogel without the nanozyme exhibited no O_2_ generation (Fig. 3e). Moreover, ε-PLE@MnCoO/Gel was able to persistently produce O_2_ in the presence of H_2_O_2_, revealing its excellent catalytic durability (Fig. 3f).

### In vitro proliferation of BMSCs under protection of hydrogel

In healthy adults, a series of stem cell behavior mediates the bone repair process. Under RA conditions, however, the hypoxia and elevated ROS in synovium impede the survival and proliferation of stem cells^14,31^. To deal with these limitations, we inventively designed a ε-PLE@MnCoO/Gel hydrogel as a H_2_O_2_-driven oxygenerator to coculture BMSCs. Prior to in vivo application, the biocompatibility of the ε-PLE@MnCoO/Gel hydrogel (abbreviated as Gel-NPs) was investigated by live/dead staining and CCK-8 assay. For comparison, the pristine hydrogel without the addition of ε-PLE@MnCoO nanozyme was also prepared (abbreviated as Gel). The BMSCs exhibited good cell survival in the PBS, Gel, and Gel-NPs groups, and there was no apparent difference between all groups (Supplementary Figs. 14–16). Moreover, the BMSCs were distributed uniformly in the ε-PLE@MnCoO/Gel hydrogel matrix, and they maintained intact morphology without obvious damage (Supplementary Fig. 17). These results confirmed that the ε-PLE@MnCoO/Gel hydrogel had no obvious cytotoxicity to BMSCs.

**Fig. 4:**
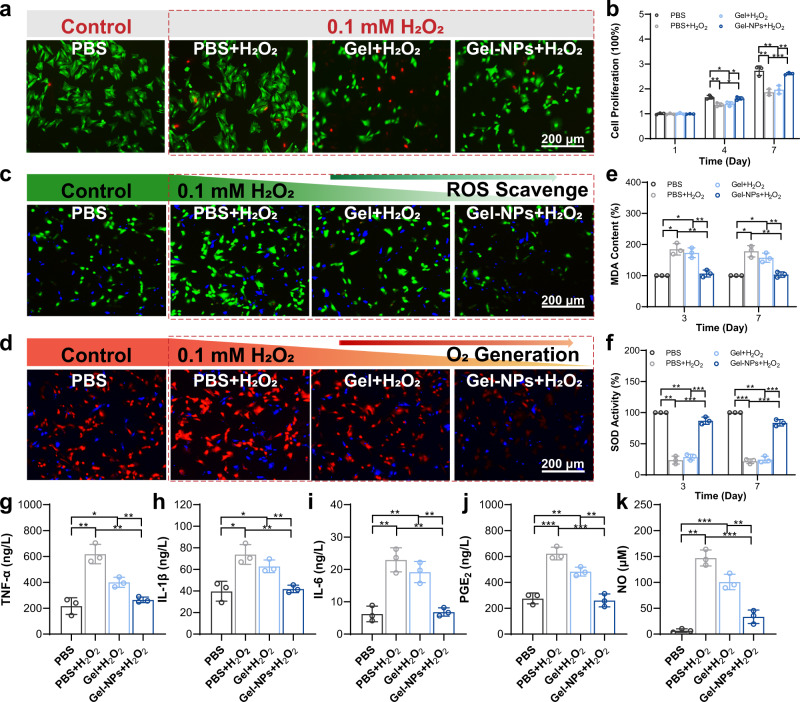
In vitro studies of BMSCs treated with the hydrogel. **a** Calcein-AM/propidium iodide (PI) staining of BMSCs after treatment with PBS, PBS + H_2_O_2_, Gel+H_2_O_2_, and Gel-NPs+H_2_O_2_. **b** Cell proliferation of BMSCs in different groups at the 1st, 4th, and 7th days. **c** ROS scavenge ability validated by a ROS probe (DCFH-DA) after different treatments. Green fluorescence from DCFH-DA indicates the presence of ROS. **d** Intracellular O_2_ generation assay monitored by an O_2_ probe [Ru(dpp)_3_Cl_2_]. Red fluorescence from Ru(dpp)_3_Cl_2_ is quenched by O_2_. **e** MDA activity of BMSCs after different treatments. **f** SOD activity of BMSCs after different treatments. **g**–**k** Expression of inflammatory mediators of BMSCs after different treatments including TNF-α (**g**), IL-1β (**h**), IL-6 (**i**), PGE_2_ (**j**), and NO (**k**). These data are presented as mean values ± SD (*n* = 3 independent experiments). Statistical significance was determined by two-tailed *t* test. *P < 0.05, **P < 0.01, and ***P < 0.001. Source data and exact *P* values are provided as a Source Data file.

Then, the protection of ε-PLE@MnCoO/Gel on the viability of stem cells under the RA pathological microenvironment was investigated. To imitate the H_2_O_2_-rich RA hostile microenvironment, BMSCs were separately incubated with PBS, Gel, and Gel-NPs and then treated with H_2_O_2_ (0.1 mM). Incubating with the PBS solution alone was used to simulate the normal physical condition. As shown in Fig. 4a, the co-incubation of BMSCs with the PBS + H_2_O_2_ and Gel+H_2_O_2_ groups displayed significant cell mortality under the damage of oxidative stress. It is worth noting that the Gel-NPs+H_2_O_2_ group exhibited only a minimal reduction in the cell viability, which was comparable with the normal condition (Supplementary Fig. 18). Additionally, the results of CCK-8 assay were consistent with the live/dead assay, revealing that BMSCs in the PBS + H_2_O_2_ and Gel+H_2_O_2_ treatment groups experienced apparently inhibited cell proliferation, whereas the Gel-NPs+H_2_O_2_ treatment group could alleviate the inhibitory effect of H_2_O_2_ on the cell proliferation (Fig. 4b).

### In vitro antioxidative and anti-inflammatory profile of hydrogel

The enhanced viability and proliferation of stem cells are mainly benefited from the dual ROS decomposition and O_2_ production profiles of the ε-PLE@MnCoO/Gel hydrogel. To visualize the in vitro ROS scavenge performance, BMSCs were separately seeded on the different treatment groups and then stained with a ROS indicator (2’,7’-dichlorodihydrofluorescein diacetate (DCFH-DA), Fig. 4c)^32^. Compared with the PBS + H_2_O_2_ and Gel+H_2_O_2_ groups, the green fluorescence intensity of BMSCs co-cultured with Gel-NPs+H_2_O_2_ group showed 2.70-fold and 2.13-fold decreases respectively, indicating that the ε-PLE@MnCoO/Gel hydrogel dramatically alleviated the intracellular oxidative stress (Supplementary Fig. 19a). Afterwards, in vitro O_2_ production capability was monitored by a typical O_2_ level indicator [Ru(dpp)_3_Cl_2_] (Fig. 4d)^33^. According to Supplementary Fig. 19b, the BMSCs cultured in Gel-NPs+H_2_O_2_ exhibited dramatically quenched red fluorescence intensity owing to the intracellular O_2_ elevation.

The ROS decomposition and O_2_ generation performance of hydrogels are also contributed to protecting stem cells from oxidative damage. Malonaldehyde (MDA) is a crucial end product of lipid peroxidation to reflect the cell oxidative damage of BMSCs^34^. As shown in Fig. 4e, the MDA content of BMSCs on the PBS + H_2_O_2_ and Gel+H_2_O_2_ groups significantly increased at 3 and 7 days compared with the PBS group, while the treatment with ε-PLE@MnCoO/Gel could obviously down-regulate the levels of MDA to show a comparable level of MDA content with the PBS group during the whole incubated period. Moreover, superoxide dismutase (SOD) is one of the most important antioxidant proteins in the human body, and the previous study evidenced that increased SOD activity can ascend the survival and proliferation of cells damaged by H_2_O_2_^35^. We next evaluated whether the ε-PLE@MnCoO/Gel hydrogel could activate the SOD activity of BMSCs under H_2_O_2_-rich RA pathological microenvironment. The SOD activity of BMSCs on the PBS + H_2_O_2_ and Gel+H_2_O_2_ groups obviously declined (Fig. 4f), since the H_2_O_2_ treatment tremendously inhibited the SOD activity. Significantly, these detrimental actions were evidently relieved by the antioxidative function of ε-PLE@MnCoO/Gel hydrogel. These studies highlighted that the developed ε-PLE@MnCoO/Gel hydrogel could downgrade the adverse effects of H_2_O_2_ on the survival and proliferation of BMSCs by maintaining the intrinsic SOD activity and inhibiting intracellular MDA production, which would be potentially favorable for the restriction of inflammation.

The continuous oxidative damage of RA can aggravate the occurrence of excessive inflammation. A cascade of inflammatory reactions plays a critical role in the pathological microenvironment of RA, and the proinflammatory cytokines including tumor necrosis factor α (TNF-α), interleukin-1β (IL-1β), interleukin-6 (IL-6), and prostaglandins (PGE_2_) would abundantly express in the rheumatoid joint cavity^36,37^. On account of the antioxidant ability, the ε-PLE@MnCoO/Gel hydrogel could also exhibit obvious anti-inflammatory capability. As confirmed by enzyme-linked immunosorbent assay (ELISA) in Fig. 4g–k, the continuous oxidative stress induced by H_2_O_2_ treatment (0.1 mM) could remarkably increase the expression of TNF-α, IL-1β, IL-6, PGE_2_, and nitric oxide (NO) in BMSCs, whereas ε-PLE@MnCoO/Gel could depress the expression of these cytokines even under the H_2_O_2_-rich condition. Collectively, the ε-PLE@MnCoO/Gel hydrogel inhibited inflammatory responses through H_2_O_2_-induced continuous oxidative stress, meeting the requirements as the suitable biomaterial carrier for the transplantation of BMSCs.

### In vitro osteogenic differentiation promotion by hydrogel

**Fig. 5:**
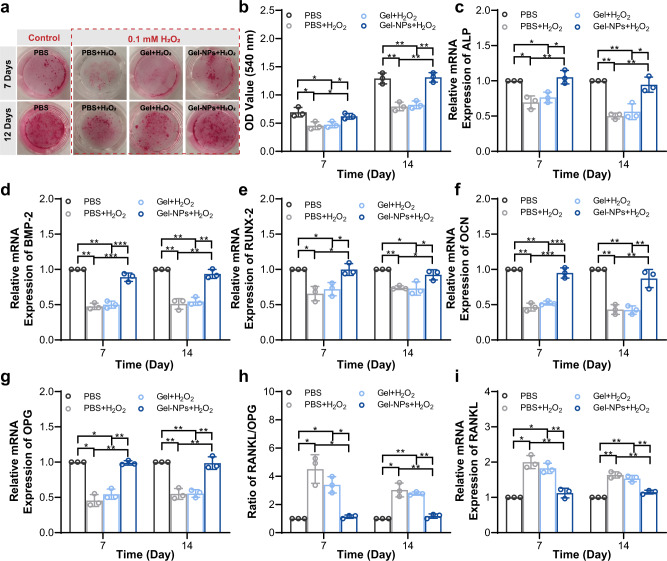
In vitro osteogenic differentiation promoted by hydrogel. **a** Gross observation of mineralized nodules stained by Alizarin Red. **b** Semi-quantitative analysis of mineralized nodules in different groups. **c**–**f** Relative mRNA expression levels of osteogenic genes from BMSCs after different treatments, including ALP (**c**), BMP-2 (**d**), RUNX-2 (**e**), and OCN (**f**). **g**–**i** Relative mRNA expression levels of osteoclastic genes from BMSCs after different treatments, including OPG (**g**), RANKL (**h**), and RANKL/OPG (**i**). These data are presented as mean values ± SD (*n* = 3 independent experiments). Statistical significance was determined by two-tailed *t* test. *P < 0.05, **P < 0.01, and ***P < 0.001. Source data and exact *P* values are provided as a Source Data file.

It is well known that continuous oxidative stress can inhibit the osteogenic differentiation of mesenchymal stem cells, thus inducing osteoblast apoptosis and reducing bone mineral density in vivo^38^. The dual ROS decomposition and O_2_ production functions of ε-PLE@MnCoO/Gel are attributed to the osteogenic differentiation of BMSCs. The formation of calcium nodule deposition is an important marker for the osteogenic differentiation of BMSCs. The mineralized nodules were first stained with Alizarin Red to reveal the osteogenic differentiation of BMSCs. Gross observation (Fig. 5a) and semi-quantitative analysis (Fig. 5b) suggested that the mineralization of BMSCs in the PBS + H_2_O_2_ and Gel+H_2_O_2_ groups was prominently restrained, while BMSCs treated with PBS alone or Gel-NPs+H_2_O_2_ had obvious calcium deposition after 7 and 14 days of differentiation. It was concluded that the ε-PLE@MnCoO/Gel hydrogel could dramatically reverse the adverse impact of H_2_O_2_ on osteogenic differentiation.

Furthermore, the genes involved in osteogenic differentiation of BMSCs were determined by real-time quantitative polymerase chain reaction (RT-qPCR). The relative mRNA of osteogenic gene markers including alkaline phosphatase (ALP), bone morphogenetic protein-2 (BMP-2), runt-related transcription factor 2 (RUNX-2), and osteocalcin (OCN) were selected as representative examples^39,40^. According to Fig. 5c–f, the mRNA expression of multiple osteogenic differentiation markers on the PBS + H_2_O_2_ and Gel+H_2_O_2_ groups suffered from the prominent oxidative damage, while they could be activated under the protection of the ε-PLE@MnCoO/Gel hydrogel. Apart from the capability of enhancing osteogenic differentiation, the ε-PLE@MnCoO/Gel hydrogel could inhibit the osteoclastogenesis of BMSCs in the pathological oxidative microenvironment, which was represented by up-regulating the osteoclastogenesis inhibition indicator of osteoprotegerin (OPG), as well as decelerating the receptor activator of nuclear factor-κ B ligand (RANKL) and RANKL/OPG (Fig. 5g–i). Together, these data confirmed that the nanozyme-reinforced hydrogel could augment osteogenic activities of stem cells, capable of being as the desirable stem cell delivery vehicle for improving prosthetic interface osseointegration in RA.

### In vivo immunomodulation of hydrogel-mediated stem cell therapy

Encouraged by the satisfactory biocompatibility, improved proliferation, and superior osteogenic differentiation in vitro, the hydrogel-mediated stem cell therapy against RA was then assessed in vivo. To more convincingly prove the advantages, we conducted the certification in a severe RA rabbit model induced by subcutaneously injecting ovalbumin (OVA) and Freund’s adjuvant, and a cylindrical bone defect matching the scaffold was prepared on the distal femur tissues to mimic the joint replacement surgery of RA. Clinically, the 3D printed microporous titanium alloy scaffolds, one of the most popular inorganic orthopedic implants, can provide mechanical support to the lesion tissue and facilitate the reconstruction of large-scale joint defects of RA^41,42^. We thus administered the cell-laden nanozyme-reinforced hydrogel into the large micropores of 3D printed titanium alloy prosthesis, defined as the pTi@Gel-NPs group. To further investigate the influence of different factors, another two control groups were constructed, including the primary 3D printed microporous titanium alloy scaffold loaded with BMSCs (pTi group) and pTi modified with Gel-containing BMSCs (pTi@Gel group). Once the rabbits developed typical symptoms of RA, such as the elevated joint surface temperature and swollen joint, in situ implantations of pTi, pTi@Gel, and pTi@Gel-NPs scaffolds were performed, respectively. According to Supplementary Fig. 20, the distinct reduction of the skin temperature and joint diameter from the RA rabbit implanted with pTi@Gel-NPs scaffold were observed during the whole therapeutic period, demonstrating the pTi@Gel-NPs scaffold could relieve the local excessive inflammation and synovial hyperplasia of the joint.

**Fig. 6:**
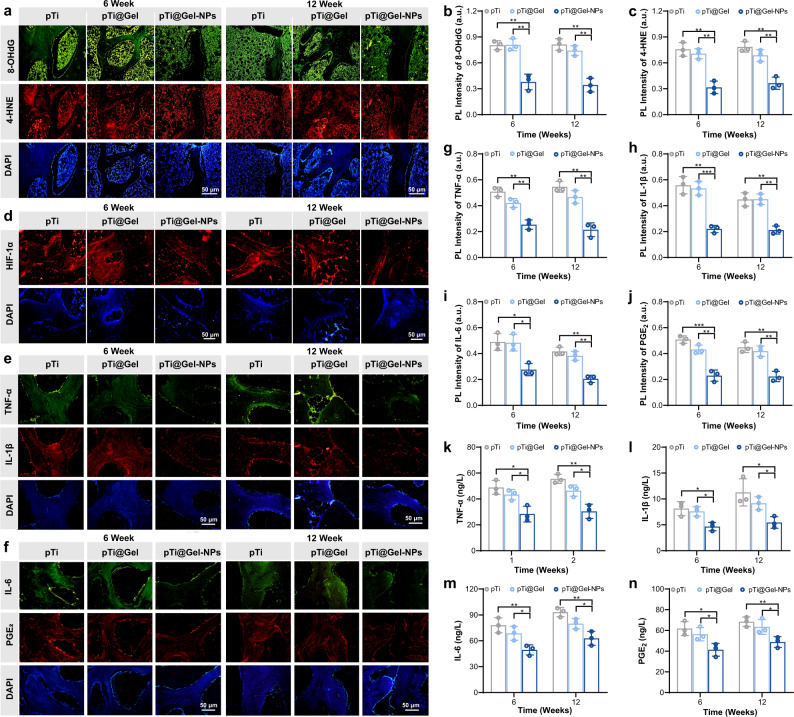
In vivo immunomodulation of hydrogel for stem cell therapy. **a**–**c** Representative immunofluorescence staining images (**a**) and quantitative statistics of 8-OHdG (**b**) and 4-HNE (**c**) content at weeks 6 and 12 after the treatment with pTi, pTi@Gel, and pTi@Gel-NPs scaffolds. **d** Representative immunofluorescence staining images of HIF-1α on the bone tissues around the scaffolds at weeks 6 and 12 after different treatments. **e**–**j** Representative immunofluorescence staining images (**e**, **f**) and quantitative statistics of TNF-α (**g**), IL-1β (**h**), IL-6 (**i**), and PGE_2_ (**j**) on the bone tissue around the scaffolds at weeks 6 and 12 after different treatments. **k**–**n** Content of inflammatory cytokines in synovial fluid, including TNF-α (**k**), IL-1β (**l**), IL-6 (**m**), and PGE_2_ (**n**). These data are presented as mean values ± SD (*n* = 3 independent experiments). Statistical significance was determined by two-tailed *t* test. *P < 0.05, **P < 0.01, and ***P < 0.001. Source data and exact *P* values are provided as a Source Data file.

To determine whether nanozyme-reinforced hydrogel was associated with the antioxidative and hypoxia-attenuating capacities of the stem cell therapy, we performed a series of examinations on the synovium, joint fluid, and bone tissue of the left knee joint. Immunofluorescence staining of bone tissues showed remarkable DNA damage in the pTi and pTi@Gel groups, as evidenced by the high levels of oxidative DNA damage marker 8-hydroxydeoxyguanosine (8-OHdG) and lipid peroxidation marker 4-hydroxy-2-nonenal (4-HNE). In contrast, the treatment of pTi@Gel-NPs exhibited lower 8-OHdG and 4-HNE expression (Fig. 6a–c). Moreover, the ROS level in RA bone tissue represents the oxidative stress status during inflammation. We then evaluated the ROS variations through the ROS probe (DCFH-DA). As revealed by the images of immunofluorescence staining, the RA bone tissue treated with the pTi@Gel-NPs group displayed notably lower green fluorescence than that after pTi or pTi@Gel treatment during the whole treatment cycle, demonstrating that the nanozyme-reinforced hydrogel could effectively reduce the level of ROS and attenuate oxidative stress in RA (Supplementary Fig. 21). On the other hand, hypoxia-inducible factors 1α (HIF-1α), as a marker of tissue hypoxia, is highly expressed in the hypoxic environment of RA joints. The hypoxia-attenuating ability of nanozyme-reinforced hydrogel was then verified by immunofluorescence staining of HIF-1α. According to Fig. 6d and Supplementary Fig. 22, the downregulation in HIF-1α expression was most prominent after treatment with the pTi@Gel-NPs group, implicating its simultaneous inhibitory of HIF-1α signaling pathways and synergistic production of O_2_. Taken together, these results demonstrated the superior antioxidation and hypoxic reliever properties of the nanozyme-reinforced hydrogel in vivo, showing the potential to serve as an advanced stem cell delivery vehicle in the management of hostile microenvironment.

It is known that continued oxidative stress and HIF-1α expression can lead to a cascade of inflammatory reactions. Considering the excellent antioxidative and down-regulated HIF-1α abilities, the high anti-inflammatory effect of pTi@Gel-NPs could be predicted. To evaluate the inflammatory response in vivo, we applied hematoxylin and eosin (H&E) to stain synovial tissue sections harvested in weeks 6 and 12 (Supplementary Fig. 23). In the pTi and pTi@Gel groups, robust infiltration of inflammatory cells was clearly observed in synovial tissues, while there was substantially milder infiltration in the pTi@Gel-NPs group. The expression of inflammatory cytokines in bone tissue around the scaffolds was also determined by immunofluorescence staining (Fig. 6e, f), and the quantitative analysis of fluorescence intensity indicated that the levels of TNF-α, IL-1β, IL-6, and PGE_2_ were certainly suppressed in the pTi@Gel-NPs group (Fig. 6g–j). Moreover, the expression levels of these immunological cytokines in the synovial fluid also exhibited a similar tendency (Fig. 6k–n). The expression of inflammatory factors of TNF-α, IL-1β, IL-6, and PGE_2_ at the serum level was subsequently detected to investigate how pTi@Gel-NPs influenced the systemic immune response. It was observed that there was no significant downregulation of inflammatory factors in serum in all groups (Supplementary Fig. 24). A well-established hallmark of RA is the pathological inflammation associated with a large number of molecules, and the inhibition of one or a few molecules at local inflammatory levels may be insufficient to halt or reverse disease progression on the systemic level. Thus, the nanozyme-reinforced hydrogel encapsulated with BMSCs could accumulate at arthritic sites and prominently alleviate the local symptoms of RA. These equally effective local antioxidants and anti-inflammatory functions have a broad impact on bone regeneration and osseointegration.

### Prosthetic interface osseointegration by hydrogel-mediated stem cell therapy in vivo

**Fig. 7:**
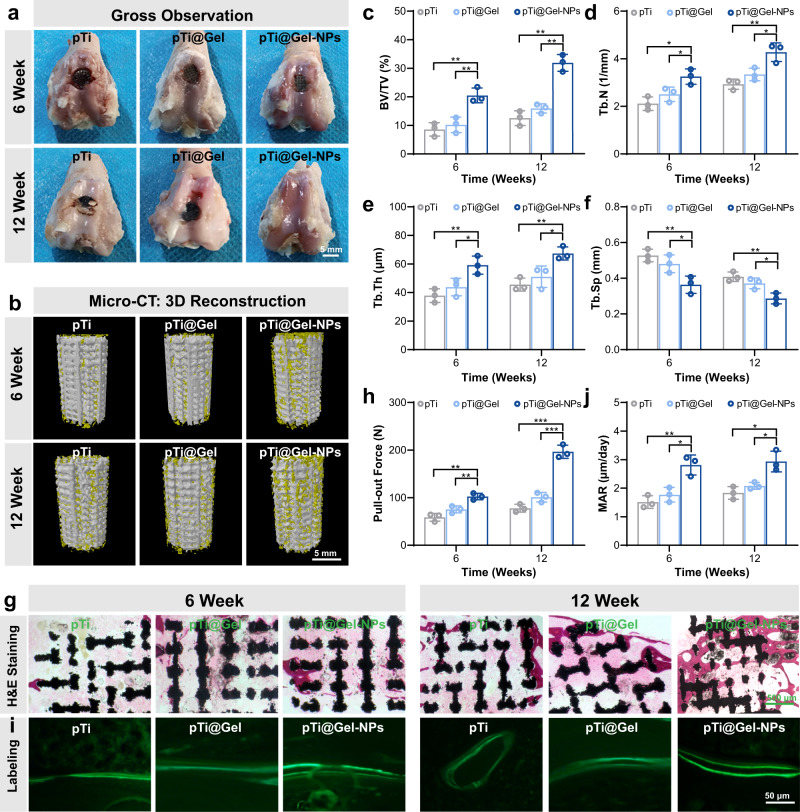
Prosthetic interface osseointegration by hydrogel-mediated stem cell therapy. **a** Gross appearance for the articular surface of distal femurs at weeks 6 and 12 after the treatment of pTi, pTi@Gel, and pTi@Gel-NPs scaffolds. **b** Representative 3D reconstruction images of bone regeneration from different scaffold treatments. **c**–**f** Quantitative statistics of BV/TV (**c**), Tb.N (**d**), Tb.Th (**e**), and Tb.Sp (**f**) from different groups according to Micro-CT scanning. **g** H&E staining of bone defects at weeks 6 and 12 after the treatment. **h** Quantitative analysis of the osseointegration according to biomechanical pull-out test. **i** Calcein fluorescence double-labeling staining of bone defects at weeks 6 and 12 after the treatment. **j** Quantitative analysis of mineral apposition rate via calcein fluorescence double-labeling. These data are presented as mean values ± SD (*n* = 3 independent experiments). Statistical significance was determined by two-tailed *t* test. *P < 0.05, **P < 0.01, and ***P < 0.001. Source data and exact *P* values are provided as a Source Data file.

The intra-articular infiltration of inflammatory cells has a negative effect on the downstream activities and biological behavior of normal bone cells such as osteogenic differentiation and biomineralization, eventually leading to insufficient osteogenic activity. In view of the alteration of inflammatory response, the in vivo osteogenic bioactivity of BMSCs on the nanozyme-reinforced hydrogel was investigated. At weeks 6 and 12 after the operation, femur tissues were collected for subsequent analysis. According to Fig. 7a, the knee joints of the pTi and pTi@Gel groups possessed severe RA features, such as cartilage damage and bone erosion. In sharp contrast, the treatment by the pTi@Gel-NPs scaffold exhibited maximal regenerated cartilage and superior articular morphology. Good osseointegration between prosthesis and host bone is a major requirement for joint replacement to guarantee daily load-bearing applications^43–45^. The bone integration ability was then evaluated by micro-computed tomography (Micro-CT), and typical 3D reconstruction images were shown in Fig. 7b. Consistently, the pTi@Gel-NPs treated group expressed noticeable new bone tissue on the bone-implant interface as compared with the other treatment groups. Quantitative morphometric analysis on the parameters of bone volume/tissue volume ratio (BV/TV), trabecular number (Tb.N), trabecular thickness (Tb.Th), and trabecular separation (Tb.Sp) was conducted to evaluate the regenerated bone ingrowth into the microporous scaffolds at both weeks 6 and 12 after the implantation. It was found that the level of BV/TV ratio on the pTi@Gel-NPs group was apparently higher than that of pTi and pTi@Gel groups, which was 20.47 ± 2.58% vs 10.19 ± 2.71% and 8.59 ± 2.35% at week 6, and 31.85 ± 2.95% vs 15.90 ± 1.61% and 12.50 ± 2.58% at week 12, respectively (Fig. 7c). Moreover, the pTi@Gel-NPs group showed the maximum increased Tb.N (from 3.25 ± 0.32 to 4.27 ± 0.39 mm^−1^, Fig. 7d) and Tb.Th (from 59.16 ± 6.38 to 67.32 ± 4.60 μm, Fig. 7e), and the most significantly decreased Tb. Sp from 0.36 ± 0.05 to 0.29 ± 0.03 mm (Fig. 7f). These results revealed that pTi@Gel-NPs scaffold had a great bone protection effect, and BMSCs encapsulated in nanozyme-reinforced hydrogel could enhance osseointegration of Ti implant in vivo for facilitating bone reconstruction in RA treatment. We also compared the expression of osteogenesis-related markers and observed that the significantly up-regulated expression of ALP, BMP-2, RUNX-2, and OCN occurred in the pTi@Gel-NPs treated group. In contrast, pTi and pTi@Gel treatment revealed no notably therapeutic effect (Supplementary Fig. 25). Based on these results, it could be concluded that the nanozyme-reinforced hydrogel improved the harsh microenvironment of RA through its pleiotropic roles including anti-inflammation and antioxidation so that the transplanted BMSCs could fully exert high therapeutic efficacy in localized bone defects.

The osteointegration of scaffolds with the surrounding bone tissue was further analyzed by H&E staining (Fig. 7g). In the pTi and pTi@Gel groups, obvious gaps could be observed between the host bone and scaffolds on account of the limited bone regeneration. Excitingly, the pronounced bone formation was displayed after the treatment with pTi@Gel-NPs, especially at week 12, demonstrating efficiently improved osteogenic differentiation of BMSCs and accelerated bone integration. The osseointegration strength between the scaffolds and the host bone was then measured by the biomechanical push-out test. As shown in Fig. 7h, the pull-out peak force values of the pTi, pTi@Gel, and pTi@Gel-NPs groups were 58.83 ± 8.15, 75.32 ± 7.35, and 102.39 ± 6.87 N at week 6, and 77.74 ± 8.27, 101.02 ± 10.07, and 196.51 ± 13.78 N at week 12, respectively. Meanwhile, the dynamic histomorphometric analysis results according to calcein double-labeling indicated that the pTi@Gel-NPs group quickened the bone regeneration around the scaffold, as demonstrated by the obviously increased mineral apposition rate (MAR, Fig. 7i, j). Finally, the biosafety of the transplanted hydrogels was evaluated by H&E staining observation of major organs including the heart, liver, spleen, lung, and kidney, and there was no obvious organ damage or inflammation in the experimental groups (Supplementary Fig. 26). These results suggested the good biocompatibility of scaffolds in vivo, which promised their therapeutic efficacy and long-term biosafety for future clinical applications.

## Discussion

RA is a refractory inflammatory disorder characterized by progressive and irreversible impairment of joint functions. Albeit the stem cell-based therapies have been envisioned as a powerful approach for RA intervention, the therapeutic efficacy of stem cells remains highly compromised due to the overproduction of ROS and the poor oxygen supply of RA pathological microenvironment. To address the above-mentioned challenges, we presented here a biological metabolism-inspired hydrogel made from the dynamically cross-linked natural polymer with the introduction of MOF-derived nanozyme for reshaping the hostile RA microenvironment. The engineered hydrogel exhibited superior biocompatibility, injectable performance, and remarkable self-healing ability. Significantly, the nanozyme-reinforced hydrogel could synergistically decompose ROS and generate O_2_, which enabled it to protect the implanted BMSCs from ROS and hypoxia-mediated death. In addition to the cell delivery vehicle, the nanozyme-reinforced self-protecting hydrogel could be employed as a H_2_O_2_-driven oxygenerator to facilitate stem cell survival, proliferation, and osteogenic differentiation in vitro (Figs. 4 and 5).

Based on the satisfactory results of cellular experiments, we further investigated the therapeutic potential of hydrogel-mediated stem cell therapy for immunomodulation in RA rabbit model. Immunofluorescence staining of bone tissues demonstrated obvious DNA damage in the pTi and pTi@Gel control groups, while the efficient antioxidative ability of the pTi@Gel-NPs group was observed (Fig. 6a–c). The infiltration of inflammatory cells, especially M1 macrophages, has been recognized as the most prominent cells associated with initiating RA and promoting disease progression. To reveal the anti-inflammatory ability of hydrogel-mediated stem cell therapy, various types of proinflammatory cytokines secreted by M1 macrophages, including TNF-α, IL-1β, IL-6, and PGE_2_, were monitored by immunohistological staining and ELISA kit analysis in both bone tissue and synovial fluid. Consistent with the cell experiment results, the treatment by pTi@Gel-NPs displayed a great anti-inflammatory performance in reducing the TNF-α, IL-1β, IL-6, and PGE_2_ levels as compared with other groups (Fig. 6k–n).

In RA, the infiltration of inflammatory cells is associated with eroded articular cartilage and destroyed subchondral bone tissue. Especially in the knee joint, the impaired joint function may lead to limited mobility, and RA patients ultimately have to undergo joint replacement surgery to restore joint function and relieve pain. To avoid a series of postoperative complications, improvement of bone regeneration and osseointegration is in great demand for joint replacement in RA patients. The neutralization of inflammatory factors is able to elicit a potent therapeutic effect, thus locally depressing overall bone erosion progression and improving osteogenic activities. Considering the conspicuous and synergetic immunomodulation of hydrogel-mediated stem cell therapy, we proposed that pTi@Gel-NPs can enhance prosthetic interface osseointegration in a RA rabbit model. Intriguingly, the pTi and pTi@Gel groups partially attenuated the arthritis symptom of cartilage damage and bone erosion, whereas the pTi@Gel-NPs group exhibited the maximal regenerated cartilage and superior articular morphology (Fig. 7a). In good agreement with the gross observation, the 3D reconstructed Micro-CT images revealed that the pTi@Gel-NPs treatment displayed noticeable new bone tissues on the bone-implant interface (Fig. 7b–f). Moreover, the H&E staining and dynamic histomorphometric analyses indicated that the pTi@Gel-NPs group strengthened the osseointegration (Fig. 7g) and quickened the new bone formation around the scaffolds (Fig. 7i, j). Overall, these results confirmed that pTi@Gel-NPs achieved superior bone regeneration efficacy and satisfactory osteointegration owing to the hydrogel-mediated stem cell therapy. Thereby, the cell-laden nanozyme-reinforced hydrogel is a powerful system to reduce the loosening, displacement, and periprosthetic fractures after prosthesis implantation in RA. Our work represents a promising approach for the mediation of stem cell therapy, which offers a vast application prospect for the intervention of other immune-related diseases even beyond RA.

## Methods

Supplementary experimental details are shown in the Supplementary Information.

### Preparation of ε-PLE@MnCoO

The ε-PLE@MnCoO nanoparticles were synthesized according to the following procedures. (1) Fabricated process of Mn_3_[Co(CN)_6_]_2_ nanoparticles: Mn_3_[Co(CN)_6_]_2_ was synthesized by following the well-established method^25^. Briefly, Mn(CH_3_COO)_2_·4H_2_O (0.15 mmol) and poly(vinylpyrrolidone) (0.6 g) were added in ethanol (30 mL) and distilled water (10 mL) to form precursor A. Similarly, K_3_[Co(CN)_6_] (0.08 mmol) was solubilized in distilled water (20 mL) to form precursor B. Then, precursor B was dropwise added to precursor A under intensive stirring. White turbidity was generated immediately, and the above mixture was allowed to rest at room temperature for 24 h. The obtained Mn_3_[Co(CN)_6_]_2_ nanoparticles were purified by several consecutive washing/centrifugation cycles with C_2_H_5_OH and H_2_O and dried at room temperature for further modification. (2) Fabricated process of Mn_3_[Co(CN)_6_]_2_@SiO_2_ nanoparticles: Mn_3_[Co(CN)_6_]_2_ (36 mg) was dispersed in C_2_H_5_OH (36 mL). Then, NH_3_·H_2_O (2.16 mL, 28% in H_2_O) in C_2_H_5_OH (36 mL) and tetraethyl orthosilicate (136 µL) was sequentially added to the above solution under vigorous stirring. The reaction solution was allowed to proceed for 2 h and then maintained at room temperature overnight. The obtained product was purified by several consecutive washing/centrifugation cycles with C_2_H_5_OH and H_2_O, and dried for further modification. (3) Fabricated process of MnCoO nanoparticles: The as-prepared Mn_3_[Co(CN)_6_]_2_@SiO_2_ nanoparticles were placed directly into a muffle furnace at 500 °C for 4 h, and then etched with 4 M NaOH solution. The MnCoO nanoparticles were purified several times by centrifugation for further use. (4) Preparation of ε-PLE@MnCoO nanoparticles: ε-PLE@MnCoO nanoparticles were facilely obtained by adding ε-polylysine solution (50 mL, 10 mg mL^−1^) into aqueous suspension of MnCoO nanoparticles (10 mL, 1 mg mL^−1^) under sonication for 30 min. The ε-PLE@MnCoO nanoparticles were collected by centrifugation, and washed with water three times to remove the residual ε-polylysine. The obtained ε-PLE@MnCoO nanoparticles were dried via lyophilization and then stored at room temperature for further use.

### Preparation of hydrogels

(1) Synthesis of hydrazide-modified hyaluronic acid (HA-HYD): As illustrated in Supplementary Fig. 27, HA (1.0 g, molecular weight = 90,000–100,000 Da) was solubilized in distilled water (100 mL), and activated by 1-ethyl-3-(3-dimethylaminopropyl)carbodiimide (2.0 g). Subsequently, adipic acid dihydrazide (9.0 g) was added to the above solution under pH = 4.5. The reaction was allowed to proceed under intense stirring for 24 h, followed by dialysis (molecular weight cut off 3500) against distilled water for 3 days. Then, the obtained HA-HYD polymer was dried via lyophilization, and then preserved at 4 °C for the next application^46^. (2) Synthesis of aldehyde-modified hyaluronic acid (HA-ALD): As illustrated in Supplementary Fig. 28, a precursor solution was formed by dissolving HA (1.0 g, molecular weight = 1,000,000–2,000,000 Da) and NaIO_4_ (1.0 g) in deionized water (100 mL). Then, the precursor solution was intensely stirred in dark for 3 h, followed by adding ethylene glycol (10 mL) to terminate the reaction. After another 1 h, the prepared polymer was purified by dialysis (molecular weight cut off 3500) against distilled water for 3 days. Then, the obtained HA-ALD polymer was dried via lyophilization, and then preserved at 4 °C for the next application^47^. (3) Synthesis of ε-PLE@MnCoO/Gel: Precursor A was formed by dissolving ε-PLE@MnCoO (1 mg mL^−1^, 100 µL) and HA-HYD (2.5 wt%, 500 µL) together in PBS solution (pH = 7.4). HA-ALD with different concentrations (0.078, 0.156, 0.313, 0.625, 1.25, 2.5, 5, and 10 wt%) was dissolved in PBS solution (pH = 7.4) to form precursor B. For the preparation of the ε-PLE@MnCoO/Gel hydrogel, precursor A was mixed with precursor B under intense shaking.

### Porosity of hydrogels

The porosity of ε-PLE@MnCoO/Gel hydrogel was evaluated based on the ethanol displacement method. Briefly, the completely gelled ε-PLE@MnCoO/Gel hydrogel was prepared and weighed (*W*_*1*_) before immersing in absolute ethanol. After reaching saturation in ethanol, the hydrogel was collected and weighed (*W*_*2*_). Thereafter, the porosity of hydrogel was calculated via the following equation: Porosity = (*W*_*1*_ – *W*_*2*_) / *ρV* × 100%, where *ρ* is the density of absolute ethanol (*ρ* = 0.789 g cm^−3^) and *V* is the volume of the hydrogel.

### Swelling ratio of hydrogels

The swelling ratio of ε-PLE@MnCoO/Gel hydrogel was examined by recording the change in wet weight. First, the completely gelled ε-PLE@MnCoO/Gel hydrogel (500 µL) was immersed in PBS solution (5 mL) at 37 °C. At predetermined time points (0, 5, 10, 15, 20, 30, 40, and 60 min), the hydrogel was withdrawn from the solution, and excess water was wiped off using filter paper. Afterwards, the ε-PLE@MnCoO/Gel hydrogel was weighed, and the swelling ratio was appraised using the following equation: Swelling ratio = (*W*_*t*_ – *W*_*0*_)/*W*_*0*_ × 100%, where *W*_*t*_ represents the weight of hydrogel after incubation in PBS solution, and *W*_*0*_ means the initial weight of hydrogel.

### In vitro degradation ability of hydrogels

In vitro degradation performance of ε-PLE@MnCoO/Gel hydrogel was measured by recording the change in dry weight. In detail, the completely gelled ε-PLE@MnCoO/Gel hydrogel was lyophilized, and then the dried hydrogel (50 mg) was incubated in PBS solution at 37 °C. To reduce bacterial growth, sodium azide (0.02 wt%) was added to the above solution. At predetermined time points (0, 3, 6, 9, 12, 15, 18, 21, 24, 27, and 30 days), the residual hydrogel was collected and washed with deionized water three times. Then, the hydrogel was lyophilized, and the weight remaining was calculated according to the following equation: Weight remaining = 1–(*W*_*0*_ – *W*_*d*_)/*W*_*0*_ ×  100%, where *W*_*0*_ is the initial dry weight of hydrogel, and *W*_*d*_ represents the weight of the hydrogel after degradation at different time points.

### H_2_O_2_ scavenging and O_2_ generation of hydrogel

The H_2_O_2_ depletion ability of the hydrogel was monitored by mixing H_2_O_2_ (1.0 mM, 1 mL) and ε-PLE@MnCoO/Gel hydrogel (1 mL) in PBS solution at 37 °C. Then, Ti(SO_4_)_2_ precursor solution was prepared by dissolving 24 % Ti(SO_4_)_2_ (2.66 mL) and H_2_SO_4_ (16.66 mL) together in distilled water (100 mL). After designated times, the supernatant of hydrogel (100 µL) was collected and added with Ti(SO_4_)_2_ solution (200 µL), and the concentration of H_2_O_2_ could be determined by the absorbance at 405 nm^48^. At predetermined time intervals, the above supernatant (100 µL) was collected, and the O_2_ concentration was monitored by an oxygen probe.

### Cell viability

To explore the biocompatibility of the prepared hydrogel, BMSCs (at a density of 50,000 cells/well) were cultured in plates, and then treated with the hydrogel (200 μL) or PBS (200 μL). At predetermined time points (1st, 4th, and 7th day), a CCK-8 assay kit was applied to quantify cell proliferation. On the 3rd day, the cell viability was also evaluated by a Calcein-AM/PI kit according to the producer’s instructions. In order to simulate the oxidative environment of RA, BMSCs were seeded in different groups with the addition of H_2_O_2_ (10 μL, 100 μM). At predetermined time points, the cell proliferation and viability were detected using the CCK-8 assay kit and Calcein-AM/PI staining.

### Evaluation of intracellular ROS (H_2_O_2_) depletion and O_2_ generation

The intracellular H_2_O_2_ scavenge capacity of ε-PLE@MnCoO/Gel hydrogel was verified by a H_2_O_2_ probe DCFH-DA. In brief, BMSCs (at a density of 5.0 × 10^4^ cells/well) were seeded in plates, and then incubated with ε-PLE@MnCoO/Gel or Gel under the oxidative environment (H_2_O_2_ with a final concentration of 100 µM). For comparison, BMSCs (at a density of 5.0 × 10^4^ cells/well) were also incubated with PBS with or without H_2_O_2_ (with a final concentration of 100 µM). After 3 d incubation, DCFH-DA (10 μM in FBS-free Dulbecco’s modified eagle medium (DMEM)) was added to all experimental groups, and the intracellular ROS level of all experimental groups was observed by a confocal microscope. The intracellular O_2_ generation capacity of ε-PLE@MnCoO/Gel hydrogel was demonstrated by an O_2_ probe Ru(dpp)_3_Cl_2_. Briefly, Ru(dpp)_3_Cl_2_ (10 μg mL^−1^) was added to the different groups. After incubation at 37 °C for 3 d, the O_2_ level of all treatment groups was observed by a confocal microscope^32^. SOD activity and MDA formation were also applied to indicate the antioxidative abilities of the hydrogel. After being cultured under a pathological oxidative microenvironment for 3 and 7 days, the cells were collected, where the SOD activity and MDA formation were appraised by commercial SOD and MDA assay kits, respectively.

### Intracellular inflammatory cytokine detection

BMSCs (at a density of 5.0 × 10^4^ cells/well) were seeded in plates, and then incubated with ε-PLE@MnCoO/Gel or Gel under the oxidative environment (H_2_O_2_ with a final concentration of 100 µM). For comparison, BMSCs were incubated with PBS with or without H_2_O_2_. After 3 d incubation, the content of inflammatory cytokine (TNF-α, IL-1β, IL-6, and PGE_2_) in the cell culture supernatants was analyzed by commercially available ELISA kits. The level of NO released from BMSCs was detected by the Griess reagent. After incubation for 10 min, the absorbance of the product was monitored by a microplate reader at 540 nm and analyzed according to the standard curve.

### Cell differentiation

Alizarin red S staining was applied to evaluate the osteogenic differentiation of BMSCs. BMSCs (at a density of 5.0 × 10^4^ cells/well) were seeded in plates and then incubated with ε-PLE@MnCoO/Gel or Gel under the oxidative environment (H_2_O_2_ with a final concentration of 100 µM). For comparison, BMSCs were also incubated with PBS in the presence or absence of H_2_O_2_. After the incubation at 37 °C for 24 h, the previous medium was changed for the fresh osteogenic induction differentiation medium including low-glucose DMEM along with β-glycerol-phosphate (10 mM), ascorbate-2-phosphate (50 μM), and dexamethasone (0.1 μM). After designated times, the cells were treated with paraformaldehyde and then stained with 0.1% ARS at pH 4.1–4.3. After gross observation, the semi-quantitative analysis of cell mineralization was conducted by adding 2% cetylpyridinium chloride to dissolve the calcium nodules, and quantified by a microplate reader at 540 nm. Furthermore, TRIzol Reagent was applied to extract the total RNA of all samples, and the osteogenic and osteoclastic differentiation-related genes were assessed by real-time qPCR. The primer sequences were displayed in Supplementary Table 3, and glyceraldehyde 3-phosphate dehydrogenase was selected as the housekeeping gene.

### Preparation of 3D printed porous Ti_6_Al_4_V scaffold

The 3D printed porous Ti_6_Al_4_V scaffold was produced layer-by-layer according to the previous report^49^. The printing parameters were set to 800 μm in pore size, 70% in porosity, and 300 μm in strut size to obtain the cylindrical scaffold (φ 6 mm × L 10 mm) for osseointegration evaluation in RA animal model.

### RA animal model establishment and scaffold implantation

All animal procedures were conducted according to the guidelines for Care and Use of Laboratory Animal Experience of Jilin University and approved by the Animal Ethics Committee of Jilin University. Thirty-six male New Zealand white rabbits (2.5–3.0 kg, 5-month-old) were randomly allocated into three groups for in vivo study, and the RA rabbit models were established according to the previously established methods^9^. Briefly, the mixed solution of OVA (20 g L^−1^) and Freund’s adjuvant (1 mL) was weekly subcutaneously injected into the dorsal region. On the 4th week, the mixed solution (0.5 mL) was injected into the left knee joint cavity of rabbits to make the RA models.

Two weeks later, the RA rabbits were first anesthetized by the injection of pentobarbital, and then the implantation surgery was performed. Briefly, the lateral longitudinal incision of the distal femur on the left hind limb was selected, and bone defects matched with cylindrical scaffolds were prepared using a bone drill. Subsequently, three groups of as-prepared scaffolds (pTi, pTi@Gel, pTi@Gel-NPs) loaded with BMSCs (at a density of 2 × 10^6^ cells/well) were transplanted into the bone defects, respectively. After suturing the injured tissues, the RA rabbits were fed in their individual cages and injected intramuscularly with penicillin to avoid postoperative infection. On the 14th and 4th day before sacrifice, all RA rabbits were intramuscularly injected with calcein (8 mg kg^−1^) to assess the mineral apposition rate. At weeks 6 and 12 after the operation, the rabbits were sacrificed, and the femur samples were collected for the subsequent analysis.

### Bone regeneration assessment

The bone regeneration of samples was scanned by Micro-computed tomography. In particular, the cylindrical bone defects containing the scaffolds were chosen as the region of interest for reconstruction, and the parameters of bone histomorphometry were then quantitatively analyzed. Additionally, biomechanical experiments of the push-out test were carried out to investigate osteointegration. After fixing the bone samples on the console, the indenter propelled parallel along the long axis of the scaffolds was set at a speed of 0.1 mm/s, and the peak push-out force was recorded when the scaffolds were removed from the bone.

### Histological observation

All dehydrated samples were embedded in methyl methacrylate. After being fixed with a sample holder, undecalcified bone sections were sliced. The sliced calcein fluorescence was observed by a confocal microscope to analyze the mineral apposition rate. To analyze osseointegration between regenerated bone tissue and micropore of the scaffolds, the sections were treated with H&E staining. After the metallic scaffolds were pushed out through biomechanical experiments, the bone samples around the scaffolds were decalcified and embedded, and then the sections were prepared for immunofluorescence staining.

### Statistical analysis

Quantitative data were presented as mean ± standard deviation, and conducted from at least triple independent experiments. Error bars represent the standard deviation of measurements within each experiment (*p < 0.05, **p < 0.01, ***p < 0.001). One-way ANOVA was applied for comparisons across multiple groups followed by Tukey’s post hoc test using SPSS 19.0 (SPSS Inc., Chicago, USA).

### Reporting summary

Further information on research design is available in the Nature Portfolio Reporting Summary linked to this article.

## Supplementary information

Supplementary Information

Peer Review File

Reporting Summary

## Data Availability

All data are available within the Article and Supplementary Files, or available from the corresponding authors upon reasonable request. Source data are available for Figs. 2–7 and Supplementary Figs. 2, 3, 5, 6, 8–13, 15, 16, 18–22, 24, and 25 in the associated Source Data file. Source data are provided with this paper.

## Source data

Source Data
