# Supplementary material for: Nanozyme-reinforced hydrogel as a H2O2-driven oxygenerator for enhancing prosthetic interface osseointegration in rheumatoid arthritis therapy
Source: Nat Commun. 2022 Nov 9;13:6758. doi: 10.1038/s41467-022-34481-5 (PMC9646710; doi:10.1038/s41467-022-34481-5)
Supplement: Supplementary file 1 — Supplementary Information [file 41467_2022_34481_MOESM1_ESM.pdf]

Supplementary Information

Nanozyme-Reinforced Hydrogel as a H_2O_2-Driven Oxygenerator for Enhancing Prosthetic Interface Osseointegration in Rheumatoid Arthritis Therapy

Zhao et al.

Supplementary experimental details

Materials: Hyaluronic acid (HA, Mw = 90-100 KDa, Mw = 1,000-2,000 KDa) was obtained from Yuanye Biological Technology Co., Ltd (Shanghai, China). Adipic acid dihydrazide (ADH, ~98%), 2',7'-dichlorodihydrofluorescein diacetate (DCFH-DA), 1-ethyl-3-(3-dimethylaminopropyl)carbodiimide (EDC), Freund's adjuvant, ovalbumin (OVA, ~99 %), sodium periodate ($NaIO_4$, ~99%), and tris(4,7-diphenyl-1,10-phenanthroline) ruthenium(II) dichloride [Ru(dpp)$_3$Cl$_2$] were purchased from Sigma-Aldrich (St. Louis, MO, USA). Ammonium hydroxide solution ($NH_3 \cdot H_2O$, 28% in H_2O, ~99%), manganese acetate [Mn(CH3COO)2·4H2O], potassium cobaltocyanide (K3[Co(CN)6]), and tetraethyl orthosilicate (TEOS, ~99%) were from Aladdin. Sodium hydroxide (NaOH) was purchased from Shanghai Macklin Biochemical Co., Ltd (Shanghai, China). Hydrogen peroxide (H_2O_2) 30% aqueous solution, poly(vinylpyrrolidone) (K-30), and $Ti(SO_4)_2$ were from Sinopharm Group Co. Ltd (Beijing, China). Ethanol (C_2H_5OH, ~99.7 %) was obtained from Beijing Chemical Works (Beijing, China). Spherical medical-grade titanium alloy (Ti_6Al_4V) powder (Grade 23, particle size 45-100 μm) was obtained from AK Medical Co., Ltd. (Beijing, China). Fetal bovine serum (FBS), low-glucose Dulbecco's Modified Eagle's Medium (DMEM), and 0.25% trypsin/EDTA were provided by Gibco® Life Technologies (Grand Island, NY, USA). Calcein-AM/propidium iodide (PI), cell counting kit-8 (CCK-8), commercial malonaldehyde (MDA), Griess reagent, and superoxide dismutase (SOD) assay kits were purchased from Beyotime Biotechnology (Shanghai, China). IL-1β, IL-6, PGE_2 and TNF-α ELISA kits were purchased from Shanghai Haling Biological Technology Co., Ltd. (Shanghai, China). Alizarin red, 4% paraformaldehyde, phosphate-buffered saline (PBS), and Triton X-100 were supplied by Solarbio (Beijing, China). TRIzol reagent was provided by Invitrogen Life Technology (Carlsbad, CA, USA). Prime script RT reagent kit and SYBR premix Ex Taq II kit were purchased from Takara Bio (Dalian, China). Primary antibodies of mouse monoclonal 4-HNE, rabbit monoclonal TNF-α, and rabbit polyclonal PGE_2 were purchased from were purchased from Abcam (Cambridge, UK). Primary antibodies of rabbit polyclonal IL-1β and rabbit polyclonal HIF-1α were purchased from Affinity Biosciences LTD (Jiangsu, China). Primary antibody of rabbit polyclonal 8-OHdG was supplied by Bioss Antibodies (Beijing, China). Primary antibody of rabbit polyclonal IL-6 was supplied by Cell Signaling Technology (Massachusetts, USA). Primary antibody of mouse monoclonal 4-HNE, at 1:150 dilution: Anti-4 Hydroxynonenal antibody [HNEJ-2], Mouse monoclonal [HNEJ-2] to 4

Hydroxynonenal, Cat. No. ab48506. Primary antibody of rabbit polyclonal 8-OHdG, at 1:100 dilution: Rabbit polyclonal, Cat. No. bs-1278R. Primary antibody of rabbit polyclonal IL-1β, at 1:150 dilution: Rabbit polyclonal, Cat. No. AF5103. Primary antibody of rabbit monoclonal TNF-α, at 1:100 dilution: Recombinant Anti-TNF alpha antibody [EPR19147], Rabbit monoclonal [EPR19147] to TNF alpha, Cat. No. ab183218. Primary antibody of rabbit polyclonal PGE2, , at 1:100 dilution: SureLight® APC Anti-PGE2 receptor EP4 subtype antibody, SureLight® APC Rabbit polyclonal to PGE2 receptor EP4 subtype, Cat. No. ab92763. Primary antibody of rabbit polyclonal IL-6, at 1:150 dilution: IL-6 (D5W4V) XP® Rabbit mAb (Mouse Specific) Cat. No. #12912. Primary antibody of rabbit polyclonal HIF-1α, , at 1:200 dilution: Rabbit polyclonal, Cat. No. AF1009. Hematoxylin and eosin (H&E) staining kit was provided by Thermo Fisher Scientific (Shanghai, China). Rabbit BMSCs osteogenic induction differentiation medium (RBXMX-90021) was supplied by Cyagen Biosciences (Suzhou, China). All chemicals were used as received without further purifications.

Characterizations: Scanning electron microscopy (SEM) images were measured on a microscopy (Thermo scientific, Thermo APREO S) under an acceleration voltage of 3 kV. Rheological measurements were performed with a rheometer (ThermoFisher Scientific, HAAKE MARS 40) at room temperature. Powder X-ray diffraction (XRD) images were obtained from a diffractometer (German Bruker, D8ADVANCE). X-ray photoelectron spectroscopy (XPS) was performed with a spectrometer (Thermo Fisher Scientific, K-Alpha+) equipped with Mg Kα excitation (1253.6 eV). Fourier transform infrared spectroscopy (FT-IR) spectra were measured on a spectrophotometer (Shimadzu, IR Affinity-1) collected at wavenumbers ranging from 4000 to 500 cm^{-1}. UV-Vis absorption spectra were obtained from a spectrometer (Shimadzu, UV-3600PLUS) equipped with a quartz cell of 1 cm path length at room temperature. Dynamic laser scattering (DLS) measurements were carried out using a Zetasizer UV spectrometer (Beckman, DelsaMax CORE). Photoluminescence data were obtained from a spectrofluorimeter (Hitachi, F7000) equipped with a quartz cell of 1 cm path length at room temperature. The O_2 generation capacity was monitored by an oxygen probe (JPBJ-608 portable dissolved oxygen meter, Shanghai REX Instrument Factory). The articular skin temperature was recorded using an electronic thermometer (Yuwell THP79JU, Shanghai, China). The joint diameter of RA rabbits was monitored using a micrometer caliper (Links, Harbin, China). The push-out test was carried out by a dynamic testing machine (Shimadzu, Nakagyo-ku Kyoto, Japan). 3D printed porous Ti_6Al_4V scaffolds

were manufactured layer-by-layer by an EBM system (Q10, Arcam, Sweden). The osteogenic and osteoclastic differentiation-related genes were analyzed by a CFX96TM Real-time qPCR system (Bio-Rad, Hercules, CA, USA). The parameters of bone histomorphometry were quantitatively analyzed by a μCT auxiliary software (NRecon, version 1.6.6). The percentage of live cells was analyzed by Image J software (NIH, Bethesda, MD, USA). The optical density (OD) was measured at 450 nm by a microplate reader (Multiskan EX, Thermo Fisher Scientific Inc., China). The stained samples were imaged with the confocal microscope (Olympus Fluoview FV1000, USA). The undecalcified bone sections were prepared by a diamond saw microtome (Leica 2500E, Leica SpA, Milan, Italy). Statistical calculations were performed using OriginPro 2019 (64 bit) and graphPad Prism (V.8.0.2).

Supplementary Table 1. Porosity of ε-PLE@MnCoO/Gel hydrogel using HA-HYD (2.5 wt%), ε-PLE@MnCoO (1 mg mL^{-1}), and HA-ALD (5 wt%).

Group	Porosity = $(W_1 - W_2) / \rho V \times 100\%$
1	W_1 = 0.473 g, W_2 = 0.110 g, V = 0.5 cm^{-3}<br>Porosity = 92.01%
2	W_1 = 0.499 g, W_2 = 0.113 g, V = 0.5 cm^{-3}<br>Porosity = 97.85%
3	W_1 = 0.500 g, W_2 = 0.110 g, V = 0.5 cm^{-3}<br>Porosity = 98.86%

Supplementary Table 2. Details of the hydrogel formula.

Hydrogel	Concentration of different polymers
1	500 µL of 2.5 w% HA-HYD<br>100 µL of ε-PLE@MnCoO (1 mg mL^{-1})<br>500 µL of 0.078125 wt% HA-ALD
2	500 µL of 2.5 w% HA-HYD<br>100 µL of ε-PLE@MnCoO (1 mg mL^{-1})<br>500 µL of 0.15625 wt% HA-ALD
3	500 µL of 2.5 w% HA-HYD<br>100 µL of ε-PLE@MnCoO (1 mg mL^{-1})<br>500 µL of 0.3125 wt% HA-ALD
4	500 µL of 2.5 w% HA-HYD<br>100 µL of ε-PLE@MnCoO (1 mg mL^{-1})<br>500 µL of 0.625 wt% HA-ALD
5	500 µL of 2.5 w% HA-HYD<br>100 µL of ε-PLE@MnCoO (1 mg mL^{-1})<br>500 µL of 1.25 wt% HA-ALD
6	500 µL of 2.5 w% HA-HYD<br>100 µL of ε-PLE@MnCoO (1 mg mL^{-1})<br>500 µL of 2.5 wt% HA-ALD
7	500 µL of 2.5 w% HA-HYD<br>100 µL of ε-PLE@MnCoO (1 mg mL^{-1})<br>500 µL of 5 wt% HA-ALD
8	500 µL of 2.5 w% HA-HYD<br>100 µL of ε-PLE@MnCoO (1 mg mL^{-1})<br>500 µL of 10 wt% HA-ALD

Supplementary Table 3. Primer sequences of genes.

Gene subtype	Oligonucleotide primers (5'-3')
ALP	F: 5'-TCCCACTTTGTCTGGAACCG-3'<br>R: 5'-ACTGGGCCTGGTAGTTGTTG-3'
RUNX-2	F: 5'-GGCGCATTTCAGGTGCTTC-3'<br>R: 5'-GTGGTGGCAGGTAGGTATGG-3'
OCN	F: 5'-AGAGTCTGGCAGAGGCTCA-3'<br>R: 5'-CAGGGGATCCGGGTAAGGA-3'
BMP-2	F: 5'-CAGCCAACTCGAAATTCCCC-3'<br>R: 5'- CAAGTGGGTCACTTCCACCA-3'
OPG	F: 5'-CACCTACCTGAAGCAGCACT-3'<br>R: 5'-CCCTTCCTCGCATTCACACA-3'
RANKL	F: 5'-CCCACGAGTATTCACTGCCA-3'<br>R: 5'-TGACACACACTCCGCTTGAG-3'
GAPDH	F: 5'-AGACACGATGGTGAAGGTCG-3'<br>R: 5'-TGCCGTGGGTGGAATCATAC-3'

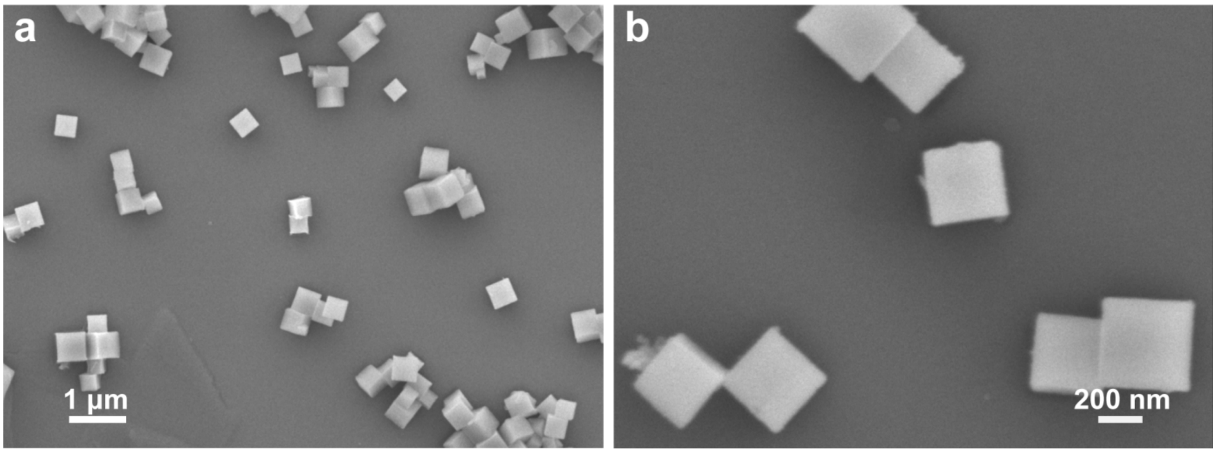

Supplementary Figure 1. **Representative SEM images.** (a,b) SEM images of $Mn_3[Co(CN)_6]_2$ MOF nanoparticles at different magnification. A representative image of three replicates from each group is shown.

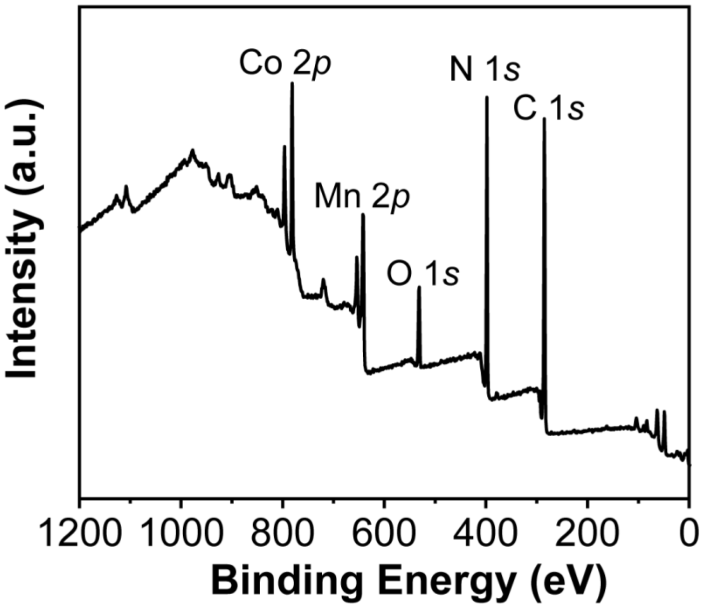

Supplementary Figure 2. XPS spectrum of $Mn_3[Co(CN)_6]_2$ MOF nanoparticles. Source data are provided as a Source Data file.

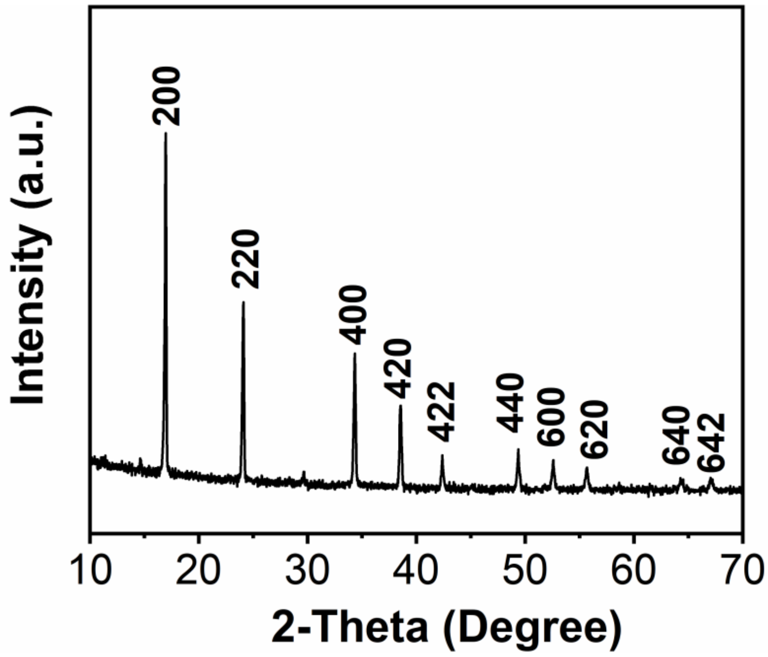

Supplementary Figure 3. Powder XRD pattern of $Mn_3[Co(CN)_6]_2$ MOF nanoparticles. Source data are provided as a Source Data file.

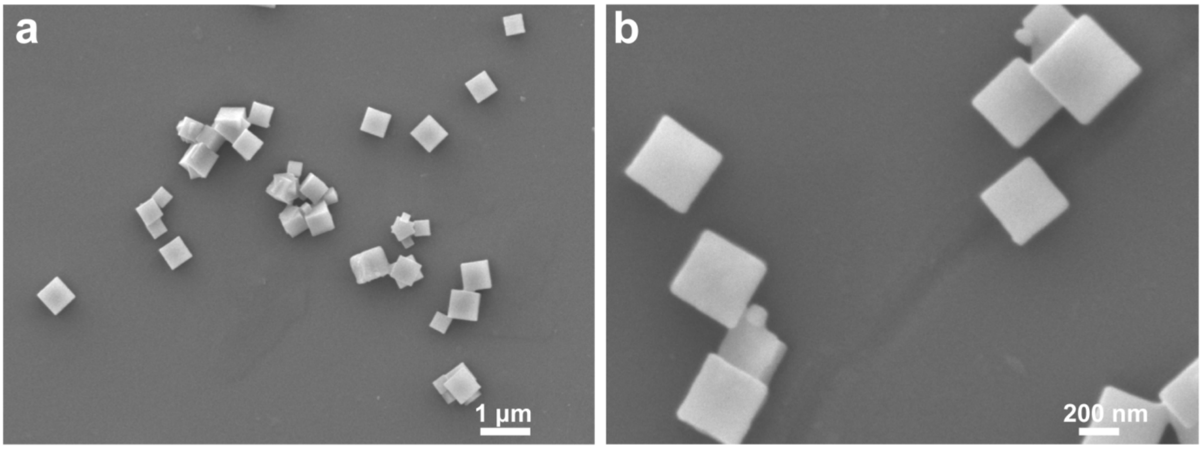

Supplementary Figure 4. **Representative SEM images**. (a,b) SEM images of $Mn_3[Co(CN)_6]_2@SiO_2$ nanoparticles at different magnification. A representative image of three replicates from each group is shown.

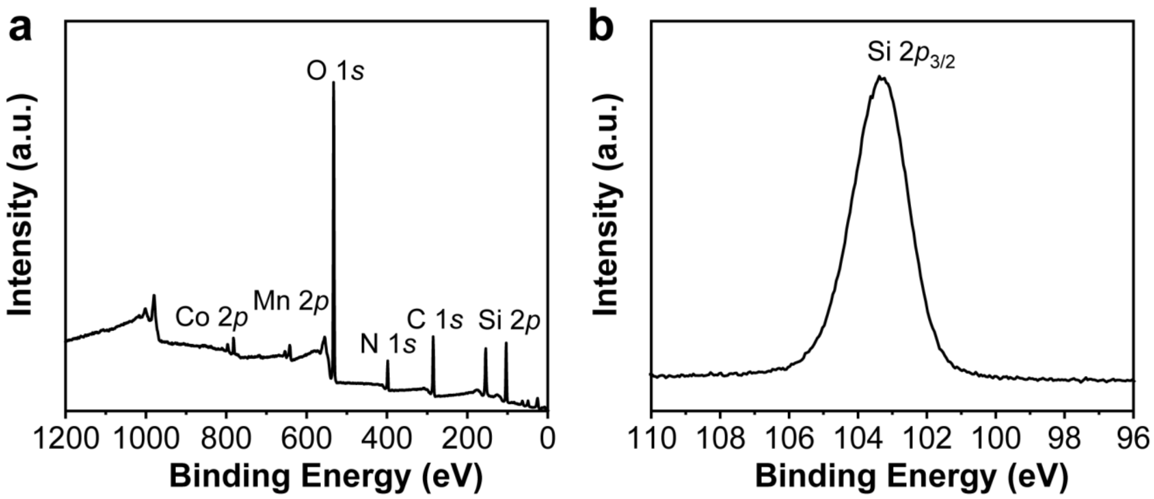

Supplementary Figure 5. **XPS spectra.** (a) XPS spectrum of $Mn_3[Co(CN)_6]_2@SiO_2$ nanoparticles. (b) High-resolution XPS spectrum of Si 2p in $Mn_3[Co(CN)_6]_2@SiO_2$ nanoparticles. Source data are provided as a Source Data file.

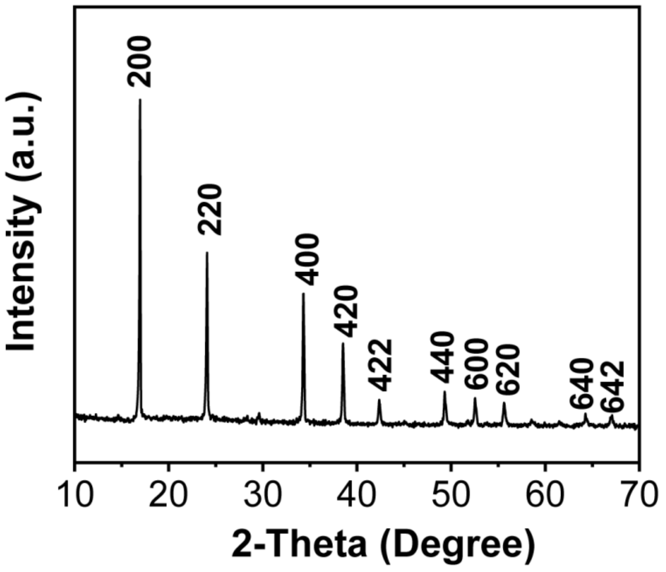

Supplementary Figure 6. Powder XRD pattern of $Mn_3[Co(CN)_6]_2@SiO_2$ nanoparticles. Source data are provided as a Source Data file.

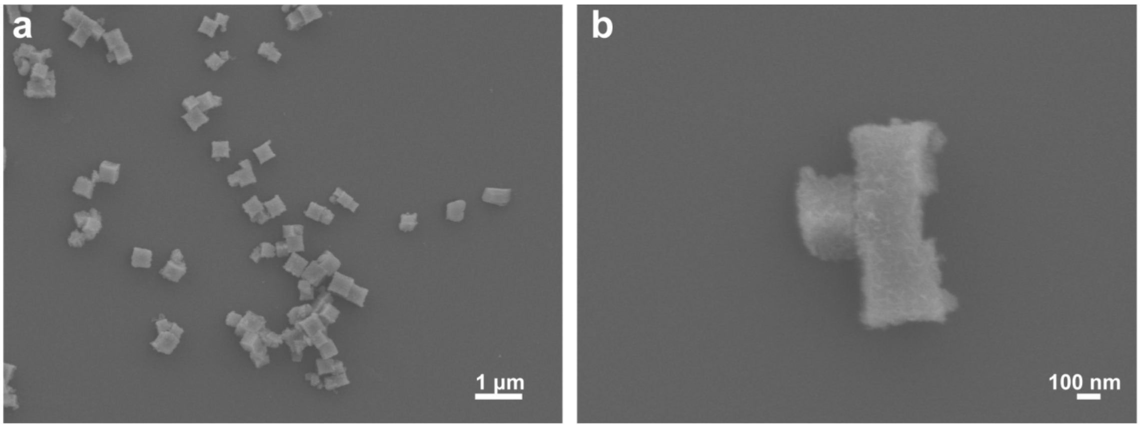

Supplementary Figure 7. **Representative SEM images.** (a,b) SEM images of MnCoO nanoparticles at different magnification. A representative image of three replicates from each group is shown.

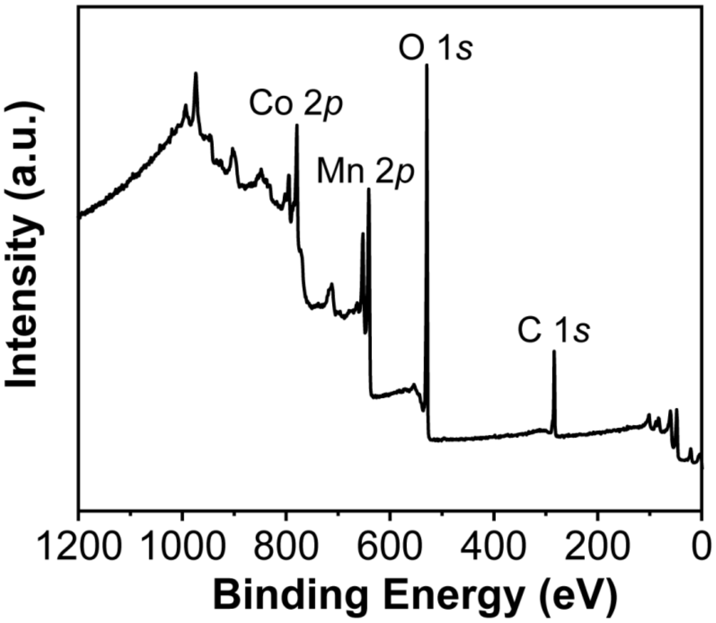

Supplementary Figure 8. XPS spectrum of MnCoO nanoparticles. Source data are provided as a Source Data file.

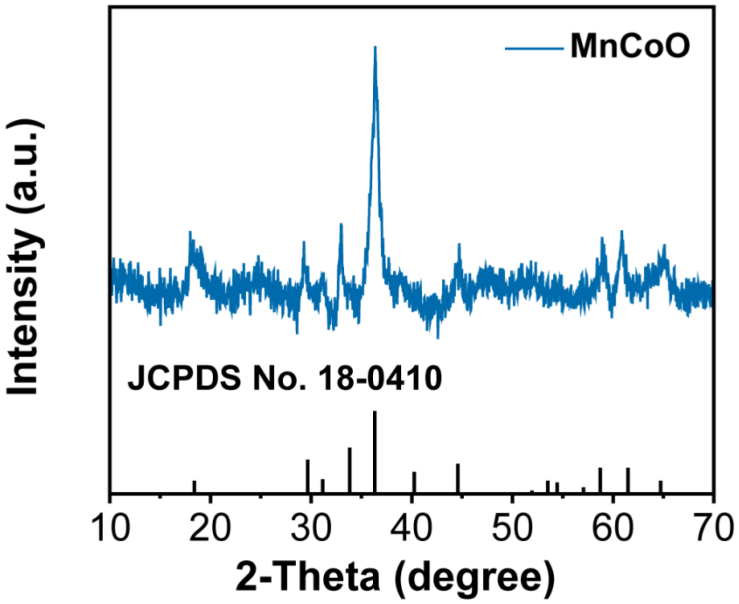

Supplementary Figure 9. Powder XRD pattern of MnCoO nanoparticles. Source data are provided as a Source Data file.

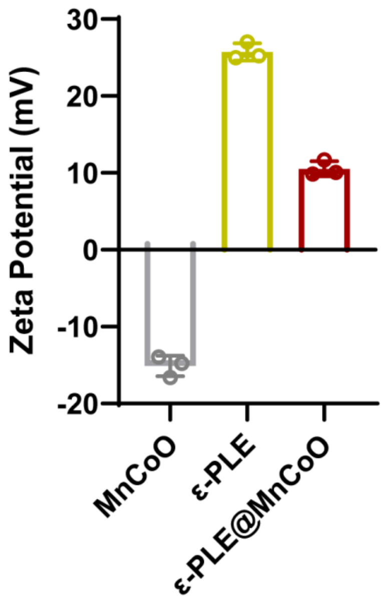

Supplementary Figure 10. Zeta potential of MnCoO nanoparticles, ε-PLE, and ε-PLE@MnCoO nanoparticles. Data are presented as mean values ± SD (n = 3 independent experiments). Source data and exact *P* values are provided as a Source Data file.

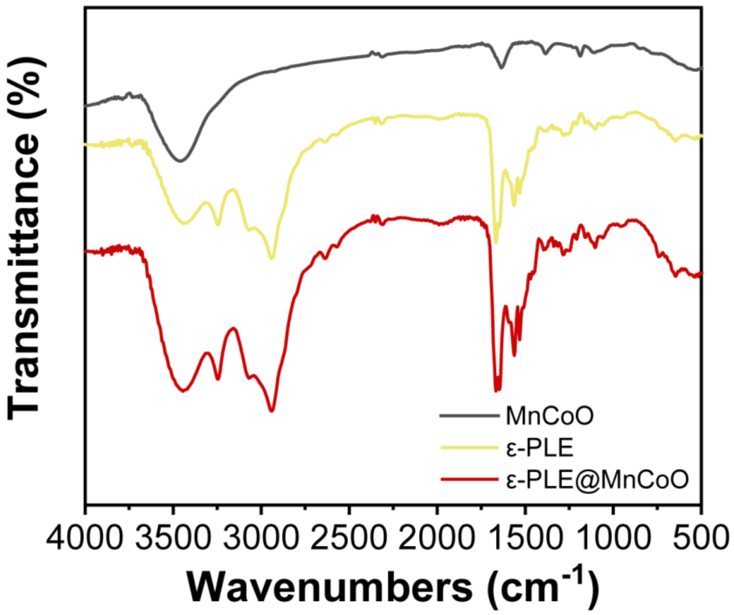

Supplementary Figure 11. FT-IR spectra of MnCoO nanoparticles, ε-PLE, and ε-PLE@MnCoO nanoparticles. Source data are provided as a Source Data file.

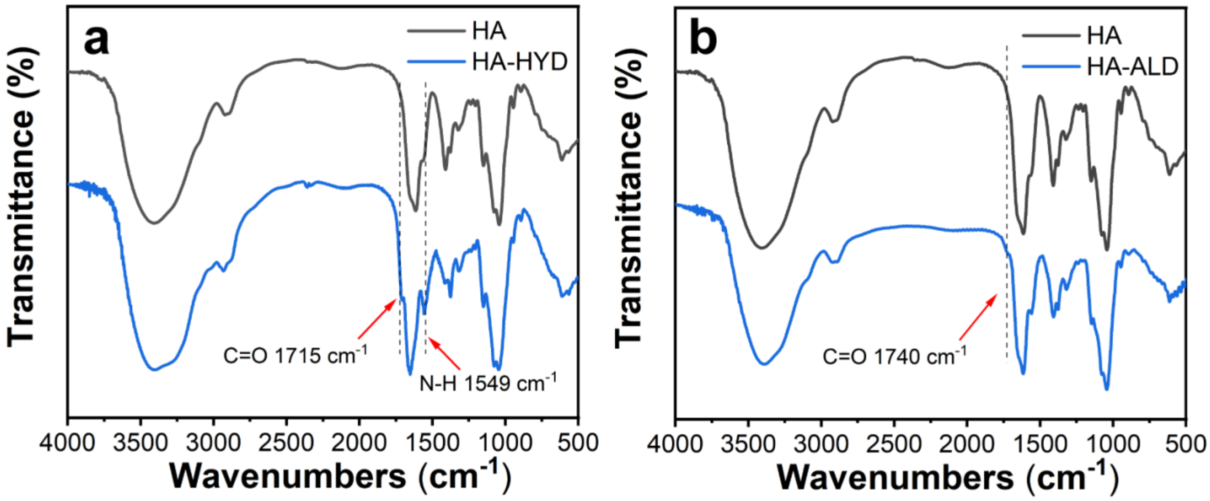

Supplementary Figure 12. **FT-IR spectra.** (a) FT-IR spectra of HA and HA-HYD. (b) FT-IR spectra of HA and HA-HYD. Source data are provided as a Source Data file.

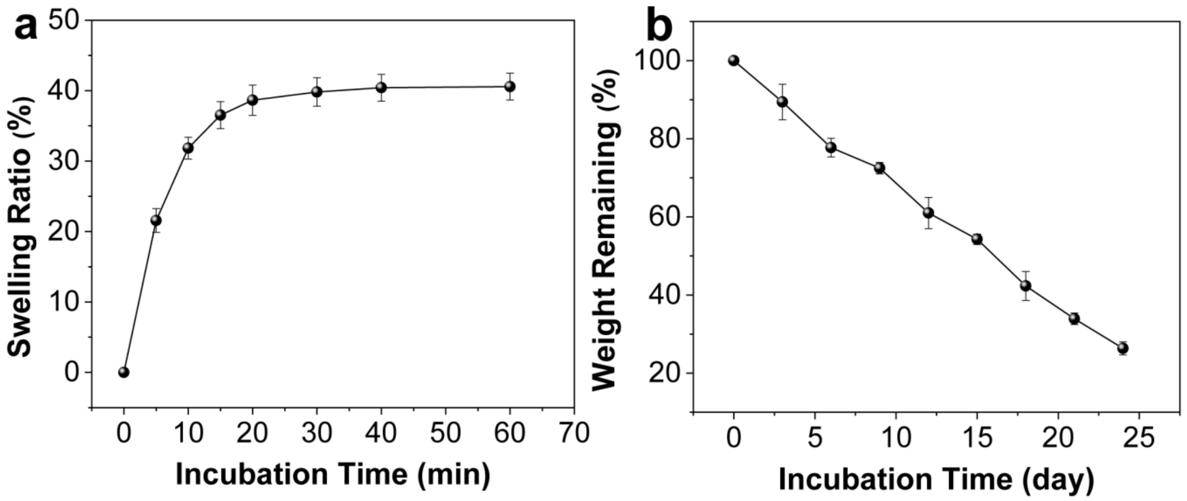

Supplementary Figure 13. **Swelling ratio and weight remaining studies.** (a) Swelling ratio of the hydrogel during the incubation under physiological conditions. (b) Weight remaining of the hydrogel during the incubation under physiological conditions. Data are presented as mean values ± SD (n = 3 independent experiments). Source data are provided as a Source Data file.

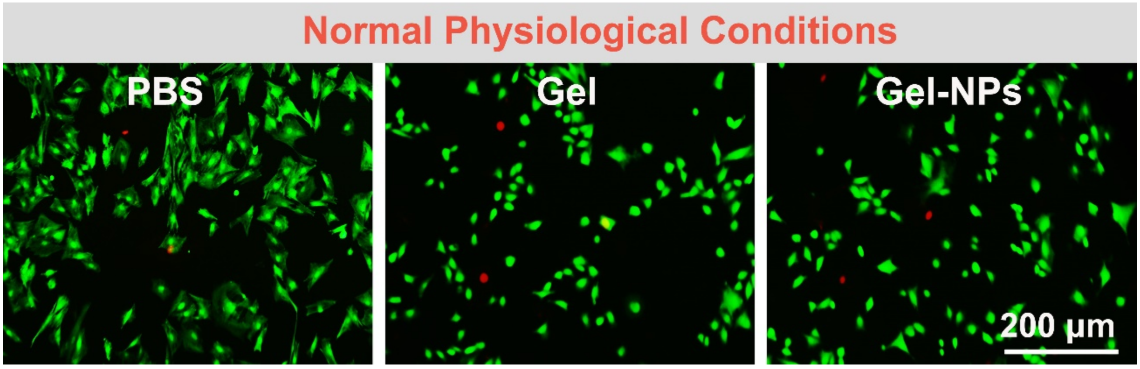

Supplementary Figure 14. Fluorescence images of BMSCs stained with Calcein-AM/PI after treated with PBS, Gel, and Gel-NPs. A representative image of three replicates from each group is shown.

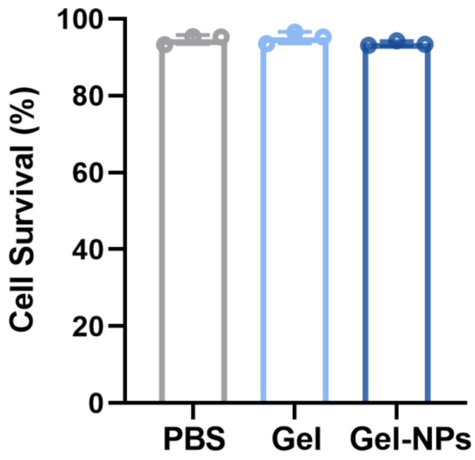

Supplementary Figure 15. Quantitative analysis of BMSCs survival after treated with PBS, Gel, and Gel-NPs. Data are presented as mean values ± SD (n = 3 independent experiments). Source data are provided as a Source Data file.

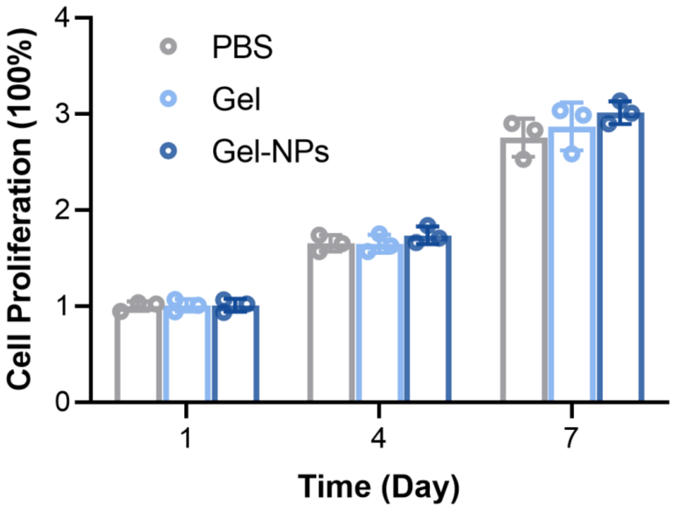

Supplementary Figure 16. Quantitative analysis of BMSCs proliferation after treated with PBS, Gel, and Gel-NPs. Data are presented as mean values ± SD (n = 3 independent experiments). Source data are provided as a Source Data file.

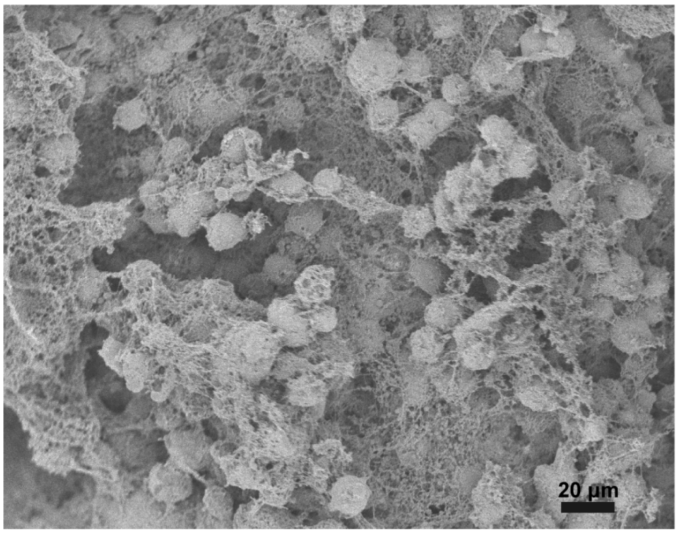

Supplementary Figure 17. SEM image of the lyophilized ε-PLE@MnCoO/Gel hydrogel after encapsulating BMSCs. A representative image of three replicates from each group is shown.

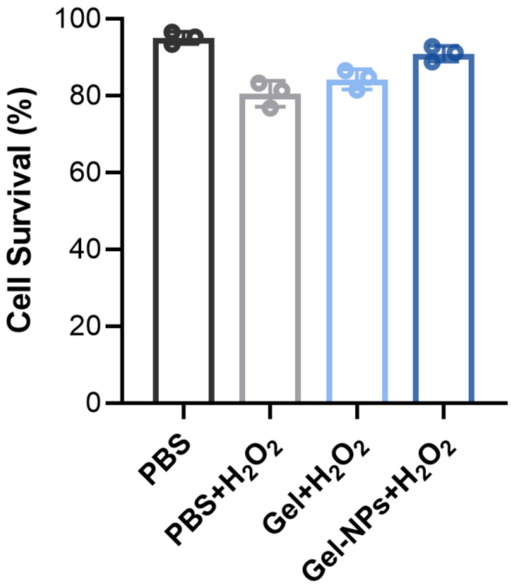

Supplementary Figure 18. Quantitative analysis of BMSCs survival after treated with PBS, PBS+H_2O_2, Gel+H_2O_2, and Gel-NPs+H_2O_2. Quantitatively, the cell survival rate of Gel-NPs group (90.91 ± 2.02%) was significantly improved as compared to PBS+H_2O_2 (80.47 ± 3.36%) and Gel+H_2O_2 (84.24 ± 2.59%). Data are presented as mean values ± SD (n = 3 independent experiments). Source data are provided as a Source Data file.

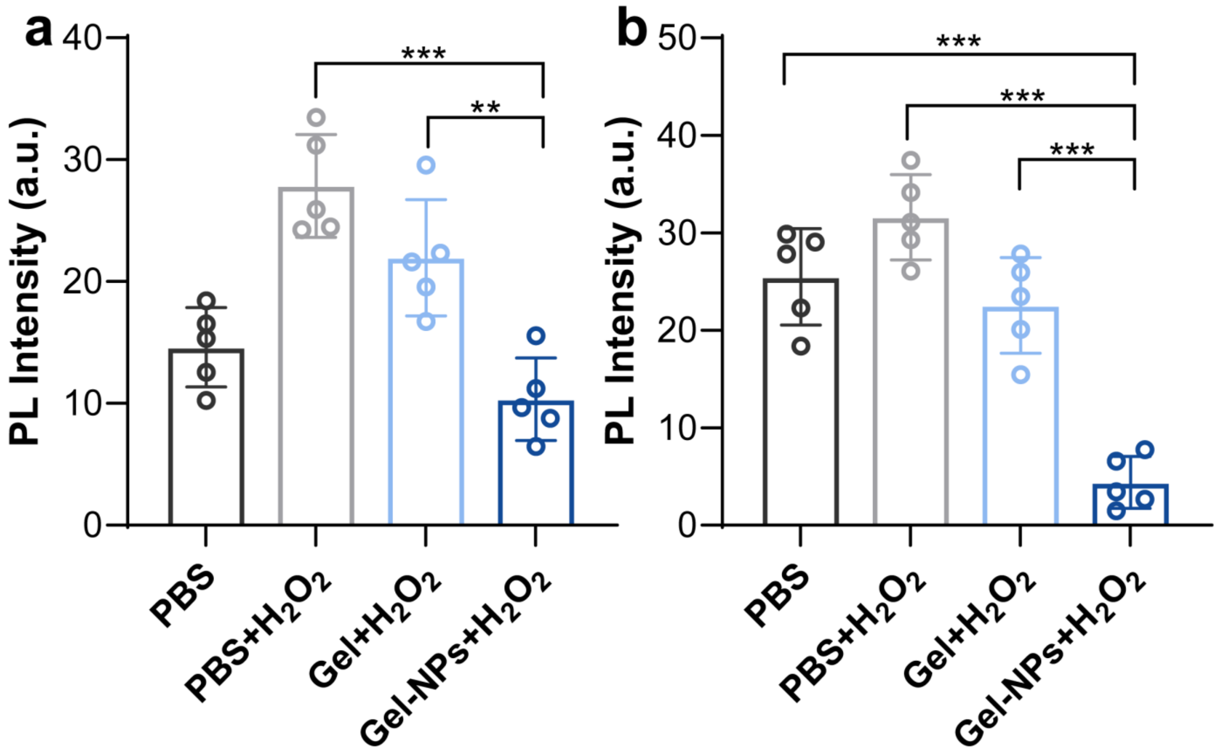

Supplementary Figure 19. **Quantitative analysis of intracellular ROS decomposition and O_2 generation.** (a) Quantitative analysis of intracellular ROS decomposition according to the fluorescent intensity of an ROS indicator (DCFH-DA). Data are presented as mean values ± SD (n = 5 independent experiments). (b) Quantitative analysis of intracellular O_2 generation according to the fluorescent intensity of a typical O_2 level indicator [Ru(dpp)$_3$Cl$_2$]. Throughout, data are presented as mean values ± SD (n = 5 independent experiments), and statistical significance was determined by two-tailed t-test. *$P < 0.05$, **$P < 0.01$, and ***$P < 0.001$. Source data and exact *P* values are provided as a Source Data file. PL: photoluminescence.

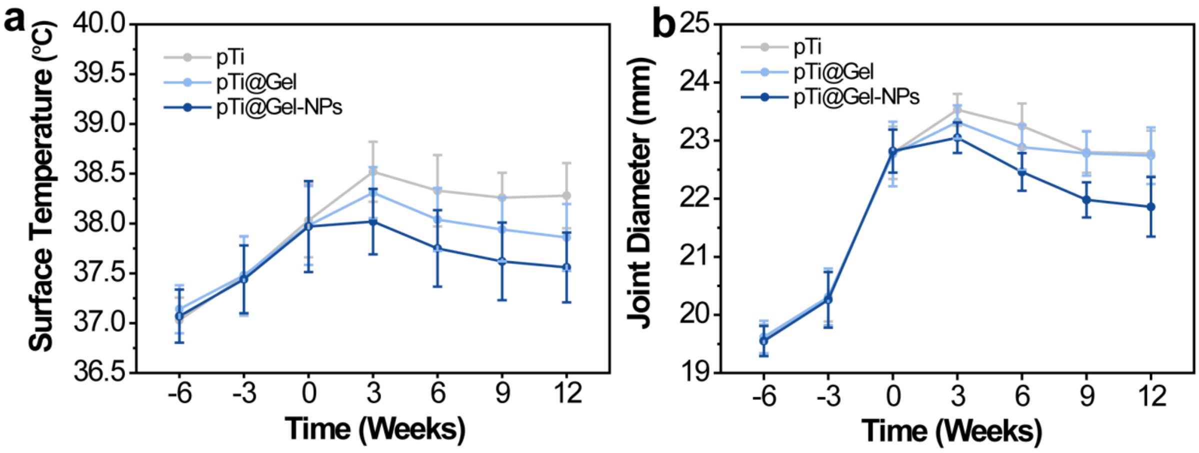

Supplementary Figure 20. **Skin temperature and joint diameter studies.** (a) Skin temperature and (b) joint diameter of RA rabbit implanted with various samples recorded during the whole therapeutic period. Throughout, data are presented as mean values ± SD (n = 5 independent experiments), and statistical significance was determined by two-tailed t-test. Source data and exact *P* values are provided as a Source Data file.

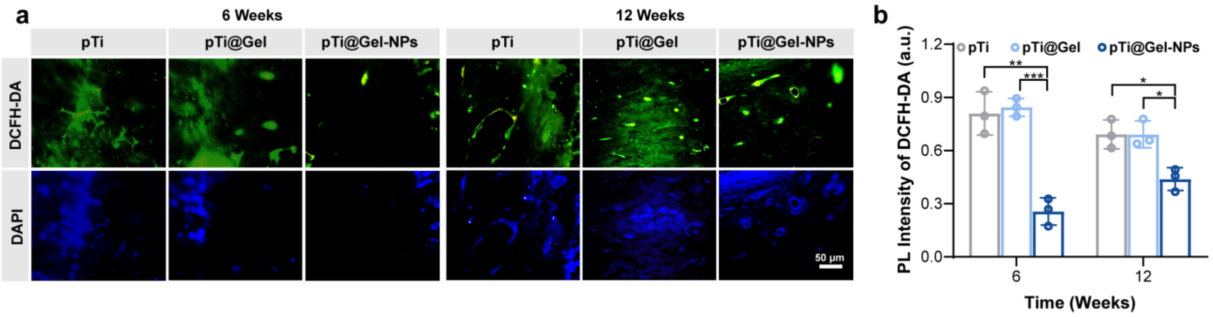

Supplementary Figure 21. **The ROS level in RA bone tissue.** (a) Representative immunofluorescence staining images of DCFH-DA on the bone tissues around the scaffolds at weeks 6 and 12 after different treatmencts. A representative image of three replicates from each group is shown. (b) Quantitative statistics of DCFH-DA on the bone tissue around the scaffolds at week 6 and 12 after different treatments. Data are presented as mean values ± SD (n = 3 independent experiments), and statistical significance was determined by two-tailed t-test. *$P < 0.05$, **$P < 0.01$, and ***$P < 0.001$. Source data and exact *P* values are provided as a Source Data file.

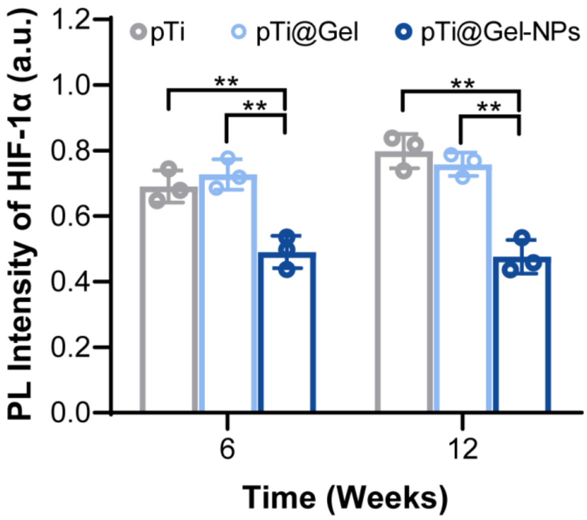

Supplementary Figure 22. Quantitative statistics of HIF-1α on the bone tissue around the scaffolds at week 6 and 12 after different treatments. Data are presented as mean values ± SD (n = 3 independent experiments), and statistical significance was determined by two-tailed t-test. **P* < 0.05, ***P* < 0.01, and ****P* < 0.001. Source data and exact *P* values are provided as a Source Data file.

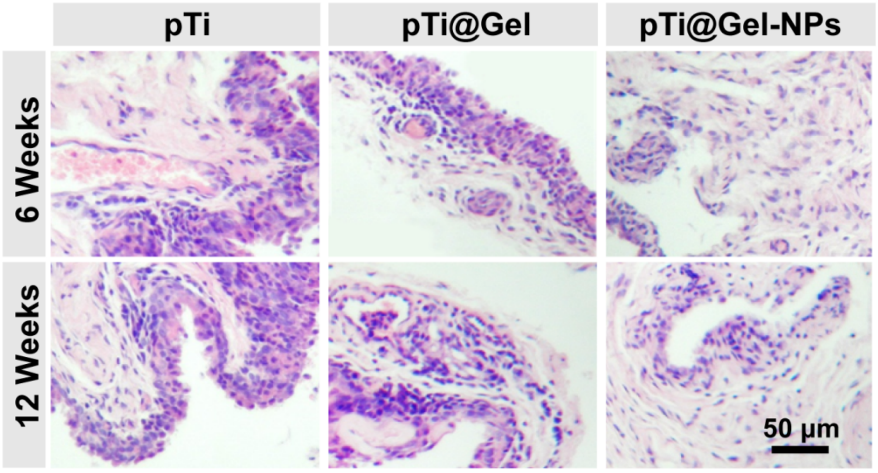

Supplementary Figure 23. Hematoxylin and eosin staining of synovial tissues for RA rabbits receiving various treatments on weeks 6 and 12. A representative image of three replicates from each group is shown.

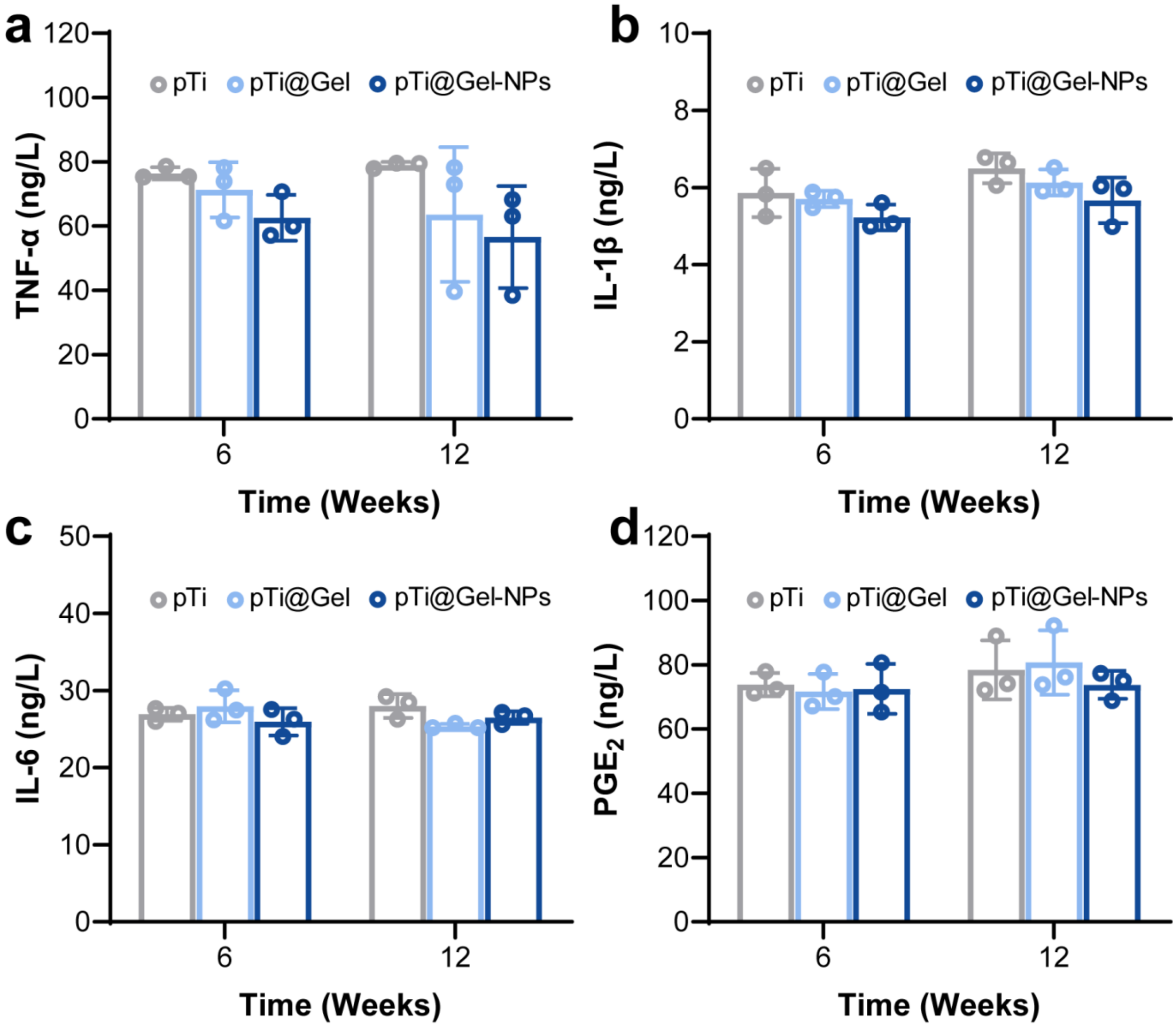

Supplementary Figure 24. **Content of inflammatory cytokines in serum**. (a) TNF-α, (b) IL-1β, (c) IL-6, and (d) PGE_2 data for assessing systemic RA inflammatory state Data are presented as mean values ± SD (n = 3 independent experiments). Source data are provided as a Source Data file.

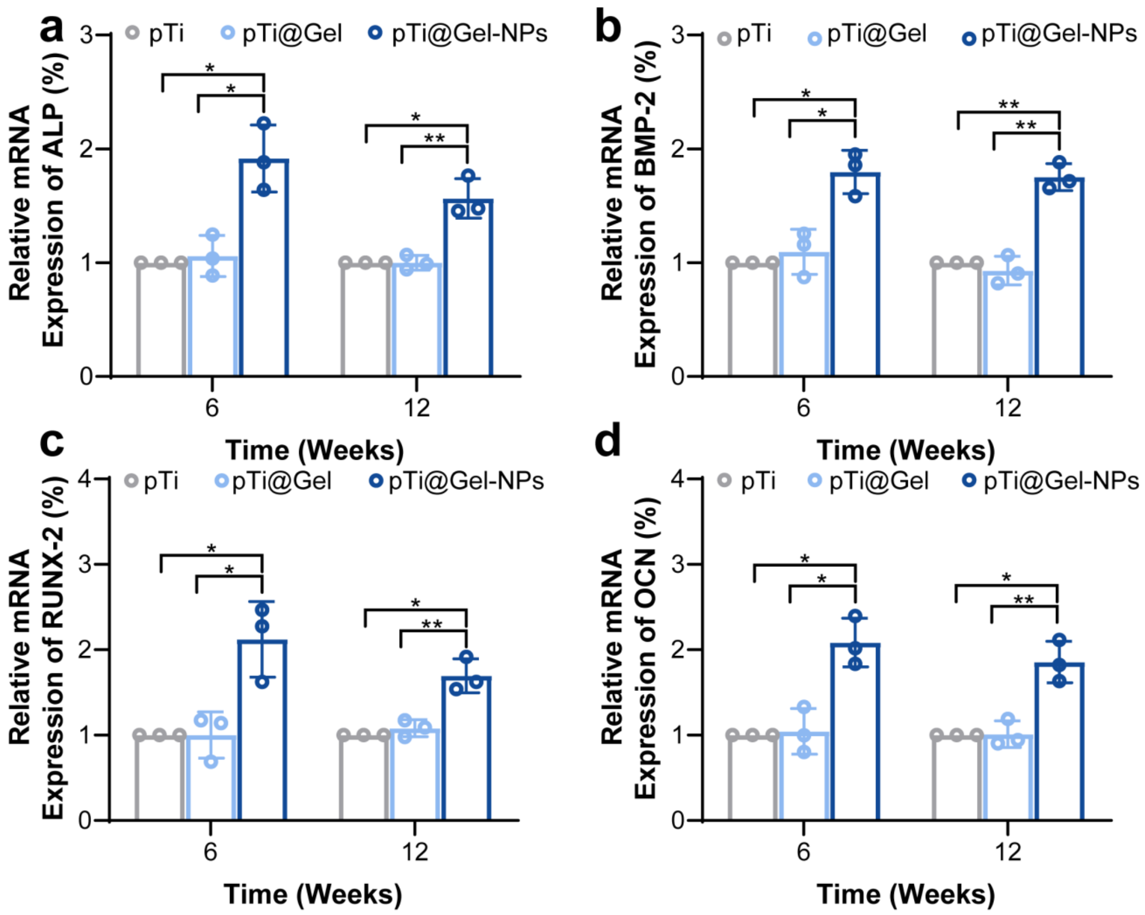

Supplementary Figure 25. **Relative mRNA expression levels.** Relative mRNA expression levels of osteoclastic genes from bone defects at week 6 and 12 after the treatment with pTi, pTi@Gel, and pTi@Gel-NPs scaffolds, including (a) ALP, (b) BMP-2, (c) RUNX-2, and (d) OCN. Throughout, data are presented as mean values ± SD (n = 3 independent experiments), and statistical significance was determined by two-tailed t-test. *$P < 0.05$, **$P < 0.01$, and ***$P < 0.001$. Source data and exact *P* values are provided as a Source Data file.

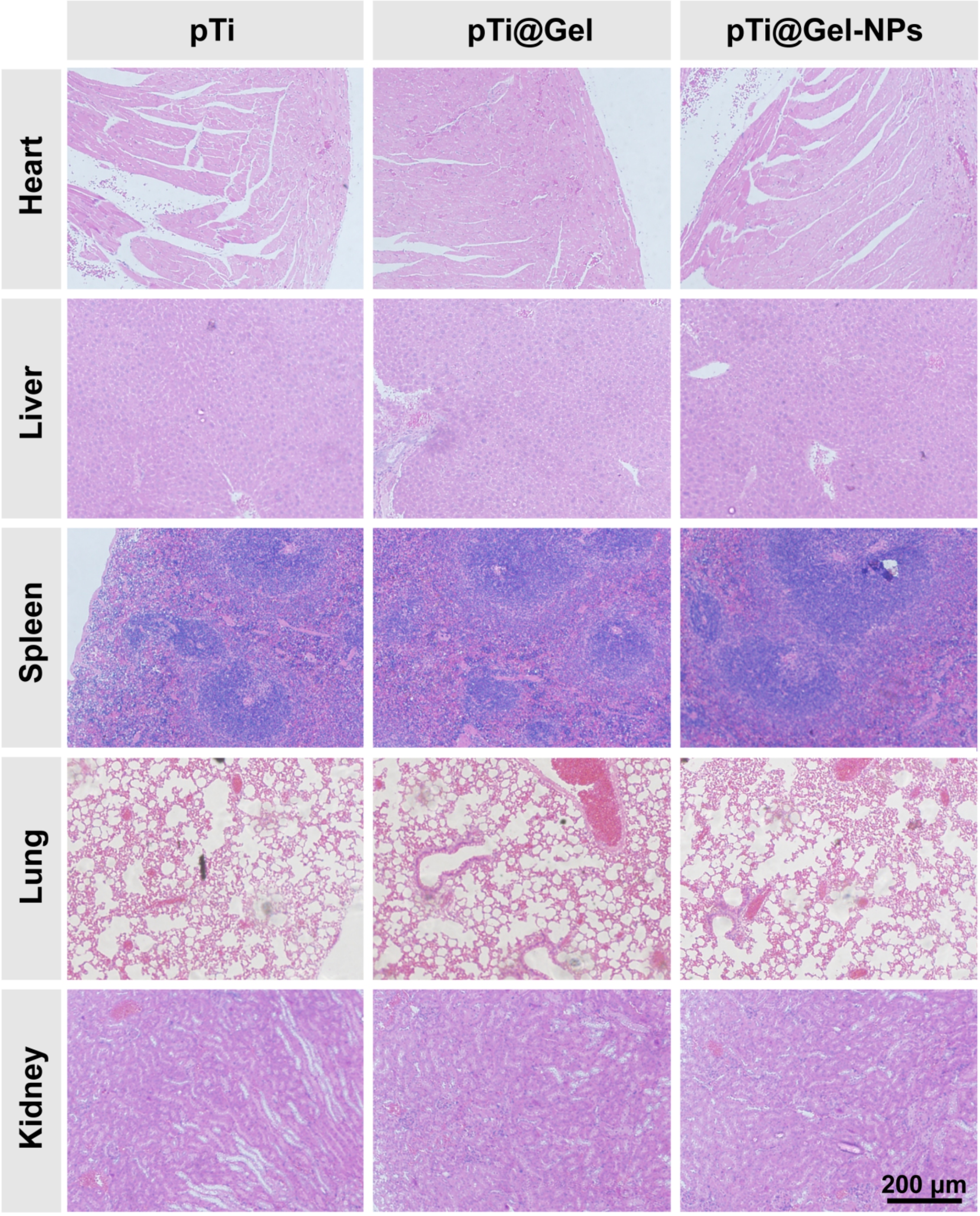

Supplementary Figure 26. Representative histological images for major organs, including heart, liver, spleen, lung, and kidney with H&E at the week 12 after the treatment with pTi, pTi@Gel, and pTi@Gel-NPs scaffolds, respectively. A representative image of three replicates from each group is shown.

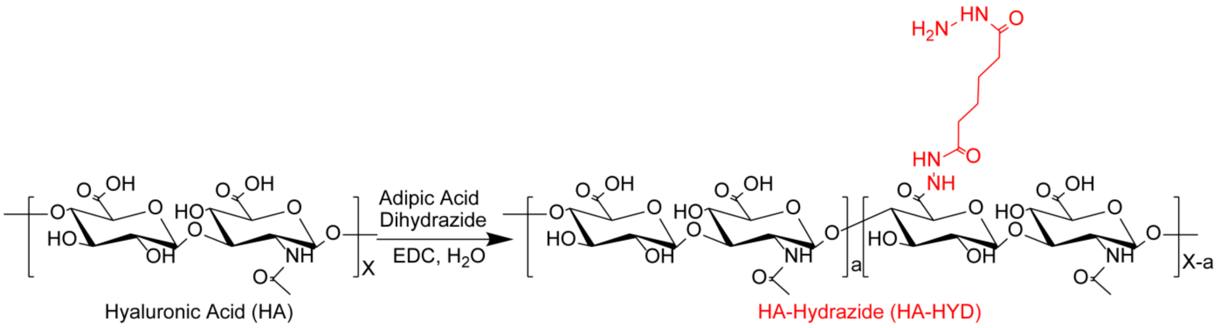

Supplementary Figure 27. Synthesis procedure of HA-HYD.

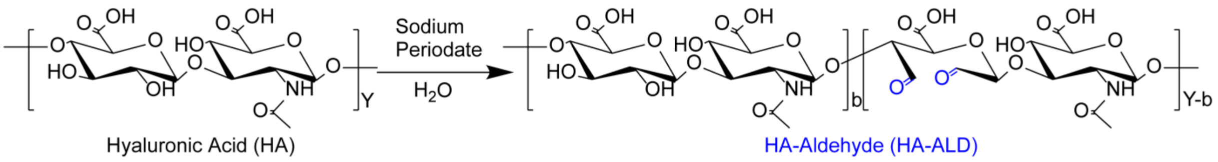

Supplementary Figure 28. Synthesis procedure of HA-ALD.
