# Supplementary material for: Nanozyme-reinforced hydrogel as a H2O2-driven oxygenerator for enhancing prosthetic interface osseointegration in rheumatoid arthritis therapy
Source: Nat Commun. 2022 Nov 9;13:6758. doi: 10.1038/s41467-022-34481-5 (PMC9646710; doi:10.1038/s41467-022-34481-5)
Supplement: Supplementary file 3 — Reporting Summary [file 41467_2022_34481_MOESM3_ESM.pdf]

nature portfolio

Corresponding author(s): Zuhao Li; Jincheng Wang; Xiangzhong Ren; Yanli Zhao

Last updated by author(s): Oct 7, 2022

# Reporting Summary

Nature Portfolio wishes to improve the reproducibility of the work that we publish. This form provides structure for consistency and transparency in reporting. For further information on Nature Portfolio policies, see our Editorial Policies and the Editorial Policy Checklist.

## Statistics

For all statistical analyses, confirm that the following items are present in the figure legend, table legend, main text, or Methods section.

| n/a | Confirmed | |
|---|---|---|
| ☐ | ☒ | The exact sample size (*n*) for each experimental group/condition, given as a discrete number and unit of measurement |
| ☐ | ☒ | A statement on whether measurements were taken from distinct samples or whether the same sample was measured repeatedly |
| ☐ | ☒ | The statistical test(s) used AND whether they are one- or two-sided<br>*Only common tests should be described solely by name; describe more complex techniques in the Methods section.* |
| ☐ | ☒ | A description of all covariates tested |
| ☐ | ☒ | A description of any assumptions or corrections, such as tests of normality and adjustment for multiple comparisons |
| ☐ | ☒ | A full description of the statistical parameters including central tendency (e.g. means) or other basic estimates (e.g. regression coefficient) AND variation (e.g. standard deviation) or associated estimates of uncertainty (e.g. confidence intervals) |
| ☐ | ☒ | For null hypothesis testing, the test statistic (e.g. *F*, *t*, *r*) with confidence intervals, effect sizes, degrees of freedom and *P* value noted<br>*Give P values as exact values whenever suitable.* |
| ☒ | ☐ | For Bayesian analysis, information on the choice of priors and Markov chain Monte Carlo settings |
| ☒ | ☐ | For hierarchical and complex designs, identification of the appropriate level for tests and full reporting of outcomes |
| ☒ | ☐ | Estimates of effect sizes (e.g. Cohen's *d*, Pearson's *r*), indicating how they were calculated |

*Our web collection on statistics for biologists contains articles on many of the points above.*

## Software and code

Policy information about availability of computer code

| | |
|---|---|
| Data collection | Scanning electron microscopy (SEM) images were collected on a microscopy (Thermo scientific, Thermo APREO S). Rheological data were collected with a rheometer (ThermoFisher Scientific, HAAKE MARS 40). Powder X-ray diffraction (XRD) data were collected from a diffractometer (German Bruker, D8ADVANCE). X-ray photoelectron spectroscopy (XPS) data were collected with a spectrometer (Thermo Fisher Scientific, K-Alpha+) equipped with Mg Kα excitation (1253.6 eV). Fourier transform infrared spectroscopy (FT-IR) data were collected on a spectrophotometer (Shimadzu, IR Affinity-1) . UV-Vis absorption data were obtained from a spectrometer (Shimadzu, UV-3600PLUS) . Dynamic laser scattering (DLS) data were collected using a Zetasizer UV spectrometer (Beckman, DelsaMax CORE). |
| Data analysis | Quantitative analysis of immunofluorescence was performed by Image J 1.50i (NIH, Bethesda, MD, USA). Bone morphological parameters of Micro-CT were performed by micro-CT auxiliary software (NRecon, version 1.6.6). Quantitative data were presented as mean ± standard deviation, and conducted from at least triple independent experiments. Error bars represent the standard deviation of measurements within each experiment (*P <0.05, **P < 0.01, ***P < 0.001). Statistical calculations were performed using OriginPro 2019 (64 bit) and graphPad Prism (V.8.0.2). One-way ANOVA was applied for comparisons across multiple groups followed by Tukey's post hoc test using SPSS 19.0 (SPSS Inc., Chicago, USA). |

For manuscripts utilizing custom algorithms or software that are central to the research but not yet described in published literature, software must be made available to editors and reviewers. We strongly encourage code deposition in a community repository (e.g. GitHub). See the Nature Portfolio guidelines for submitting code & software for further information.

## Data

Policy information about availability of data

All manuscripts must include a data availability statement. This statement should provide the following information, where applicable:

- Accession codes, unique identifiers, or web links for publicly available datasets
- A description of any restrictions on data availability
- For clinical datasets or third party data, please ensure that the statement adheres to our policy

The authors declare that all data are available within the Article and Supplementary Files, or available from the corresponding authors upon reasonable request. Source data are available for Figs. 2-7 and Supplementary Figs. 2, 3, 5, 6, 8-13, 15, 16, 18-22, 24 and 25 in the associated Source Data file. Source data are provided with this paper.

## Human research participants

Policy information about studies involving human research participants and Sex and Gender in Research.

| | |
|---|---|
| Reporting on sex and gender | N/A |
| Population characteristics | N/A |
| Recruitment | N/A |
| Ethics oversight | N/A |

Note that full information on the approval of the study protocol must also be provided in the manuscript.

# Field-specific reporting

Please select the one below that is the best fit for your research. If you are not sure, read the appropriate sections before making your selection.

[x] Life sciences   [ ] Behavioural & social sciences   [ ] Ecological, evolutionary & environmental sciences

For a reference copy of the document with all sections, see nature.com/documents/nr-reporting-summary-flat.pdf

# Life sciences study design

All studies must disclose on these points even when the disclosure is negative.

| | |
|---|---|
| Sample size | We use the minimum number of animals (n≥3), depending on each experiment, necessary to achieve statistical significance. The detailed sample size for each experiment is shown in all the figure legends. |
| Data exclusions | No data was excluded. |
| Replication | Experiments were repeated at least three independent experiments with similar results. All experiments were reproduced to reliably support conclusions stated in the manuscript. |
| Randomization | All the tests were conducted with randomly allocated experimental groups. |
| Blinding | The investigators were blinded to group allocation during data collection and analysis. |

# Reporting for specific materials, systems and methods

We require information from authors about some types of materials, experimental systems and methods used in many studies. Here, indicate whether each material, system or method listed is relevant to your study. If you are not sure if a list item applies to your research, read the appropriate section before selecting a response.

## Materials & experimental systems

| n/a | Involved in the study |
| --- | --- |
| ☐ | ☒ Antibodies |
| ☐ | ☒ Eukaryotic cell lines |
| ☒ | ☐ Palaeontology and archaeology |
| ☐ | ☒ Animals and other organisms |
| ☒ | ☐ Clinical data |
| ☒ | ☐ Dual use research of concern |

## Methods

| n/a | Involved in the study |
| --- | --- |
| ☒ | ☐ ChIP-seq |
| ☒ | ☐ Flow cytometry |
| ☒ | ☐ MRI-based neuroimaging |

# Antibodies

Antibodies used

Primary antibodies for tissue immunofluorescence/immunohistochemistry:
Primary antibodies of mouse monoclonal 4-HNE, rabbit monoclonal TNF-α, and rabbit polyclonal PGE2 were purchased from were purchased from Abcam (Cambridge, UK). Primary antibodies of rabbit polyclonal IL-1β and rabbit polyclonal HIF-1α were purchased from Affinity Biosciences LTD (Jiangsu, China). Primary antibody of rabbit polyclonal 8-OHdG was supplied by Bioss Antibodies (Beijing, China). Primary antibody of rabbit polyclonal IL-6 was supplied by Cell Signaling Technology (Massachusetts, USA)
Primary antibody of mouse monoclonal 4-HNE, at 1:150 dilution: Anti-4 Hydroxynonenal antibody [HNEJ-2], Mouse monoclonal [HNEJ-2] to 4 Hydroxynonenal, Cat. No. ab48506.
Primary antibody of rabbit polyclonal 8-OHdG, at 1:100 dilution: Rabbit polyclonal, Cat. No. bs-1278R
Primary antibody of rabbit polyclonal IL-1β, at 1:150 dilution: Rabbit polyclonal, Cat. No. AF5103
Primary antibody of rabbit monoclonal TNF-α, at 1:100 dilution: Recombinant Anti-TNF alpha antibody [EPR19147], Rabbit monoclonal [EPR19147] to TNF alpha, Cat. No. ab183218.
Primary antibody of rabbit polyclonal PGE2, , at 1:100 dilution: SureLight® APC Anti-PGE2 receptor EP4 subtype antibody, SureLight® APC Rabbit polyclonal to PGE2 receptor EP4 subtype, Cat. No. ab92763.
Primary antibody of rabbit polyclonal IL-6,  at 1:150 dilution: IL-6 (D5W4V) XP® Rabbit mAb (Mouse Specific) Cat. No. #12912.
Primary antibody of rabbit polyclonal HIF-1α, , at 1:200 dilution: Rabbit polyclonal, Cat. No. AF1009.

Validation

All antibodies are commercially available and have been tested by the manufacturer. Vendors and catalogue numbers are listed above, and validation information can be found on the manufacturer's website:

https://www.abcam.com/4-hydroxynonenal-antibody-hnej-2-ab48506.html
http://www.bioss.com.cn/prolook_03.asp?id=AF08169606000963&pro37=1
http://www.affbiotech.cn/goods-4410-AF5103-IL1_beta_Antibody.html
https://www.abcam.com/tnf-alpha-antibody-epr19147-ab183218.html
https://www.abcam.com/surelight-apc-pge2-receptor-ep4-subtype-antibody-ab92763.html
https://www.cellsignal.com/products/primary-antibodies/il-6-d5w4v-xp-rabbit-mab-mouse-specific/12912?site-search-type=Products&N=4294956287&Ntt=%2312912&fromPage=plp&_requestid=43941
http://www.affbiotech.cn/goods-2076-AF1009-HIF1A_Antibody.html

# Eukaryotic cell lines

Policy information about cell lines and Sex and Gender in Research

| | |
| --- | --- |
| Cell line source(s) | Rabbit BMSCs were supplied by Procell (CP-Rb007). |
| Authentication | The suppliers routinely authenticate the cell lines by short tandem repeat profiling though the cell lines were not authenticated by our laboratory. |
| Mycoplasma contamination | Mycoplasma test was negative. |
| Commonly misidentified lines (See ICLAC register) | No commonly misidentified cell lines was used. |

# Animals and other research organisms

Policy information about studies involving animals; ARRIVE guidelines recommended for reporting animal research, and Sex and Gender in Research

| | |
| --- | --- |
| Laboratory animals | Thirty-six male New Zealand white rabbits (2.5-3.0 kg, 5-month-old) were purchased from Jilin University and kindly kept in the Laboratory Animal Center of Jilin University. We use the minimum number of animals (n≥3), depending on each experiment, necessary to achieve statistical significance. The detailed sample size for each experiment is shown in all the figure legends. |
| Wild animals | The study did not involve wild animals. |

| | |
|---|---|
| Reporting on sex | To more convincingly prove the advantages, we conducted the certification in a severe RA male rabbit model induced by subcutaneously injecting ovalbumin (OVA) and Freund's adjuvant, and a cylindrical bone defect was prepared on the distal femur tissues to mimic large-scale bone destruction of RA. A male rabbit is suitable for the establishment of the RA model according to literatures, while the level of estrogen of the female rabbit has an influence on the development of the RA model. |
| Field-collected samples | No field-collected samples was used. |
| Ethics oversight | All animal procedures were conducted according to the guidelines for Care and Use of Laboratory Animal Experience of Jilin University and approved by the Animal Ethics Committee of Jilin University. |

Note that full information on the approval of the study protocol must also be provided in the manuscript.
