# Supplementary material for: Nanozyme-reinforced hydrogel as a H2O2-driven oxygenerator for enhancing prosthetic interface osseointegration in rheumatoid arthritis therapy
Source: Nat Commun. 2022 Nov 9;13:6758. doi: 10.1038/s41467-022-34481-5 (PMC9646710; doi:10.1038/s41467-022-34481-5)
Supplement: Supplementary file 2 — Peer Review File [file 41467_2022_34481_MOESM2_ESM.pdf]

nature portfolio

Peer Review File

Nanozyme-Reinforced Hydrogel as a H_2O_2-Driven Oxygenerator for Enhancing Prosthetic Interface Osseointegration in Rheumatoid Arthritis Therapy

Open Access This file is licensed under a Creative Commons Attribution 4.0 International License, which permits use, sharing, adaptation, distribution and reproduction in any medium or format, as long as you give appropriate credit to the original author(s) and the source, provide a link to the Creative Commons license, and indicate if changes were made. In the cases where the authors are anonymous, such as is the case for the reports of anonymous peer reviewers, author attribution should be to 'Anonymous Referee' followed by a clear attribution to the source work. The images or other third party material in this file are included in the article's Creative Commons license, unless indicated otherwise in a credit line to the material. If material is not included in the article's Creative Commons license and your intended use is not permitted by statutory regulation or exceeds the permitted use, you will need to obtain permission directly from the copyright holder. To view a copy of this license, visit http://creativecommons.org/licenses/by/4.0/.

REVIEWER COMMENTS</B>

Reviewer #1 (Remarks to the Author):

The manuscript written by Zhao et al. developed a biological nanozyme-reinforced hydrogel, showing promising potentials for synergetic effects with BMSCs in the treatment against RA. Overall, the manuscript is well organized, and the authors provide significant amounts of data obtained from experiments that have been carefully conducted. However, the reviewer has several concerns about some detailed issues, which requires further explanations or supplying more data by the authors.

Major concerns
1. Page 7, line 157. More clarifications are suggested to show the reasons of using HA-HYD (2.5 wt%), ε-PLE@MnCoO (1 mg mL-1), and HA-ALD (5 wt%) as the representative preparation. Is the optimization only according to the mechanical property?
2. What is the porosity, swelling property and degradation of the gel?
3. SEM of the gel after encapsulating MSCs should be demonstratred.
4. The safety of the transplanted gel is suggested to evaluate.
5. In Fig. 2, 1.0 M H2O2 was selected for the evaluations in A to C, 0.1 M H2O2 was used in E and F. What are the reasons of using different concentrations of H2O2?
6. Will the O2 generation causes some adverse impacts on BMSCs? This point is suggested to evaluate, because the overhigh O2 levels may also affect the behavior of BMSCs.
7. In Fig. 3, the concentration of H2O2 was demonstrated as 100 μM. It is suggested to use the uniform unit (0.1 M or 100 μM) for the convenience of readers. Additionally, why this concentration of H2O2 was selected for the evaluation? Is this concentration can represent the ROS levels in RA?
8. Fig. 3D, the O2 generation looks similar between the groups of PBS (control) and Gel+ H2O2.
9. Fig. 5, comparisons to evaluate therapeutic effects of using pTi@Gel-NPs without loading BMSCs and using BMSCs alone without the gel are suggested.
10. Will the loaded BMSCs be released from the gel in articulation? The reviewer feels that the therapeutic effect more attributes to the osteogenic bioactivity through the paracrine of MSCs instead of the osteogenic differentiation. More evidence to determine how encapsulated BMSCs facilitated the bone reconstruction are suggested.
11. Authors only evaluated the local inflammatory levels. Will this strategy also affect the systemic immune response, especially considering RA is caused by autoimmune disorders?

Minor suggestions:
1. It is suggested to say "… was investigated" instead of "… was systematically investigated". Each result should be systematically investigated.
2. It is suggested to show the repeats number of each data directly in figure legends.

Reviewer #2 (Remarks to the Author):

Nanozymes are functional nanomaterials with enzyme mimicking activities. They have been explored for various disease therapy. In the manuscript, the authors developed a novel nanozyme-reinforced hydrogel, which could alleviate the symptoms of RA by regulating inflammatory cytokines and improved the bone regeneration in the in vivo model. This work has broadened the biomedical applications of nanozymes and would inspire others to explore the wide promise of nanozymes as well as other bionanomaterials. Therefore, I would suggest the publication after addressing the following concerns.
1. Why did the authors select MnCoO nanozyme as the catalase-mimic? How about other nanozyme with catalase-like activity? How about the stability of MnCoO nanozyme? Would Mn2+ and Co2+ be released from the MnCoO nanozyme?

2. What's the concentration of H2O2 in RA pathological microenvironment? Why did the authors chose 1M H2O2 to test the catalase-like catalytic activity?
3. It stated that the produced dissolved oxygen could improve the poor oxygen supply in RA pathological microenvironment. How about the expression levels of HIF-1α (hypoxia-inducible factor)?
4. In RA model, the serious joint swelling behaviors would be observed. How about the improvement effect?
5. To exhibit the regenerated cartilage better and articular morphology, the Safranin-fixed green staining of joints could be performed.
6. Since it is a study on nanozymes, several closely related publications could be cited if possible. For example,
(a) Chemical Society Reviews, 2019, 48, 1004-1079.
(b) Nano Letters, 2022, 22, 508-516.

REVIEWER COMMENTS

Reviewer #1 (Remarks to the Author):

The manuscript written by Zhao et al. developed a biological nanozyme-reinforced hydrogel, showing promising potentials for synergetic effects with BMSCs in the treatment against RA. Overall, the manuscript is well organized, and the authors provide significant amounts of data obtained from experiments that have been carefully conducted. However, the reviewer has several concerns about some detailed issues, which requires further explanations or supplying more data by the authors.

Authors' responses: We thank the reviewer for the useful comments and suggestions. We have revised the manuscript accordingly.

Major concerns

1. Page 7, line 157. More clarifications are suggested to show the reasons of using HA-HYD (2.5 wt%), ε-PLE@MnCoO (1 mg mL^{-1}), and HA-ALD (5 wt%) as the representative preparation. Is the optimization only according to the mechanical property?

Authors' responses: We thank Reviewer 1 for the questions. It was found in one research study that stiff hydrogel scaffolds stimulate human mesenchymal stem cells (hMSCs) towards osteoblast differentiation, while on soft gels cells differentiate into adipocytes.[1] In our system, the mechanical property of nanozyme-reinforced hydrogels can be easily adjusted by varying the concentration of individual components. We selected HA-HYD (2.5 wt%), ε-PLE@MnCoO (1 mg mL^{-1}), and HA-ALD (5 wt%) as a typical representative, in which the mechanical property is similar to those of native bone marrow of human body. According to literature, the elastic moduli of native bone marrow can be monitored from fracture haematomas from patients with the range of 1 to 10 kpa (Fig. 1 to Response).[2] Besides, the hydrogel with the range of mechanical properties is more favorable for BMSCs osteogenic differentiation, as evidenced by a comparison of the osteogenic markers (ALP and Col I) of BMSCs in our previous work.[3]

The nanozyme-reinforced hydrogel with this formula also disclosed a homogenous porous structure with average pore diameters of 200 μm (Fig. 2c), and scaffold materials with aperture sizes of 200–350 μm have been reported to promote bone tissue growth.[4, 5] When BMSCs were encapsulated into the hydrogel, the nanochannels of hydrogel are beneficial for the transportation of nutrients and oxygen, thereby enhancing cell growth and communication. We also compared the SEM image of hydrogel fabricated from different formula, and the results of Fig. 2 to Response demonstrated that the representative hydrogel has suitable aperture size. Moreover, we supplied more characterizations including the porosity, swelling property, and degradation of the hydrogel according to the reviewer's kindly suggestions. All the above data suggest that the nanozyme-reinforced hydrogel with this formula can recapitulate many critical aspects of the native extracellular matrix, and significantly enhance the therapeutic efficacy of stem cells.

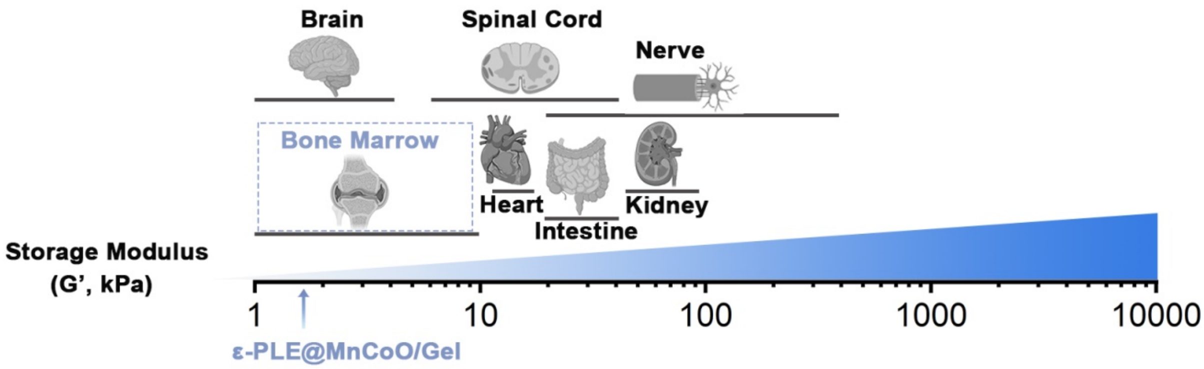

Fig. 1 to Response: Schematic illustration for the storage modulus (G') of various tissues and the ε-PLE@MnCoO/Gel hydrogel with formula of HA-HYD (2.5 wt%), ε-PLE@MnCoO (1 mg mL^{-1}), and HA-ALD (5 wt%).

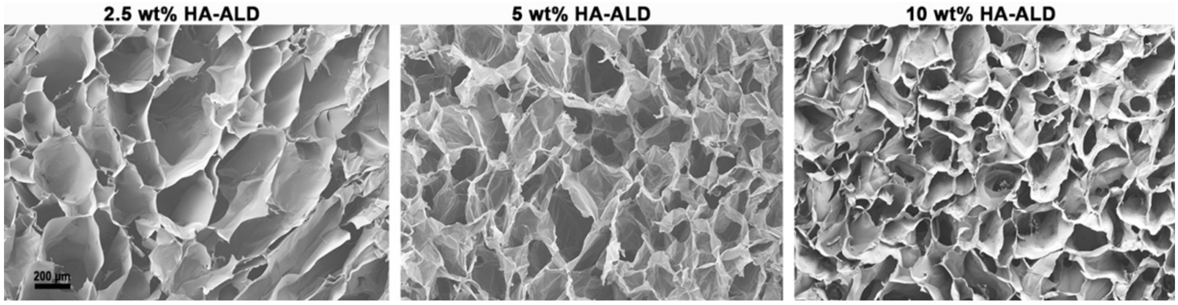

Fig. 2 to Response: SEM image of the lyophilized ε-PLE@MnCoO/Gel hydrogel made from HA-HYD (2.5 wt%), ε-PLE@MnCoO (1 mg mL^{-1}), and HA-ALD (2.5, 5 and 10 wt%).

References

1. Trappmann, B., *et al.* Extracellular-matrix tethering regulates stem-cell fate. *Nat. Mater.* **11**, 642-649 (2012).
2. Vining KH, *et al.* Mechanical checkpoint regulates monocyte differentiation in fibrotic niches. *Nat. Mater.* (2022). doi: 10.1038/s41563-022-01293-3.
3. Zhao, Y.*, et al.* Biomimetic composite scaffolds to manipulate stem cells for aiding rheumatoid arthritis management. *Adv. Funct. Mater.* **29**, 1807860 (2019).
4. Murphy, C. M., Haugh, M. G., O' Brien, F. J. The effect of mean pore size on cell attachment, proliferation and migration in collagen–glycosaminoglycan scaffolds for bone tissue engineering. *Biomaterials* **31**, 461-466 (2010).
5. Yi, H., Ur Rehman, F., Zhao, C., Liu, B., He, N. Recent advances in nano scaffolds for bone repair. *Bone Res.* **4**, 16050 (2016).

2. What is the porosity, swelling property and degradation of the gel?

Authors' responses: Thanks for the reviewer's kind suggestions, which are valuable for improving the accuracy of the manuscript. Considering the suggestion, we have supplemented the swelling and degradation of the gel in the revised manuscript as well as listed as follows.

Microstructural observation of SEM demonstrated that the hydrogels had porous network structures. Moreover, we supplied the porosity of the ε-PLE@MnCoO/Gel hydrogel

by the alcohol displacement method. Details of data and discussions have been provided in the revised manuscript as well as listed as follows.

"**Porosity of Hydrogels**: The porosity of ε-PLE@MnCoO/Gel hydrogel was evaluated based on the ethanol displacement method. Briefly, the completely gelled ε-PLE@MnCoO/Gel hydrogel was prepared and weighed (W_1) before immersing in absolute ethanol. After reaching saturation in ethanol, the hydrogel was collected and weighed (W_2). Thereafter, the porosity of hydrogel was calculated via the following equation: Porosity = $(W_1 - W_2) / \rho V \times 100\%$, where ρ is the density of absolute ethanol (ρ = 0.789 g cm^{-3}) and V is the volume of the hydrogel."

"The freeze-dried ε-PLE@MnCoO/Gel sample possessed a porous network structure (Fig. 2c), with porosity of nearly 96% (Supplementary Table 1). This structural feature allows the ε-PLE@MnCoO/Gel with abundant-water content and a high swelling ratio under physiological conditions (Supplementary Fig. 13a), which would be beneficial to the cellular metabolism for improving the survival and proliferation of engineered cells."

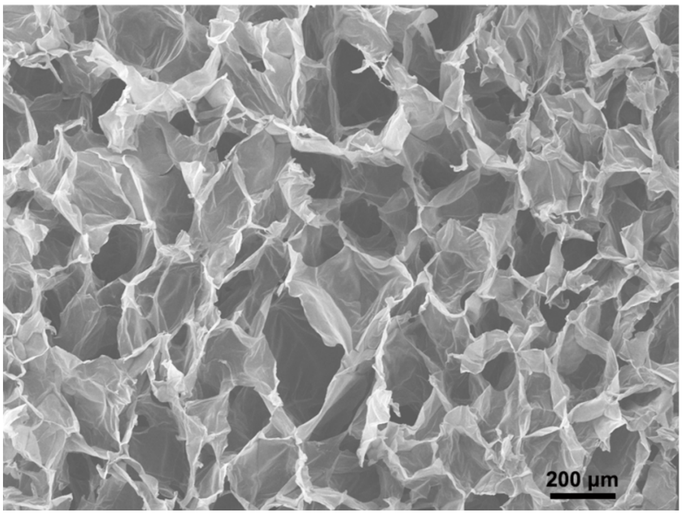

Fig. 2 (c) SEM image of the lyophilized ε-PLE@MnCoO/Gel hydrogel.

"The biodegradability of hydrogels also plays a crucial role in bone tissue applications. To explore in vitro degradation ability of the hydrogel, a quantitative survey of the change in dry weight of hydrogels was further investigated under physiological conditions (i.e., in phosphate buffered saline at 37 °C). It was observed that the ε-PLE@MnCoO/Gel hydrogel could undergo more than 50% weight loss on the 18th day, and the degraded mass percentage was nearly 75% on the 24th day, demonstrating the ε-PLE@MnCoO/Gel hydrogel gradually degraded as the incubation time increased (Supplementary Fig. 13b). The degradation of hydrogels was conducive to stem cell proliferation, migration, and remodeling of the synthetic matrix, finally providing space for the ingrowth of new bone. Taken together, these results suggested that the ε-PLE@MnCoO/Gel satisfied the requirements for cell culture and offered a substantial clinical advantage."

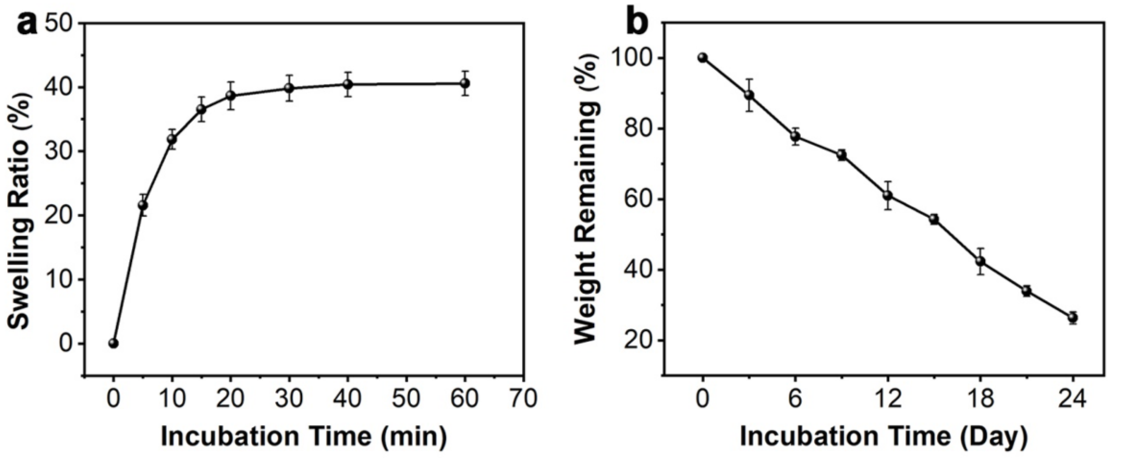

Supplementary Fig. 13. **Swelling ratio and weight remaining studies.** (a) Swelling ratio of the hydrogel during the incubation under physiological conditions. (b) Weight remaining of the hydrogel during the incubation under physiological conditions. (n = 3 independent experiments).

"**Swelling Ratio of Hydrogels**: The swelling ratio of ε-PLE@MnCoO/Gel hydrogel was examined by recording the change in wet weight. First, the completely gelled ε-PLE@MnCoO/Gel hydrogel (500 μL) was immersed in PBS (5 mL) solution at 37 °C. At predetermined time points (0, 5, 10, 15, 20, 30, 40, and 60 min), the hydrogel was withdrawn from the solution, and excess water was wiped off using filter paper. Afterwards, the ε-PLE@MnCoO/Gel hydrogel was weighed, and the swelling ratio was appraised using the following equation: Swelling ratio = $(W_t - W_0) / W_0 \times 100\%$, where W_t represents the weight of hydrogel after incubation in PBS solution, and W_0 means the initial weight of hydrogel.

In Vitro Degradation Ability of Hydrogels: In vitro degradation performance of ε-PLE@MnCoO/Gel hydrogel was measured by recording the change in dry weight. In detail, the completely gelled ε-PLE@MnCoO/Gel hydrogel was lyophilized, and then dried hydrogel (50 mg) was incubated in PBS solution at 37 °C. To reduce bacterial growth, sodium azide (0.02 wt%) was added to the above solution. At predetermined time points (0, 3, 6, 9, 12, 15, 18, 21, 24, 27, and 30 days), the residual hydrogel was collected and washed with deionized water three times. Followed that, the hydrogel was lyophilized, and the weight remaining was calculated according to the following equation: Weight remaining = $1 - (W_0 - W_d) / W_0 \times 100\%$, where W_0 is the initial dry weight of hydrogel, and W_d represents the weight of the hydrogel after degradation at different time points."

3. SEM of the gel after encapsulating MSCs should be demonstrated.

Authors' responses: Thank the reviewer for the constructive comments and suggestions. We have supplemented the SEM image of the lyophilized ε-PLE@MnCoO/Gel hydrogel after encapsulating BMSCs, and more descriptions have been included in the revised manuscript as well as listed as follows.

"Moreover, the BMSCs were distributed uniformly in the ε-PLE@MnCoO/Gel hydrogel matrix, and they maintained intact morphology without obvious damage (Supplementary Figs. 17). These results confirmed that the ε-PLE@MnCoO/Gel hydrogel had no obvious cytotoxicity to BMSCs."

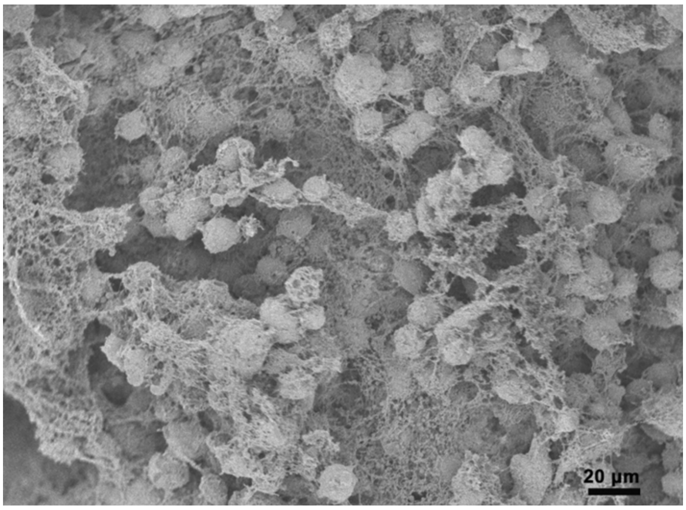

Supplementary Fig. 17. SEM image of the lyophilized ε-PLE@MnCoO/Gel hydrogel after encapsulating BMSCs.

4. The safety of the transplanted gel is suggested to evaluate.

Authors' responses: We appreciate the Reviewer's constructive suggestions, which have helped improve our manuscript. To verify the in vivo safety of hydrogels, we have supplemented the visceral slices of animals after transplantation of experimental groups. Details of data and discussions have been provided in the revised manuscript as well as listed as follows.

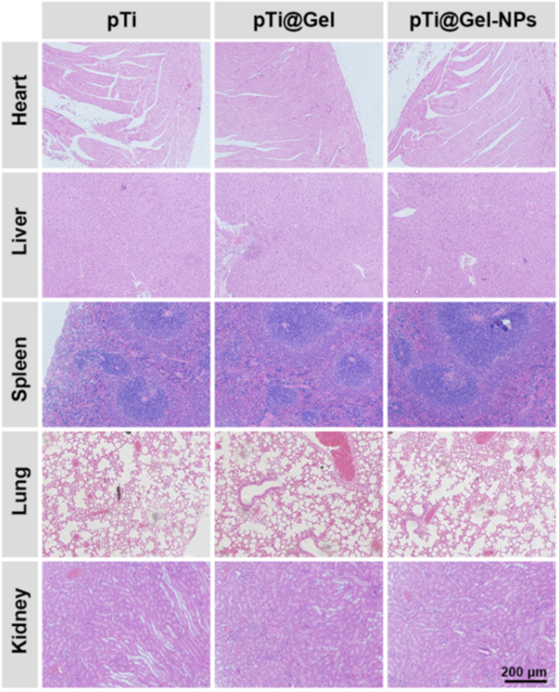

Supplementary Fig. 26. Representative histological images for major organs, including heart, liver, spleen, lung, and kidney with H&E at the week 12 after the treatment with pTi, pTi@Gel, and pTi@Gel-NPs scaffolds, respectively.

"Finally, the biosafety of the transplanted hydrogels was evaluated by H&E staining observation of major organs including the heart, liver, spleen, lung, and kidney, and there was no obvious organ damage or inflammation in the experimental groups (Supplementary Fig. 26). These results suggested the in vivo good biocompatibility of scaffolds, which promised their efficacy and long-term biosafety for clinical applications."

5. In Fig. 2, 1.0 M H_2O_2 was selected for the evaluations in A to C, 0.1 M H_2O_2 was used in E and F. What are the reasons of using different concentrations of H_2O_2?

Authors' responses: Thank you for your question. In the RA pathological microenvironment, oxidative stress is regarded as a crucial mechanism in the initial and progressed phases of destructive proliferative synovitis. According to the previous reports, the extracellular concentration of H_2O_2, as the most representative ROS, reached at a concentration of 1.0 mM under pathological inflammatory conditions, which was estimated to be up to 100-fold higher than healthy tissue.[1-3] Therefore, we selected 1 mM H_2O_2 to test the catalase-like catalytic activity rather than 1 M as indicated in the section of Methods. The concentration was chosen for imitating the H_2O_2-rich RA hostile microenvironment since the H_2O_2 level has been described as high as 1.0 mM under pathological inflammatory conditions. We are very sorry for our mistakes and have changed it to right in the revised Figs. 3a-c.

For the revised Figs. 3e-f, we chose 0.1 mM H_2O_2 to investigate the H_2O_2-driven oxygenation ability of the nanozyme-reinforced hydrogel. We selected a lower concentration of H_2O_2 than that of in revised Figs. 3a-c, since the measuring range of the oxygen probe (PBJ-608 portable dissolved oxygen meter, Shanghai REX Instrument Factory) is 0~19.9 $mg \cdot L^{-1}$ oxygen in an aqueous solution. To promise that the concentration of generated oxygen is within the measurement range, a relatively low concentration of H_2O_2 (0.1 mM) should be applied as a substrate.

References:

1. Peiró Cadahía, J., *et al.* Synthesis and evaluation of hydrogen peroxide sensitive prodrugs of methotrexate and aminopterin for the treatment of rheumatoid arthritis. *J. Med. Chem.* **61**, 3503-3515 (2018).
2. Kumar, R., *et al.* Mitochondrial induced and self-monitored intrinsic apoptosis by antitumor theranostic prodrug: in vivo imaging and precise cancer treatment. *J. Am. Chem. Soc.* **136**, 17836-17843 (2014).
3. Weinstain, R., Savariar, E. N., Felsen, C. N., Tsien, R. Y. In vivo targeting of hydrogen peroxide by activatable cell-penetrating peptides. *J. Am. Chem. Soc.* **136**, 874-877 (2014).

6. Will the O_2 generation causes some adverse impacts on BMSCs? This point is suggested to evaluate, because the over-high O_2 levels may also affect the behavior of BMSCs.

Authors' responses: Thanks for your comment. We agree with the reviewer that over-high O_2 levels may affect the behaviour of BMSCs. However, expanding the O_2 level to study the BMSCs behaviour is neither feasible, given the noticeable toxicity of over-high H_2O_2 levels involved, nor would significantly support our argument. In our system, the oxygenation performance of the nanozyme-reinforced hydrogel is directly driven by H_2O_2, and a high concentration of H_2O_2 is necessary for obtaining the over-high O_2 level. Under this circumstance, it is difficult to determine whether the behavior of BMSCs will be affected by the over-high O_2 level or H_2O_2. For this reason, we demonstrated the positive effects of H_2O_2-driven oxygenation on the proliferation, survival and osteogenic differentiation of BMSCs, where the concentration of O_2 was higher than that of in the PBS, PBS+H_2O_2, and Gel+H_2O_2 groups. In addition, previous literatures have proved that the amount of oxygen production catalyzed by enzyme/materials at the concentration of H_2O_2 (100 μM) will not affect the activity of cells in vitro experiments [*ACS Nano* **2019**, 13, 3206−3217; *Nano Lett.* **2020**, 20, 5149−5158; *Sci. Adv.* **2021**, 7, eabj0153]. Keeping the reviewer's comment in mind, we further supplemented the immunofluorescence evaluation of HIF-1α expression in vivo (Fig. 6 and Supplementary Fig. 21). These observations suggest that the level of H_2O_2-driven oxygenation could alleviate hypoxia, which further protected the implanted BMSCs from ROS and hypoxia-mediated death without side effects on BMSCs.

Reference 1: *ACS Nano* **2019**, 13, 3206−3217

manganese ferrite NPs (Figure 2f). Moreover, we confirmed that the synergistic O_2 generation by MFC-MSNs also occurs under low H_2O_2 concentration such as 100 μM, which is typical concentration with which to induce the inflammatory condition *in vitro* (Figure S8).[56,57] After 24 h, only negligible amounts of Mn, Fe, and Ce ions were leached as determined by ICP-MS analysis, demonstrating the stability of MFC-MSNs under inflammatory physiological conditions.

Reference 2: *Nano Lett.* **2020**, *20*, 5149−5158

Moreover, the protective function of our FEM hydrogel to fibroblasts against the noxious ROS was also investigated under the pathological oxidative microenvironment (100 μM H_2O_2). We first looked into the intracellular ROS variations through a ROS indicator DCFH-DA.[45] Fibroblasts coincubated with

Reference 3: *Sci. Adv.* **2021**, 7, eabj0153

To evaluate the efficacy of the hydrogel in protecting skin cell survival under pathological oxidative stress, HaCaT cells were seeded on the hydrogel surface in the presence of 100 µM H_2O_2, and the cell viability was quantified (Fig. 3G). The non–ROS-responsive hydrogel was used as a control. We found that HaCaT cells seeded on the hydrogel did not undergo substantial apoptosis in 100 µM H_2O_2 at 48 hours and even proliferated at 72 hours. However, HaCaT

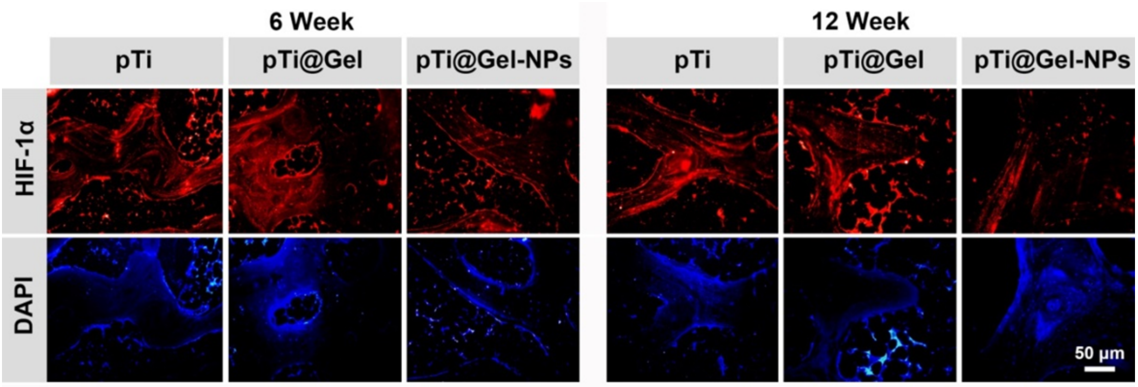

Fig. 6 (e) Representative immunofluorescence staining images of HIF-1α on the bone tissue around the scaffolds at week 6 and 12 after different treatments.

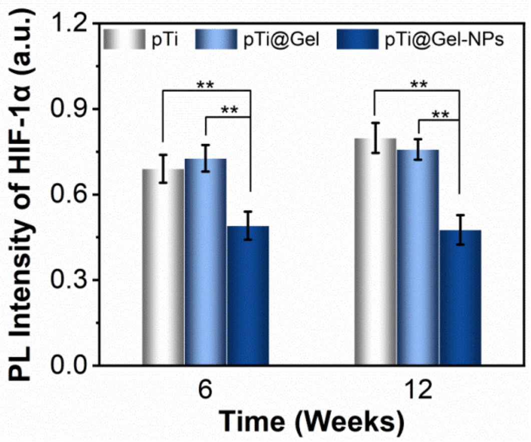

Supplementary Fig. 21. Quantitative statistics of HIF-1α on the bone tissue around the scaffolds at week 6 and 12 after different treatments (n = 3 independent experiments).

7. In Fig. 3, the concentration of H_2O_2 was demonstrated as 100 µM. It is suggested to use the uniform unit (0.1 M or 100 µM) for the convenience of readers. Additionally, why this concentration of H_2O_2 was selected for the evaluation? Is this concentration can represent the ROS levels in RA?

Authors' responses: Thanks a lot for the reviewer's comments. For the convenience of readers, we have unified the unit H_2O_2 as 0.1 mM in the revised manuscript. In addition, we performed the in vitro proliferation and osteogenic differentiation of BMSCs under low H_2O_2 concentrations such as 0.1 mM. According to literatures, this concentration is usually applied for cell experiments under the pathological inflammatory conditions, which is also the typical concentration with which to trigger the inflammatory condition in vitro.[1, 2] Moreover, the extracellular concentration of H_2O_2, as the most representative ROS, reached at a

concentration of 1.0 mM under pathological inflammatory conditions, which is estimated to be up to a 100-fold higher than healthy tissue. Although the concentration of H_2O_2 we selected for in vitro experiments is lower than the ROS levels in RA, the results can provide evidence that nanozyme-reinforced hydrogel possesses in vitro ROS scavenge performance. Due to the excellent catalytic durability of ε-PLE@MnCoO/Gel hydrogel, it is able to persistently produce O_2 as long as the presence of over-expressed H_2O_2. Moreover, the problem of over-high O_2 levels should be avoided due to its complex effects on the behavior of BMSCs.

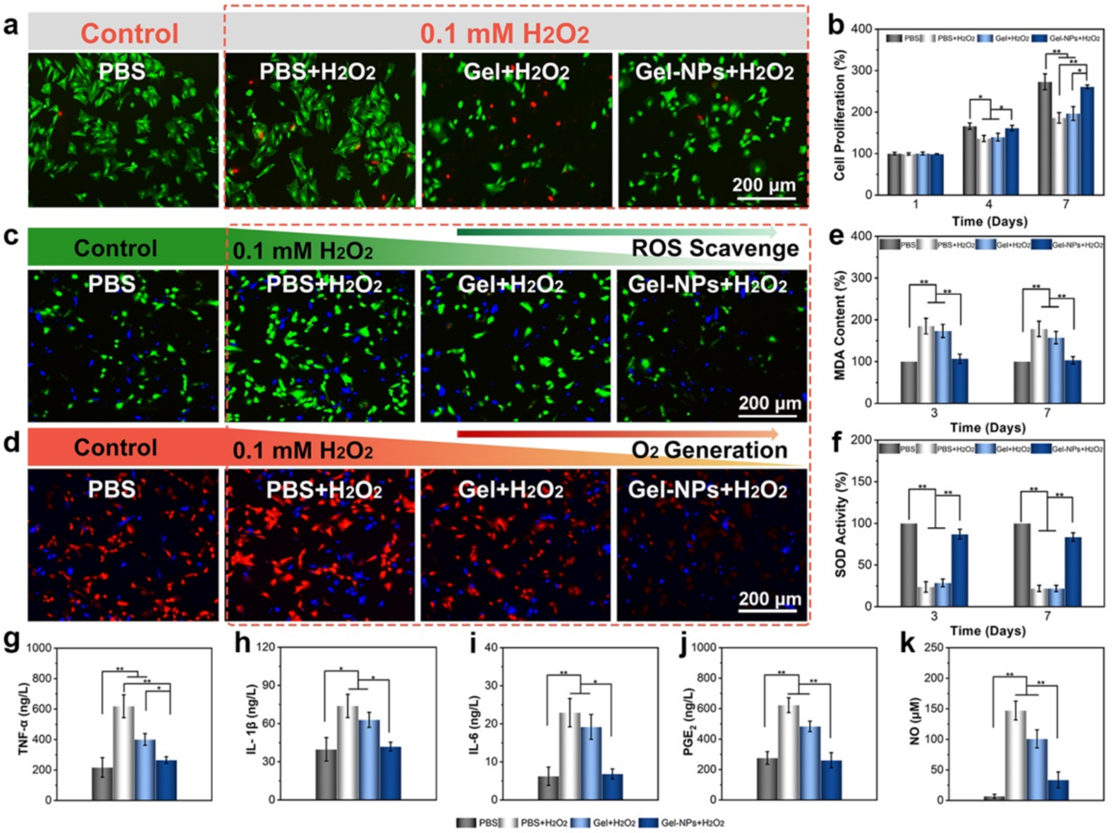

Fig. 4. **In vitro studies of BMSCs treated with the hydrogel.** (a) Calcein AM/propidium iodide (PI) staining of BMSCs after treatment with PBS, PBS+H_2O_2, Gel+H_2O_2, and Gel-NPs+H_2O_2. (b) Cell proliferation of BMSCs in different groups at 1st, 4th, and 7th day. (n = 3 independent experiments). (c) ROS scavenge ability validated by a ROS probe (DCFH-DA) after different treatments. Green fluorescence from DCFH-DA indicates the presence of ROS. (d) Intracellular O_2 generation assay monitored by an O_2 probe [$Ru(dpp)_3Cl_2$]. Red fluorescence from $Ru(dpp)_3Cl_2$ is quenched by O_2. (e) MDA activity of BMSCs after different treatments. (n = 3 independent experiments). (f) SOD activity of BMSCs after different treatments. (n = 3 independent experiments). (g-k) Expression of inflammatory mediators of BMSCs after different treatments including TNF-α (g), IL-1β (h), IL-6 (i), PGE_2 (j), and NO (k). (n = 3 independent experiments). *$P < 0.05$, **$P < 0.01$, and ***$P < 0.001$.

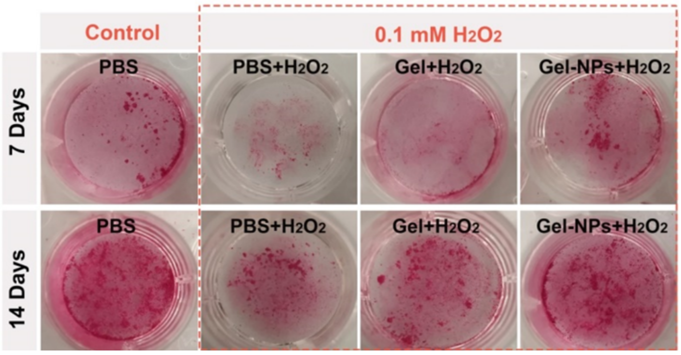

Fig. 5. (a) Gross observation of mineralized nodules stained by Alizarin Red. (b) Semi-quantitative analysis of mineralized nodules in different groups.

References

1. Kim, J., *et al*. Synergistic oxygen generation and reactive oxygen species scavenging by manganese ferrite/ceria co-decorated nanoparticles for rheumatoid arthritis treatment. *ACS Nano* **13**, 3206-3217 (2019).
2. Wang, S., *et al.* Nanoenzyme-reinforced injectable hydrogel for healing diabetic wounds infected with multidrug resistant bacteria. *Nano Lett.* **20**, 5149-5158 (2020).

8. Fig. 3D, the O_2 generation looks similar between the groups of PBS (control) and Gel+H_2O_2.

Authors' responses: Thanks a lot for the reviewer's comments. In our system, the oxygenation capability of the nanozyme-reinforced hydrogel was evaluated by a typical O_2 level indicator [Ru(dpp)$_3$Cl$_2$], whose red luminescence can be strongly quenched by oxygen. After incubation with H_2O_2 for 3 days, the nanozyme-reinforced hydrogel (Gel-NPs+H_2O_2 group) scavenged significantly more H_2O_2 and produced markedly more O_2 than the PBS, PBS+H_2O_2, and Gel+H_2O_2 groups. For the Gel+H_2O_2 group, it did not have the ability to catalyse H_2O_2 into O_2. Therefore, the O_2 level was similar to that of the PBS group.

9. Fig. 5, comparisons to evaluate therapeutic effects of using pTi@Gel-NPs without loading BMSCs and using BMSCs alone without the gel are suggested.

Authors' responses: In this study, 3D printed porous titanium alloy scaffolds were used as mechanical support, and three groups of as-prepared scaffolds (pTi, pTi@Gel, pTi@Gel-NPs) loaded with BMSCs (at a density of 2×10^6 cells/well) were transplanted into the bone defects, respectively. Therefore, the group of BMSCs alone without the gel is the pTi group already existed in the system.

It is worth mentioning that, in fact, we set up the pTi@Gel-NPs without loading BMSCs group in the exploratory experiment and conducted a preliminary evaluation of bone regeneration. As shown in the figures below, the effect of bone regeneration was poor when there was no stem cell delivery in RA bone defect. This may be due to the limited number of endogenous BMSCs in RA environment. Although hydrogels can improve the harsh microenvironment of RA, they lack enough seed cells to play a full role in osteogenesis. In general, the effect of bone regeneration obtained by using pTi@Gel-NPs without loading

BMSCs was similar to that of the pTi and pTi@Gel groups, and they were inferior to the pTi@Gel-NPs group (**Fig. 3 to Response**).

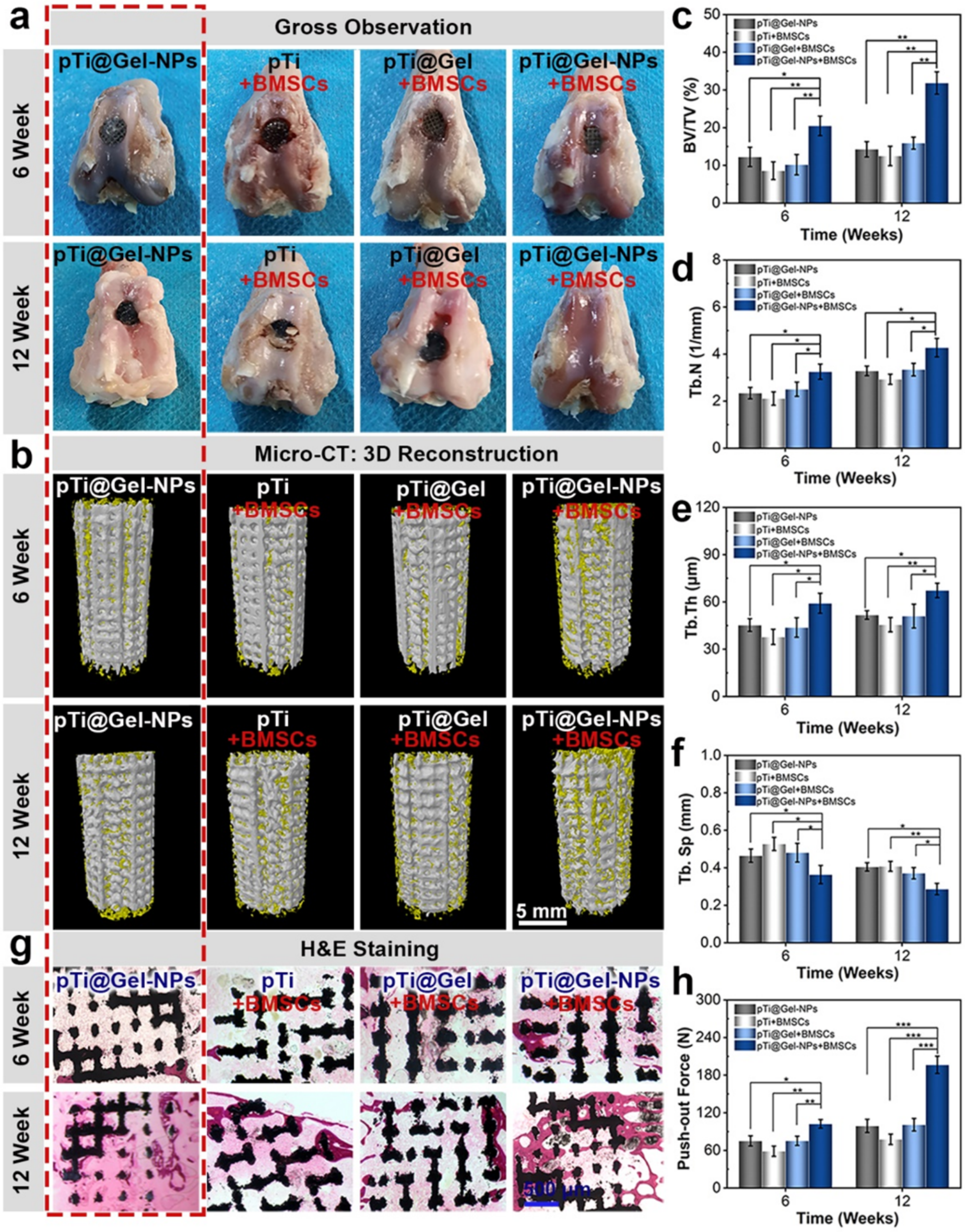

Fig. 3 to Response: (a) Gross appearance for the articular surface of distal femurs at weeks 6 and 12 after the treatment of pTi@Gel-NPs without BMSCs, pTi, pTi@Gel, and pTi@Gel-NPs scaffolds. (b) Representative 3D reconstruction images of bone regeneration from different scaffold treatments. (c-f) Quantitative statistics of BV/TV (c), Tb.N (d), Tb.Th (e), and Tb.Sp (f) from different groups according to Micro-CT scanning. (g) H&E staining of bone defects at weeks 6 and 12 after the treatment. (h) Quantitative analysis of the osseointegration according to biomechanical pull-out test.

Stem cell-based therapy has drawn attention as an alternative option for RA management by virtue of its unique characteristics. However, the therapeutic efficacy of this

approach is seriously threatened by the poor oxygen supply and accumulated ROS in RA pathological microenvironment. To address these challenges, we developed a biological nanozyme-reinforced hydrogel with ROS scavenging and oxygen generation synergistically to be utilized as a delivery vehicle of BMSCs. After transplanted in vivo, the designed hydrogels could protect implanted cells from ROS and hypoxia-mediated death, favourably enhancing the therapeutic efficacy of stem cells. Therefore, we tried to focus on the repair effect of delivering BMSCs into RA bone defects, so pTi@Gel-NPs without loading BMSCs was not the scope of this study. For these reasons, we chose not to set up this group.

10. Will the loaded BMSCs be released from the gel in articulation? The reviewer feels that the therapeutic effect more attributes to the osteogenic bioactivity through the paracrine of MSCs instead of the osteogenic differentiation. More evidences to determine how encapsulated BMSCs facilitated the bone reconstruction are suggested.

Authors' responses: Thanks for the question. In our system, the engineered nanozyme-reinforced hydrogel not only acts as an injectable delivery vehicle of BMSCs, but also releases cells in articulation to exert immunomodulation and tissue repair properties. Structurally, the nanozyme-reinforced hydrogel disclosed a homogenous porous structure with average pore diameters at 200 μm. It was reported in research studies that scaffold materials with this range of nanochannels could allow cell penetration, thereby enhancing cell migration and proliferation in the surrounding bone tissue. Moreover, the polymer used in this study was based on hyaluronic acid (HA). HA is a polysaccharide naturally found in synovial joint fluids, which was employed as the hydrogel backbone on account of its biocompatibility, biodegradability, bio-functionality, high-water retention, and viscoelastic properties. According to the reviewer's valuable advice, we further demonstrated that the nanozyme-reinforced hydrogel would be degraded nearly 75% on the day 24 days, which also implied that the loaded BMSCs could release from the hydrogel in vivo. Functionally, the nanozyme-reinforced hydrogel together with stem cells effectively suppressed inflammatory cytokines and improved bone regeneration after 3 months, and the released BMSCs were mainly responsible for this satisfactory therapeutic effect after the degradation of hydrogels.

In addition, it is believed that the mechanism of the transplanted BMSCs to promote bone repair should be originated from the synergistic effect of osteogenic differentiation and paracrine mechanism. Paracrine action refers to various substances secreted by stem cells, including growth factors, cytokines, microRNAs, proteases and extracellular vesicles, which regulate cell differentiation, proliferation, migration, apoptosis, etc. In this study, for the reconstruction of bone defects, the paracrine effect of transplanted BMSCs still can promote bone regeneration through the mechanism of promoting osteogenic differentiation of stem cells and osteoblasts. According to Fig. 5, the nanozyme-reinforced hydrogel as a H_2O_2-driven oxygenator could promote the osteogenic differentiation of BMSCs by enhancing mineralized nodule deposition and up-regulating the expression of osteogenic related markers (including ALP, BMP-2, RUNX-2, and OCN) in vitro. Considering the reviewer's suggestion, we have supplemented PCR detection on the collected bone tissues to further demonstrate how

encapsulated BMSCs promote bone reconstruction. The results in Supplementary Fig. 25 showed that the up-regulated osteogenic related genes (including ALP, BMP-2, RUNX-2, and OCN) revealed that the osteogenic differentiation of stem cells was a potential mechanism to promote bone reconstruction. Moreover, we have supplemented the Safranin O-fixed green staining of cartilage tissue around the scaffolds. Consisted with the results of gross observation, the pTi@Gel-NPs group possessed higher levels of chondrocytes and cartilage matrix compared to that in pTi and pTi@Gel groups, demonstrating the nanozyme-reinforced hydrogel could promote cartilage matrix distribution (Supplementary Fig. 24). Therefore, these results provide compelling evidence to demonstrate that encapsulated BMSCs facilitate bone reconstruction, and the osteogenic differentiation and paracrine effect of transplanted BMSCs are involved in the mechanism of bone repair.

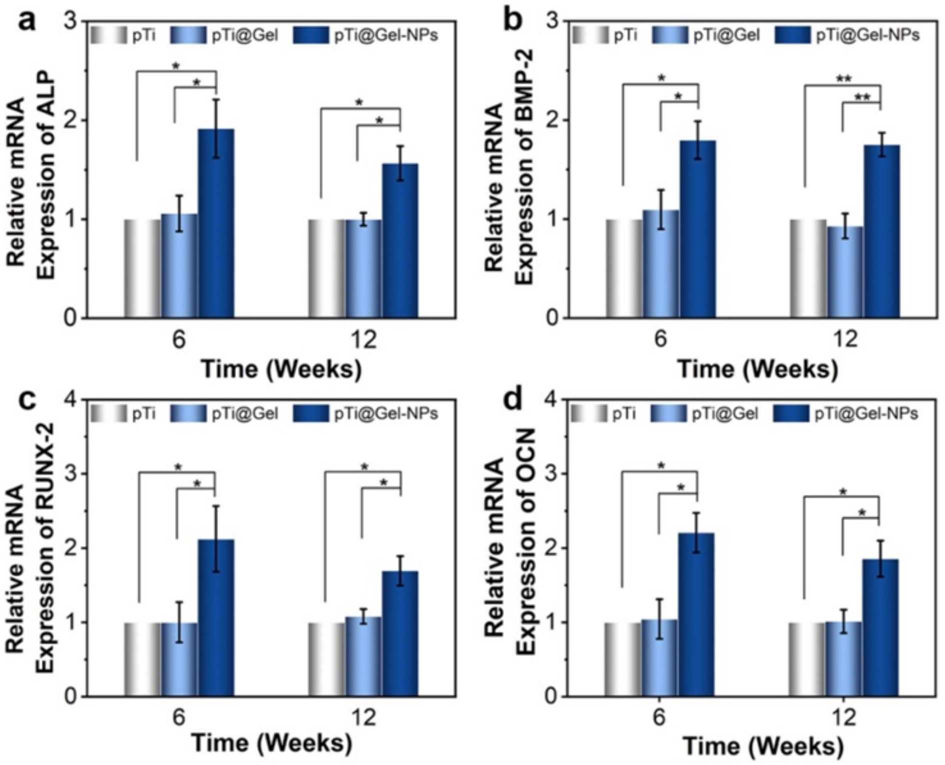

Supplementary Fig. 25. Relative mRNA expression levels of osteoclastic genes from bone defects at week 6 and 12 after the treatment with pTi, pTi@Gel, and pTi@Gel-NPs scaffolds, including ALP (a), BMP-2 (b), RUNX-2 (c), and OCN (d) (n = 3 independent experiments). *P < 0.05, **P < 0.01, and ***P < 0.001.

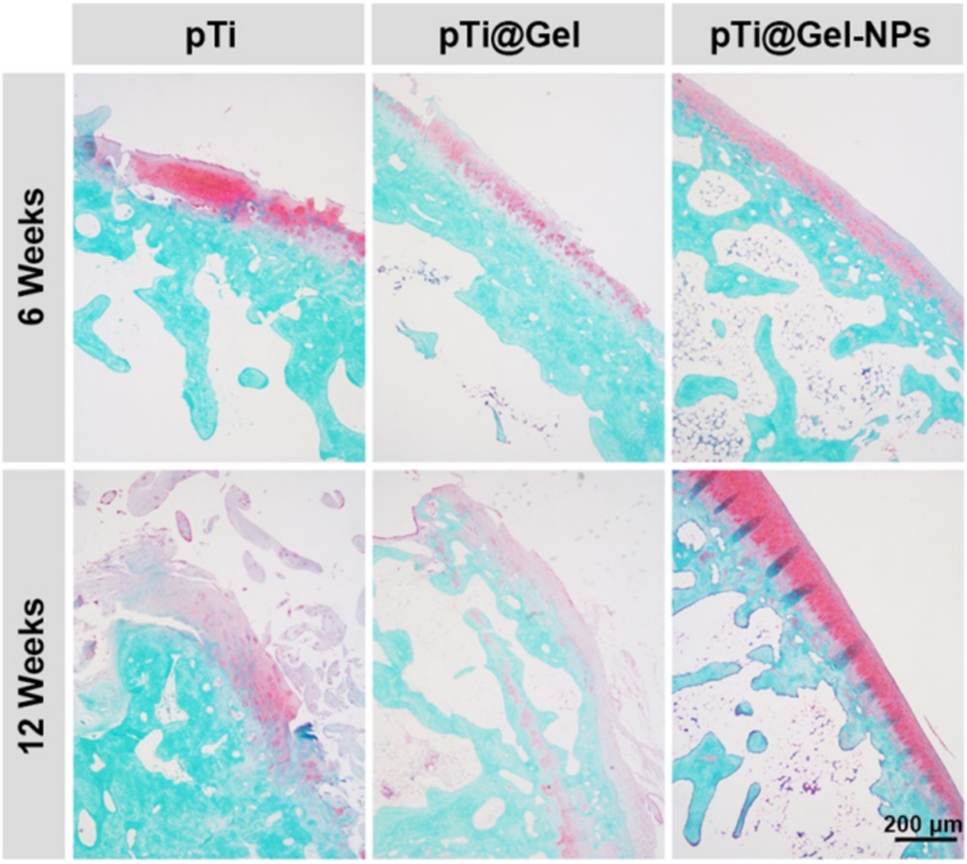

Supplementary Fig. 24. Safranin O-fixed green staining on the cartilage surface at week 6 and 12 after the treatment with pTi, pTi@Gel, and pTi@Gel-NPs scaffolds.

11. Authors only evaluated the local inflammatory levels. Will this strategy also affect the systemic immune response, especially considering RA is caused by autoimmune disorders?

Authors' responses: Thanks for the question. RA is a progressive arthritic disease, and the full recovery of RA remains a huge challenge for clinicians and researchers. A well-established hallmark of RA is the pathological inflammation associated with a large number of molecules, while the inhibition of one or a few molecules may not be sufficient to halt or reverse disease progression. In our pre-experiments, we detected the expression of inflammatory factors of TNF-α, IL-1β, IL-6, and PGE_2 at the serum level. Although the results showed a slight decrease in the expression of systemic inflammatory factors, it did not show a significant impact on the systemic immune response as demonstrated by the supplemented data (Supplementary Fig. 23) on the systemic immune response.

Thus, the engineered nanozyme-reinforced hydrogel encapsulated with BMSCs can prominently alleviate the symptoms of RA, including suppression of inflammatory cytokines and improvement of bone regeneration. In addition, it was demonstrated that the cell-laden nanozyme-reinforced hydrogel is a powerful system to reduce the loosening, displacement, and periprosthetic fractures after prosthesis implantation in RA. Our work represents a promising approach for the mediation of stem cell therapy, which offers a vast application prospect for the intervention of other immune-related diseases even beyond RA.

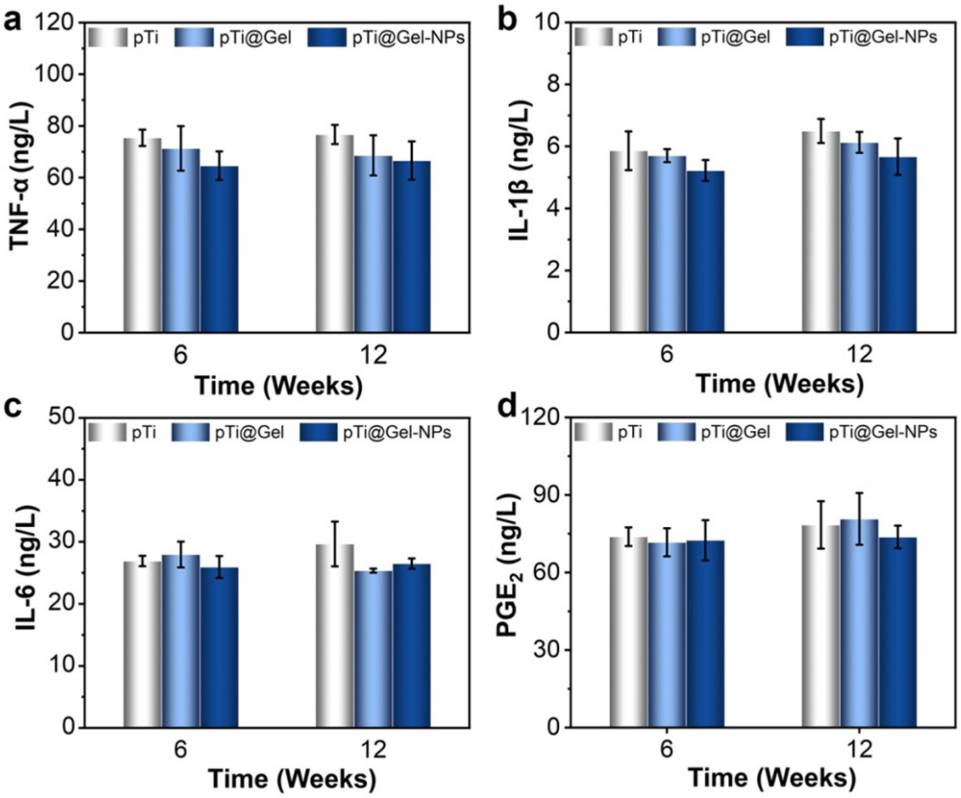

Supplementary Fig. 23. Content of inflammatory cytokines in serum, including TNF-α (A), IL-1β (B), IL-6 (C), and PGE_2 (D) to assess systemic RA inflammatory state (n = 3 independent experiments).

Minor suggestions:

1. It is suggested to say "… was investigated" instead of "… was systematically investigated". Each result should be systematically investigated.

Authors' responses: It is really true as the reviewer suggested that each "… was systematically investigated" should be changed into "… was investigated". In the revised manuscript, we have re-written these sentences according to the Reviewer's suggestion.

2. It is suggested to show the repeats number of each data directly in figure legends.

Authors' responses: We sincerely thank the reviewer for providing helpful comments, and the repeats number of each data was provided in the revised manuscript according to the reviewer's comments.

Reviewer #2 (Remarks to the Author):

Nanozymes are functional nanomaterials with enzyme mimicking activities. They have been explored for various disease therapy. In the manuscript, the authors developed a novel nanozyme-reinforced hydrogel, which could alleviate the symptoms of RA by regulating inflammatory cytokines and improved the bone regeneration in the in vivo model. This work has broadened the biomedical applications of nanozymes and would inspire others to explore the wide promise of nanozymes as well as other bionanomaterials. Therefore, I would suggest the publication after addressing the following concerns.

Authors' responses: We thank the reviewer for the useful comments and suggestions. We have revised the manuscript accordingly.

1. Why did the authors select MnCoO nanozyme as the catalase-mimic? How about other nanozyme with catalase-like activity? How about the stability of MnCoO nanozyme? Would Mn^{2+} and Co^{2+} be released from the MnCoO nanozyme?

Authors' responses: Thanks for the questions. In our previous studies, we demonstrated that the mesoporous manganese cobalt oxide ($Mn_{1.8}Co_{1.2}O_4$ = MnCoO) nanozyme derived from $Mn_3[Co(CN)_6]_2$ metal-organic frameworks (MOFs) simultaneously exhibited endogenous H_2O_2 decomposition and oxygen generation without self-consumption or external activation. Compared to the existing technology, the MnCoO nanozymes exhibited negligible biological toxicity, robust catalytic ability, and outstanding physiological stability. For example, when MnCoO nanozymes were incubated in saline, phosphate-buffered saline, and Dulbecco's modified eagle medium even for 10 months, they were still intact without obvious degradation or release of metal ions.

The direct supplementation of natural catalase often has no effect on stem cells, since the hostile microenvironment of RA greatly inhibits the expression of these antioxidant biological enzymes. Moreover, natural catalase, as a type of protein, is often associated with intrinsic disadvantages such as low operational stability and easy degradation by protease. The problems of high cost for preparation and purification as well as difficulties in recycling and reusing are also formidable. Therefore, these shortcomings all limit their applications as a H_2O_2-driven oxygenerator to regulate stem cell behavior in our system. In addition to natural catalase, several previous reports have developed artificial nanozymes with the catalase-like activity that is locally implanted for the management of orthopedic diseases. These H_2O_2-driven oxygenerators are mainly based on Cerium oxide, MnO_2, CaO_2, Sliver nanozymes, and carbon nanomaterials. Although these reported nanozymes exhibited outstanding catalytic activity for in situ endogenous O_2 generation, some major shortcomings associated with them overshadow the brilliance of therapeutic effect. For example, Cerium oxide nanoparticles have the properties of catalase-like enzymes and efficiently scavenge H_2O_2, whereas in the meantime they have the properties of superoxide dismutase to produce toxic H_2O_2. This type of self-cascading antioxidant nanozyme is difficult to balance the H_2O_2 generation and elimination. As far as MnO_2 and CaO_2 based nanomaterials concerned, an inevitable disadvantage is their rapid pH-responsive degradation, while sustained catalytic ability and

long-time durability of catalysts are preferred during practical applications. Meanwhile, the toxicity and side effects of some developed nanoparticles is unpredictable. Therefore, the developed MnCoO nanozymes exhibited good physiological stability and negligible biological toxicity that promises continuously scavenge H_2O_2 and produce O_2 in vivo.

2. What's the concentration of H_2O_2 in RA pathological microenvironment? Why did the authors chose 1M H_2O_2 to test the catalase-like catalytic activity?

Authors' responses: Thanks for the comments. In the RA pathological microenvironment, oxidative stress is regarded as a crucial mechanism in the initial and progressed phases of destructive proliferative synovitis. According to the previous reports, the extracellular concentration of H_2O_2, as the most representative ROS, reached at a concentration of 1.0 mM under pathological inflammatory conditions, which was estimated to be up to 100-fold higher than healthy tissue.[1-3]

In addition, we selected 1 mM H_2O_2 to test the catalase-like catalytic activity rather than 1 M as indicated in the section of Methods. The concentration was chosen for imitating the H_2O_2-rich RA hostile microenvironment, since the H_2O_2 level has been described as high as 1.0 mM under pathological inflammatory conditions. We are very sorry for our mistakes and have changed it into right.

References:

1. Peiró Cadahía, J., *et al.* Synthesis and evaluation of hydrogen peroxide sensitive prodrugs of methotrexate and aminopterin for the treatment of rheumatoid arthritis. *J. Med. Chem.* **61**, 3503-3515 (2018).

2. Kumar, R., *et al.* Mitochondrial induced and self-monitored intrinsic apoptosis by antitumor theranostic prodrug: in vivo imaging and precise cancer treatment. *J. Am. Chem. Soc.* **136**, 17836-17843 (2014).

3. Weinstain, R., Savariar, E. N., Felsen, C. N., Tsien, R. Y. In vivo targeting of hydrogen peroxide by activatable cell-penetrating peptides. *J. Am. Chem. Soc.* **136**, 874-877 (2014).

3. It stated that the produced dissolved oxygen could improve the poor oxygen supply in RA pathological microenvironment. How about the expression levels of HIF-1 (hypoxia-inducible factor)?

Authors' responses: Thank you for your suggestions, which have helped improve our manuscript. To address your concerns, we have supplemented the immunofluorescence evaluation of HIF-1α expression in vivo experiments, and more data and discussions have been provided in the revised manuscript as well as listed as follows.

"On the other hand, hypoxia-inducible factors 1α (HIF-1α), as a marker of tissue hypoxia, is highly expressed in the hypoxic environment of RA joints. The hypoxia-attenuating ability of nanozyme-reinforced hydrogel was then verified by immunofluorescence staining of HIF-1α. According to Fig. 6e and Supplementary Fig. 21, the downregulation in HIF-1α expression was most prominent after treatment with the pTi@Gel-NPs group, implicating its simultaneous

inhibitory of HIF-1α signaling pathways and synergistic production of O_2. Taken together, these results demonstrated the superior antioxidation and hypoxic reliever properties of the nanozyme-reinforced hydrogel in vivo, which has the potential to act as an advanced stem cell delivery vehicle in the management of hostile microenvironment."

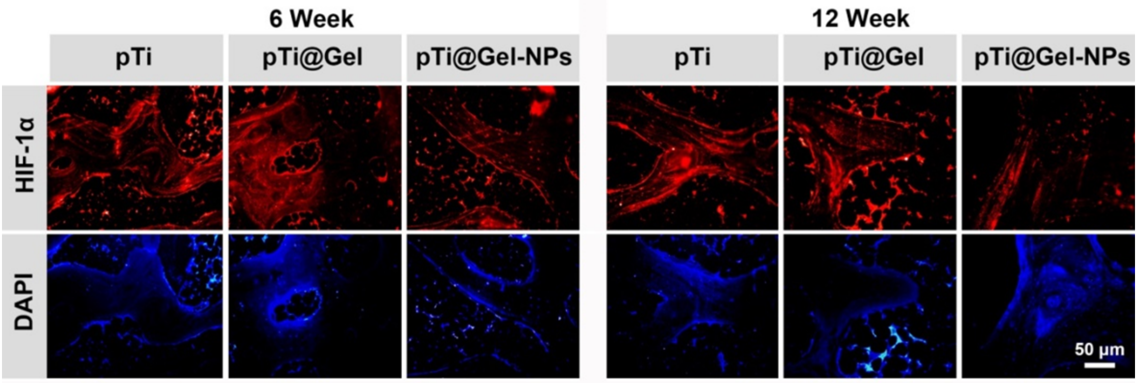

Fig. 6 (e) Representative immunofluorescence staining images of HIF-1α on the bone tissue around the scaffolds at weeks 6 and 12 after different treatments.

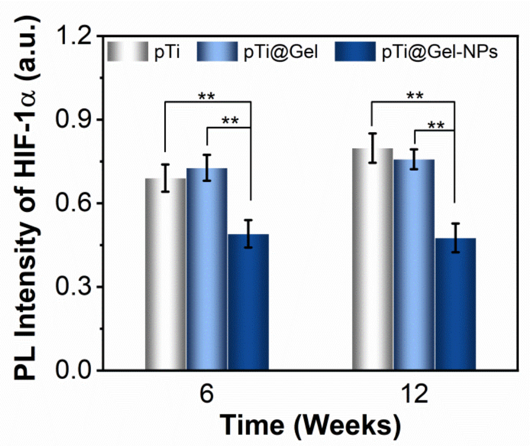

Supplementary Fig. 21. Quantitative statistics of HIF-1α on the bone tissue around the scaffolds at week 6 and 12 after different treatments (n = 3 independent experiments).

4. In RA model, the serious joint swelling behaviors would be observed. How about the improvement effect?

Authors' responses: The swelling of peripheral joint is one of the most typical local syndromes of RA. In our system, once the rabbits developed typical symptoms of RA, such as the elevated joint surface temperature and swollen joint, in situ implantations of pTi, pTi@Gel, and pTi@Gel-NPs scaffolds were performed, respectively. At predetermined time intervals, the joint surface diameter was recorded for evaluation of the joint swelling situation (Supplementary Fig. 20). It could be observed that the joint diameter of all experimental groups was significantly increased after injection of the model drug, and achieved peak values at 6 weeks after surgery due to the incision stimulation. As the implantation time further extended to 12 weeks, the joint diameter of all groups was gradually reduced to a minimum. In particular, the most significant improvement of local syndromes of RA was observed in the pTi@Gel-NPs group, whose joint surface diameter achieved the distinct reduction. These

results demonstrated that the pTi@Gel-NPs group could greatly inhibit the serious joint swelling behaviors, relieve the local excessive inflammation, and prevent synovial hyperplasia of the joint.

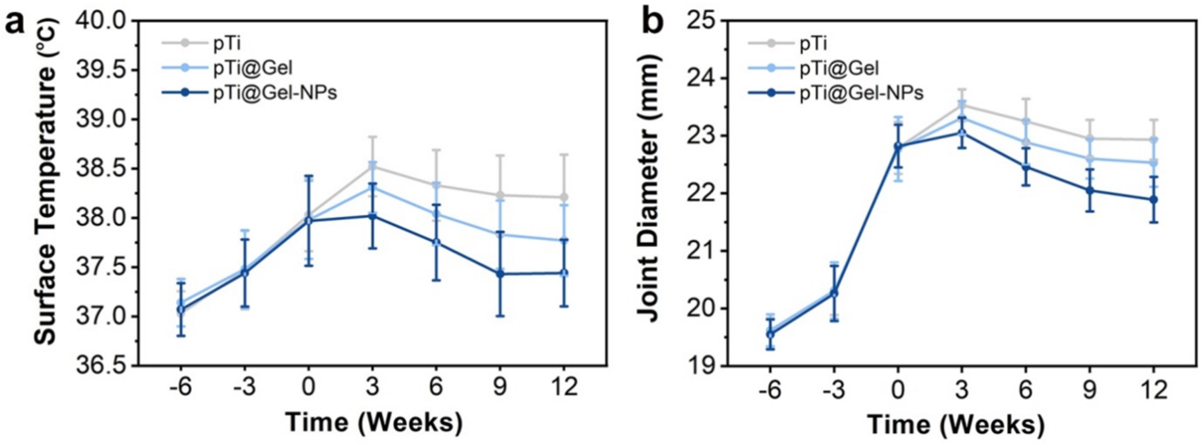

Supplementary Fig. 20. **Skin temperature and joint diameter studies.** (a) Skin temperature and (b) joint diameter of RA rabbit implanted with various samples recorded during the whole therapeutic period. (n = 10 independent experiments).

5. To exhibit the regenerated cartilage better and articular morphology, the Safranin-fixed green staining of joints could be performed.

Authors' responses: Thank the reviewer for the constructive comments and suggestions. We have supplemented the Safranin O-fixed green staining of cartilage tissue around the scaffolds, and more data and descriptions have been included in the revised manuscript as well as listed as follows.

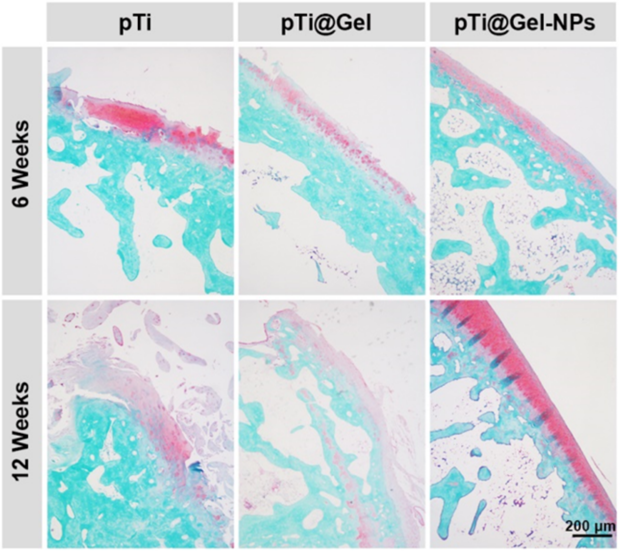

Supplementary Fig. 24. Safranin O-fixed green staining on the cartilage surface at weeks 6 and 12 after the treatment with pTi, pTi@Gel, and pTi@Gel-NPs scaffolds.

"To identify the cartilage formation, Safranin O-fixed green staining of tissues around the scaffolds was applied. Consisted with the results of gross observation, the pTi@Gel-NPs group possessed higher levels of chondrocytes and cartilage matrix compared to that in pTi and

pTi@Gel groups, demonstrating the nanozyme-reinforced hydrogel could promote cartilage matrix distribution (Supplementary Fig. 24)."

6. Since it is a study on nanozymes, several closely related publications could be cited if possible. For example,

(a) Chemical Society Reviews, 2019, 48, 1004-1079.

(b) Nano Letters, 2022, 22, 508-516.

Authors' responses: As you suggested, some highly relevant references about advanced nanozymes for biological applications were now included in the revised manuscript. Specific references are listed as follows:

Relevant References:

23. Lin, A., *et al.* Self-cascade uricase/catalase mimics alleviate acute gout. *Nano Lett.* **22**, 508-516 (2022).

24. Wu, J., *et al.* Nanomaterials with enzyme-like characteristics (nanozymes): next-generation artificial enzymes (II). *Chem. Soc Rev.* **48**, 1004-1076 (2019).

REVIEWER COMMENTS

Reviewer #1 (Remarks to the Author):

Although authors addressed most of concerns from the reviewer, there are still several issues remain.

1. Animal model is confusing. If you make a bone defect with a bone drill, your model should be osteoarthritis. Rabbit is not a common animal for RA model. RA model will not have a specific big bone defect, which is not suitable for your formulation and application.
2. Authors make the bone defect and implant the scaffold at the same time. This is not a common operation and is not the common case for RA and OA in clinical setting. Please explain and state its influence to your treatment efficacy.
3. What is the thickness of cartilage? If your scaffold is thicker than the cartilage, how can stem cells in the same scaffold differentiate into both cartilage and bone, please hypothesize and explain. Most your bioevaluation focus on bone regeneration that is not the most important issue for RA. Bone erosion is a late symptom for RA. If you want to prove your efficacy on RA treatment, your focus is improper.
4. Scaffold implantation to a specific bone defect is a local treatment while RA is a systemic disease.
5. Figure 6 is hard to support the antioxidative and hypoxia-attenuating capacities except the factor HIF-α. ROS and hypoxia are inducers for inflammation but the inflammation reduction does not equal to the reduction of ROS and hypoxia. To support this, you have to evaluate ROS and other factors in the microenvironment.
6. In figure S24, does the cartilage lay inside your bone defect, around the bone defect, or far away from the bone defect? It is suggested you present normal cartilage/bone tissue, your repaired cartilage/bone tissue, and other RA cartilage/bone tissue that is away from your bone defect to show a whole picture. In figure S24, no significant difference is seen for bone regeneration. But your previous evaluation proved a better efficacy of your final formulation for bone regeneration. Please explain. What is the relationship between bone and cartilage regeneration? What is your formulation's function for both of them? Please explain.
7. What is the size of bone defect? Did auhtors implant the scaffold in its dry form or wet form? If in dry form, have you considered the swelling issue? Will the swelled scaffold go to other place after implantation and bring stem cells to other place to form bone?
8. Figure 7g seems inconsistent to figure S24, please explain.

Reviewer #2 (Remarks to the Author):

The authors have fully addressed my concerns. The revised manuscript can be published now.

Reviewer #1 (Remarks to the Author):

Although authors addressed most of concerns from the reviewer, there are still several issues remain.

1. Animal model is confusing. If you make a bone defect with a bone drill, your model should be osteoarthritis. Rabbit is not a common animal for RA model. RA model will not have a specific big bone defect, which is not suitable for your formulation and application.

Authors' responses: Thanks for your comments. We apologize for confusing you by not emphasizing the relationship between bone defect and RA, and we have revised the manuscript to address your concerns and hope that it is now clearer. RA often leads to severe late complications such as joint pain, stiffness, and loss of function. Especially in the knee joint, the limited mobility may ultimately require joint replacement surgery to restore function and relieve pain. However, the bone regeneration capacity of RA patients is insufficient as the innate healing process is impaired by reactive oxygen species and intense inflammation, resulting in poor integration of the host bone tissue with the implanted prosthesis. Ultimately, poor osseointegration causes postoperative complications such as prosthesis loosening and displacement. Therefore, it is an urgent clinical problem to improve the bone regeneration and integration abilities after joint replacement to avoid postoperative complications. In this study, we constructed a bioactive prosthesis using nanozyme-reinforced hydrogel in combination with 3D printed microporous titanium alloy scaffold. As a H_2O_2-driven oxygenator, the hydrogel can regulate stem cell behavior and further promote bone regeneration and osseointegration at the host bone-prosthesis interface. Therefore, we used a bone drill to prepare a specific big bone defect matching the implanted prosthesis, which simulates the osteotomy and prosthesis implantation procedure of joint replacement.

As for the animal species for preparing the arthritis model, bone tissue engineering related animal models can be divided into three categories. (1) High throughput "fundamentals" studies favor classically small-sized species such as mouse, hamster and rat. These species are the staple elements for early-stage in vivo models, which have advantages of ease of handling, low costs, and ready availability through commercial channels. However, short study duration and notable dissimilarities biomechanically and physiologically are issues to consider. (2) Comprehensive, longer-term bioactivity and feasibility research typically uses medium-sized species such as rabbit and dog. Models in this category are suitable for research that requires increase in defect volumes, a scale-up in the body mass, and marginal scope for incorporating application specific scenarios. Nevertheless, quadrupedal gait, poor experimental tolerance and shortage of analogous secondary bone remodeling limit the clinical relevance of outcomes. (3) Late-translational stage clinical modeling is almost exclusively the domain of ovine models, while bovine and porcine species are not very common animal models. Models in this category are developed for trial a solution in consideration of as close as is

possible to its clinical application. Although this category is most accurate for assessing clinical efficacy, its associated problems such as high cost, restricted study number, and complicated infrastructural requirements are issues to consider.

In our system, we aim to observe the osseointegration between the host bone and the prosthesis after joint replacement using the rabbit animal model. Compared with small-sized species such as mice, midsized rabbit in vivo models can provide considerable stabilities biomechanically and physiologically and long-term bioactivity. In particular, rabbit animal models increase defect volumes, while the bone volume of mice is too small to accommodate the implantation of the prosthesis. Although the rabbit animal model cannot inform one to the same degree of accuracy as large mammal studies do, the rabbit is suitable for a preclinical trial model. Therefore, the rabbit arthritis model is often involved, including RA and degenerative arthritis, and we selected rabbits for the RA model.

2. Authors make the bone defect and implant the scaffold at the same time. This is not a common operation and is not the common case for RA and OA in clinical setting. Please explain and state its influence to your treatment efficacy.

Authors' responses: In RA, structural joint damage as a consequence of synovitis is commonly seen. Typically, bones are affected, resulting in erosions and loss of cartilage, which eventually causes joint deformities and functional impairments. Without proper control, an increase in the risk of fracture has been reported in RA patients and some of them ultimately have to undergo joint replacement surgery to restore function and relieve pain in clinics. The 3D printed microporous titanium alloy scaffolds, one of the most popular inorganic orthopaedic prostheses, can provide appropriate mechanical support and induce bone ingrowth. In our work, we aimed to improve the osseointegration between the host bone and the prosthesis after joint replacement in RA. Therefore, we made the bone defect and implanted the scaffold at the same time, simulating the osteotomy and prosthesis implantation procedure of joint replacement. This is a real simulation of the operating procedures in clinical surgery and will not affect the treatment efficacy of bone integration.

3. What is the thickness of cartilage? If your scaffold is thicker than the cartilage, how can stem cells in the same scaffold differentiate into both cartilage and bone, please hypothesize and explain. Most your bioevaluation focus on bone regeneration that is not the most important issue for RA. Bone erosion is a late symptom for RA. If you want to prove your efficacy on RA treatment, your focus is improper.

Authors' responses: Thanks for your question. The thickness of rabbit knee joint cartilage is about 0.3-1.0 mm. The implanted scaffold is a 10 mm deep, 6 mm diameter cylindrical prosthesis, which is significantly thicker than the cartilage layer. Stem cells are not only related to differentiation capacity for differentiating into specific effector cells, such as osteoblasts, chondrocytes, nerve cells, and fibroblasts involved in tissue repair, but their paracrine effects

are also crucial. Among soluble active factors secreted by stem cells, there are cytokines, chemokines, interleukins (ILs), growth factors, adhesion molecules, hormones, and nucleic acids, as micro-RNAs (miRNAs), long non-coding RNAs (lncRNAs) or messenger RNAs (mRNAs), as well as extracellular vesicles (EVs). These bioactive factors have anti-inflammatory, immune-modulatory, and inducing cartilage regeneration properties that play a relevant role in the therapeutic potential of stem cells in cartilage injury.

As discussed before, the aim of our study is to improve the osseointegration between the host bone and the prosthesis after joint replacement in RA. Therefore, we focused on bone regeneration and in-growth in the micropores after prosthesis implantation, rather than the changes in systemic state or cartilage caused by RA. To clarify this point, we have emphasized the research purpose and made some changes in the revised manuscript. These changes will not influence the content and framework of the paper.

4. Scaffold implantation to a specific bone defect is a local treatment while RA is a systemic disease.

Authors' responses: Thanks for your comment. During the progression of RA, continuous inflammation leads to irreversible damage to the bone and tissue integrity. In particular, bone erosion in the knee joint contributes to limited mobility and loss of function, and some patients with severe cases of RA ultimately have to undergo joint replacement surgery to restore function and relieve pain in clinics. In this work, we mimicked the situation that patients developed severe RA and need to undergo joint replacement surgery, and hope to solve the problem of poor osseointegration after prosthesis implantation in RA, thus reducing postoperative complications such as prosthesis loosening and displacement. We focused on the orthopaedic problems of local bone integration after joint replacement, rather than the improvement of RA systemic status. In order to avoid misunderstanding, we have revised the related discussions and emphasized our research purpose in the revised manuscript.

5. Figure 6 is hard to support the antioxidative and hypoxia-attenuating capacities except the factor HIF-α. ROS and hypoxia are inducers for inflammation but the inflammation reduction does not equal to the reduction of ROS and hypoxia. To support this, you have to evaluate ROS and other factors in the microenvironment.

Authors' responses: Thank you for your suggestions, which have helped us improve the accuracy of our manuscript. To address your concerns, we have supplemented the immunofluorescence evaluation of ROS level in vivo experiments, and more data and discussions have been provided in the revised manuscript as well as listed as follows.

"Moreover, the ROS level in RA bone tissue represents the oxidative stress status during inflammation. We then evaluated the ROS variations through the ROS probe (DCFH-DA). As revealed by the images of immunofluorescence staining, the RA bone tissue treated with the pTi@Gel-NPs group displayed notably lower green fluorescence than that after pTi or pTi@Gel

treatment during the whole treatment cycle, demonstrating that the nanozyme-reinforced hydrogel could effectively reduce the level of ROS and attenuate oxidative stress in RA (Supplementary Fig. 21)."

Combined with the detection of 8-OHdG, 4-HNE, DCFH-DA, HIF-α, and inflammatory cytokines (TNF-α, IL-1β, IL-6, and PGE2) in bone tissue and synovial fluid, we think these results can well demonstrate the superior ROS scavenging and hypoxic reliever properties of the nanozyme-reinforced hydrogel in vivo, showing the potential to serve as an advanced stem cell delivery vehicle in the management of hostile microenvironment.

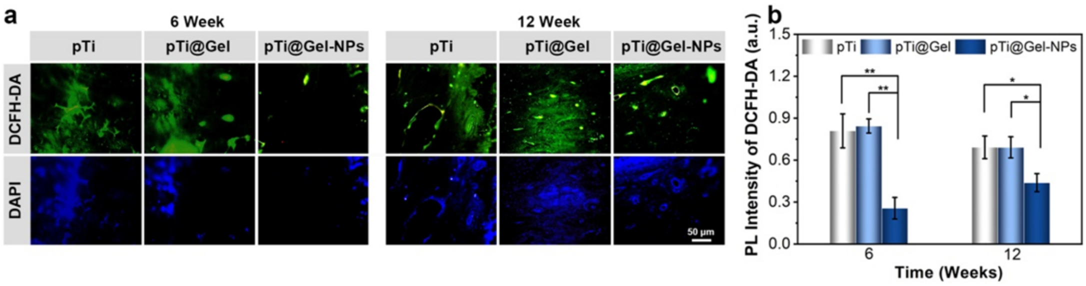

Supplementary Figure 21. The ROS level in RA bone tissue (a) Representative immunofluorescence staining images of DCFH-DA on the bone tissues around the scaffolds at weeks 6 and 12 after different treatments. (b) Quantitative statistics of DCFH-DA on the bone tissue around the scaffolds at week 6 and 12 after different treatments (n = 3 independent experiments).

6. In figure S24, does the cartilage lay inside your bone defect, around the bone defect, or far away from the bone defect? It is suggested you present normal cartilage/bone tissue, your repaired cartilage/bone tissue, and other RA cartilage/bone tissue that is away from your bone defect to show a whole picture.

Authors' responses: Thanks for your comment. In figure S24, the cartilage lay in the around of the bone defect, rather than inside or far away from the bone defect. We appreciate the reviewer's insightful suggestion and agree that it would be useful to demonstrate the regeneration of cartilage/bone tissue away from bone defects using different groups. However, we found that the conventional Safranin O-fixed green staining is difficult to perform in the presence of hard scaffold, and thus we first tried to push out the scaffold in the bone tissue and then decalcified the sample to avoid damaging the blade. During the process of titanium alloy scaffold pushing out, the cartilage inside the defect suffers severe damage, and even the surrounding bone may be broken. Therefore, only the cartilage morphology around the defect can be observed.

In figure S24, no significant difference is seen for bone regeneration. But your previous evaluation proved a better efficacy of your final formulation for bone regeneration. Please explain.

Authors' responses: For Figure 7, we observed the bone ingrowth inside the 3D printed

microporous prosthesis implanted in the bone defect, while Figure S24 showed the cartilage around the defect rather than the inside or surface of the scaffold. Moreover, after pTi and pTi@Gel treatments, the cartilage was still severely damaged, and the subchondral bone hyperplasia was reactive, resulting in a visual local increase in bone mass. For example, the bone mass of the whole distal femur was decreased in pTi group (Figure 7). However, the surface cartilage damage caused the reactive proliferation of subchondral bone in Figure S24, so it was found that the bone mass of this part seems to not decrease, or even increase. Therefore, the bone regeneration statue of Figure 7 and Figure S24 displayed a marked difference, and the bone regeneration capacity of Figure S24 could not represent the overall bone condition.

What is the relationship between bone and cartilage regeneration? What is your formulation's function for both of them? Please explain.

Authors' responses: In our system, the implanted bone marrow mesenchymal stem cells (BMSCs) play a crucial role in both bone and cartilage regeneration, which are known to differentiate towards bone, cartilage and fat tissues in a multilineage manner. Prior work has demonstrated that BMSCs not only possess potent anti-inflammatory and immunomodulatory properties, but also have superior abilities to differentiate into chondrocytes and osteoblasts. Moreover, BMSCs can exert paracrine effects to participate in bone repair. The bone tissue has a certain self-repair ability when it is damaged, while the bioactive factors secreted by the paracrine of stem cells can further accelerate the repair process. Thus, the stem cell implantation strategy has been applied for the treatment of inflammatory and degenerative rheumatic diseases, including RA.

In addition to stem cells, the application of a suitable scaffold biomaterial as stem cell carriers has gained intriguing results in maintaining the immune privileged and tissue repair capacities of stem cells. In our system, we developed an innovative concept of nanozyme-reinforced hydrogels as H_2O_2-driven oxygenator to regulate stem cell behavior. As three-dimensional polymeric networks, the developed hydrogels display hydrophilic nature, high-water content, as well as good substance permeability. In particular, the obtained hydrogel system could effectively decompose the endogenous H_2O_2 to produce O_2. The corresponding in vitro experiments demonstrated that the nanozyme-reinforced hydrogel could successfully assuage the hypoxic and oxidative microenvironment of RA, and thereby provided an appropriate 3D microenvironment for BMSCs proliferation and osteogenesis. Moreover, the polymer used in this study is originated from hyaluronic acid (HA). HA is a polysaccharide naturally found in synovial joint fluids. Several studies have demonstrated that HA is conducive to cell adhesion, and hydrogels prepared from pentanoate-modified HA can improve the osteogenic efficacy of bioactive substances and enhance bone formation capability. Therefore, stem cells together with nanozyme-reinforced hydrogels are important factors for both bone and cartilage regeneration, which can explain the phenomenon of osseointegration and cartilage repair that

we observed at the same time.

7. What is the size of bone defect? Did authors implant the scaffold in its dry form or wet form? If in dry form, have you considered the swelling issue? Will the swelled scaffold go to other place after implantation and bring stem cells to other place to form bone?

Authors' responses: Thank you for your question. The size of the bone defect is 10 mm deep, 6 mm in diameter matched with the size of a cylindrical 3D printed titanium alloy scaffold for simulating the large-scale defect during the precise osteotomy and prosthesis implantation steps of joint replacement. In order to steer stem cell behavior, we implanted the scaffold in its wet form. Wet hydrogels are very good extracellular matrix (ECM) biomimetic materials with three-dimensional crosslinked structures and high water contents, which can provide stem cells with structural support and environmental cues that influence biological processes. After implantation, the encapsulated BMSCs can move freely between the hydrogels, and further infiltrate and migrate into the surrounding bone tissue environment, thereby allowing inhibit inflammation and boosting the bone defect regeneration around the prosthetic interface. In addition, there's no need to concern about osteogenesis caused by stem cell migration elsewhere, since the normal bone tissue lacks the necessary initiation factors to promote the excessive osteogenic activity of stem cells. Moreover, the homing effect of transplanted stem cells to the microenvironment is associated with released signaling molecules at the injury site and receptors on the surface of MSCs, which enables the transplanted stem cells to fully exert suitable therapeutic effects in the local bone defect. Therefore, the nanozyme-reinforced hydrogel scaffold shows a potential for stem cell-based therapy application.

8. Figure 7g seems inconsistent to figure S24, please explain.

Authors' responses: Thanks a lot for the comments. In fact, Figure 7g and Figure S24 showed the different parts of the bone. In Figure 7g, we observed the bone ingrowth inside the 3D printed microporous prosthesis implanted in the bone defect by hard tissue staining, and the newly formed bone tissues at the bone-implant interface could be observed. In Figure S24, the Safranin O-fixed green staining should be operated by conventional soft tissue sectioning rather than hard tissue sectioning. In order to avoid damaging the blade, the 3D printed microporous scaffold in the bone tissue should be pushed out followed by decalcification. During the process of pushing out of metal scaffold, the cartilage inside the defect is difficult to keep intact, and even the surrounding bone may be broken. Thus, the cartilage around the defect is selected to observe the morphology rather than the inside. On the other hand, after pTi and pTi@Gel treatments, the cartilage was still severely damaged, and the subchondral bone hyperplasia was reactive, resulting in a visual local increase in bone mass. For example, the bone mass ingrowth into the microporous prosthesis was decreased in pTi group (Figure 7g). However, the surface cartilage damage caused the reactive proliferation of subchondral

bone in Figure S24, so it was found that the bone mass of this part seems to have not decreased, or even increased. Therefore, the bone regeneration capacities observed in Figure 7g and Figure S24 are inconsistent.

In fact, in the actual clinical joint replacement, the subject of concern is osseointegration of the prosthetic interface, and attention to the effect of the surrounding cartilage is dispensable. As shown in Figure R1 below, for the total knee replacement or partial knee replacement, the damaged articular cartilage is replaced by a smooth metal surface. The aim of our study is to improve the osseointegration between the host bone and the prosthesis after joint replacement in RA, and the regeneration of cartilage may not be an additional benefit to the life of the prosthesis after joint replacement. In order to avoid misunderstanding, we have revised the manuscript and deleted Figure S24.

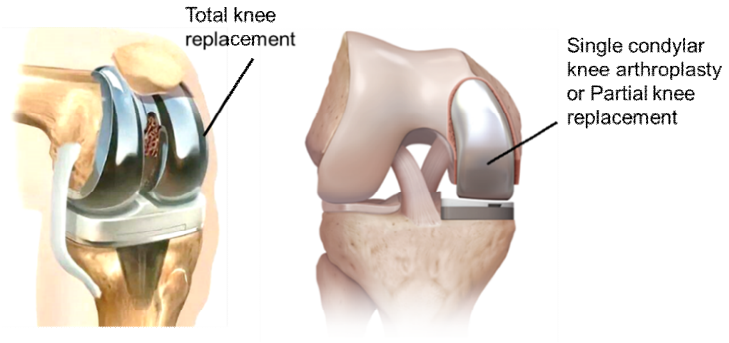

Figure R1. Schematic diagram after total knee replacement and partial knee replacement. The damaged articular cartilage is replaced by a smooth metal surface.

Reviewer #2 (Remarks to the Author):

The authors have fully addressed my concerns. The revised manuscript can be published now.

Authors' responses: We really appreciate the reviewer for the recommendation of publication.

REVIEWERS' COMMENTS

Reviewer #1 (Remarks to the Author):

The authors have answered my comments and the manuscript can be considered for publish.

Reviewer #1 (Remarks to the Author):

The authors have answered my comments and the manuscript can be considered for publish.

Authors' responses: We really appreciate the reviewer for the recommendation of publication.
